# From [NHC─H]^•^ to Persistent *σ*‐Complex Radicals: Photoinduced Radical Chemistry of Imidazolium Salts

**DOI:** 10.1002/anie.5517752

**Published:** 2026-04-29

**Authors:** Filipp M. Kolomeychuk, Lars J. C. van der Zee, Simon Mathew, Bas de Bruin, J. Chris Slootweg

**Affiliations:** ^1^ Van ’t Hoff Institute for Molecular Sciences University of Amsterdam Amsterdam Netherlands

**Keywords:** electron donor–acceptor complexes, EPR spectroscopy, imidazolium salts, *N*‐Heterocyclic Carbenes, radicals, single‐electron transfer

## Abstract

Imidazolium salts are ubiquitous precursors to *N*‐heterocyclic carbenes (NHCs), yet their radical chemistry remains largely unexplored, and structurally well‐defined imidazolium‐derived radicals are rare. Here, we show that visible‐light excitation of charge‐transfer (CT) bands of electron donor–acceptor (EDA) complexes between imidazolium cations and a triarylamine donor induces a single‐electron transfer. Variable‐temperature EPR spectroscopy reveals that irradiation at 100 K affords neutral [NHC─H]^•^ radicals, which undergo protonation upon warming to room temperature to give the persistent *σ*‐complex cations [IDippH_2_]^•+^ and [IXylH_2_]^•+^. Analysis of hyperfine couplings, in combination with density functional theory (DFT) calculations, establishes a close electronic analogy between [NHC─H_2_]^•+^ and the classical cyclohexadienyl radical [C_6_H_7_]^•^, highlighting *σ*–*π* hyperconjugation as the dominant stabilization motif. These findings identify imidazolium‐derived *σ*‐complex radicals as a unique class of carbene‐based open‐shell species and showcase imidazolium cations as noninnocent, redox‐active platforms for controlled radical generation under mild photochemical conditions.

## Graphical abstract

Visible‐light excitation of electron donor–acceptor (EDA) complexes between imidazolium salts and triarylamines enables controlled single‐electron transfer (SET) and reveals a family of carbene‐centered *σ*‐complex radical cations. Cryogenic electron paramagnetic resonance (EPR) captures the elusive [NHC─H]^•^ intermediate and its thermal evolution into persistent radicals.

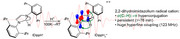

## 1 Introduction

**SCHEME 1:**
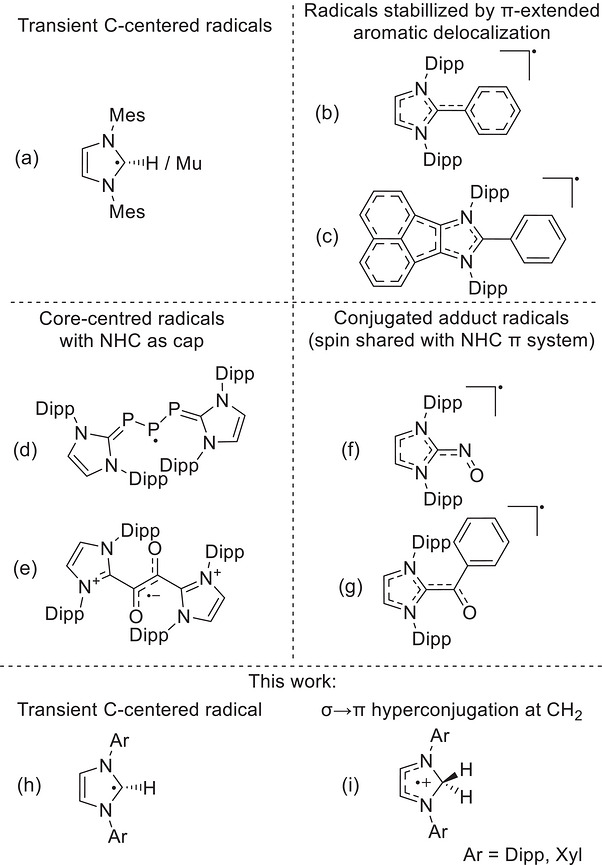
(a) Transient NHC and muonium adduct [NHC─Mu]^•^; (b–g) NHC‐derived radicals bearing a common IDipp framework that are stable or persistent at room‐temperature; (h, i) radical species investigated in this work.

*N*‐Heterocyclic carbenes (NHCs) are ubiquitous tools in synthesis and catalysis, serving as strongly *σ*‐donating and *π*‐accepting ligands as well as organocatalysts in a wide range of transformations [1, 2]. Access to free NHCs from their corresponding azolium salts is most commonly achieved by deprotonation at C2 using a Brønsted base [3]. Strong, nonnucleophilic bases such as KO*t*Bu, LiHMDS, or NaH exploit the relatively high acidity of the imidazolium C2─H bond (p*K*_a_ ≈ 21–24) to deliver imidazol‐2‐ylidenes cleanly under strictly anhydrous conditions [4, 5]. Despite the simplicity of this classical protocol, the fundamental mechanism by which such bases activate imidazolium salts has never been rigorously established. In particular, it remains unresolved whether reagents such as KO*t*Bu/LiHMDS operate exclusively as Brønsted bases or whether single‐electron pathways contribute, as both reagents are well documented to engage in single‐electron transfer (SET) chemistry in other contexts [6, 7, 8]. Compelling evidence that imidazolium salts are redox‐active was provided by Clyburne and co‐workers in 2004 [9]. They demonstrated that treatment of aryl‐substituted imidazolium salts with potassium metal in refluxing THF affords the corresponding NHCs, and that the same transformation can be achieved electrochemically in DMF. This redox reactivity was later exploited synthetically by Willans et al., enabling direct, base‐free access to NHC Cu(I)‐complexes via cathodic reduction of imidazolium salts [10]. Cyclic voltammetry revealed an irreversible cathodic event at −2.28 V versus SCE in DMF is assigned to one‐electron reduction of IMesH^+^ imidazolium cation (IMesH^+^ = 1,3‐dimesitylimidazolium) [9], followed by an anodic event at −0.20 V versus SCE corresponding to the oxidation of the free carbene. These data are fully consistent with the intermediacy of a neutral imidazolium‐derived radical, [NHC─H]^•^ (Scheme 1a), which undergoes subsequent hydrogen‐atom loss to generate the carbene. Direct spectroscopic validation of such species has remained elusive under preparative conditions; however, the muoniated analogue [NHC─Mu]^•^ (NHC = IMes and I*i*Pr_2_Me_2_) has been generated by *µ*‐beam irradiation of NHC solutions in THF and characterized by µSR spectroscopy, providing indirect support for the involvement of [NHC─H]^•^ in reductive carbene formation [11].

Beyond [NHC─H]^•^ radicals as transient intermediates, NHCs can support long‐lived and even isolable open‐shell species when the unpaired electron is sufficiently delocalized [12]. Such persistent NHC‐derived radicals span several distinct stabilization motifs that share a common practical element—strong kinetic protection by bulky *N*‐aryl substituents—yet differ fundamentally in where the unpaired electron is accommodated. One widely employed strategy is to export spin density into an extended aromatic *π*‐system. In C2‐arylated NHC radicals (Scheme 1b), the unpaired electron is stabilized by partial delocalization into the adjacent aryl substituent rather than being confined to the carbene ring itself [13]. This design principle was further developed in Mandal's *π*‐extended imidazole–aryl frameworks, which overcame earlier crystallization challenges for imidazole‐based carbon radicals through enhanced steric shielding and *π*‐stabilization [14]. Similar reasoning underpins Zhao's IPr(BIAN)‐derived radicals (Scheme 1C), where ring fusion is used to maximize spin delocalization and stabilize the open‐shell state [15]. In contrast, several persistent species employ NHCs primarily as spectator stabilizers, while the radical is centered elsewhere. In Tondreau's [(IDipp)_2_(μ‐P_3_)]^•^ (Scheme 1D), the unpaired electron resides predominantly on the pnictogen chain, with only minor spin density onto the flanking NHCs [16]. Similarly, Back's NHC‐supported triazenyl radicals place the dominant spin density on the N_3_ unit, with minimal involvement of the carbene carbons [17]. Kim's [IDipp─C(O)─C(O)─IDipp]^•+^ radical cations (Scheme 1E) represent an extreme version of this motif, in which the radical electron is largely localized on the dicarbonyl core rather than on the NHC framework [18]. A complementary stabilization strategy exploits the NHC as a *π*‐accepting sink that draws spin density across an exocyclic *π*‐bond. Park attributed the exceptional robustness of [NHC─NO]^•^ adducts (Scheme 1F) to the *π*‐accepting character of the carbene, which enables delocalization of spin density onto the carbene carbon following NO capture [19]. Similarly, Hudnall demonstrated that enhanced orbital overlap between the carbene and acyl *π*‐systems (Scheme 1G) provides a structural handle for stabilizing *α*‐acyl formamidinium radicals [20]. Collectively, these studies establish clear design principles for persistent NHC‐derived radicals: effective spin delocalization, combined with steric protection by bulky *N*‐aryl groups, enables long lifetimes and, in many cases, isolation and crystallographic characterization.

Despite these recent advances, structurally well‐defined radicals directly derived from imidazolium salts themselves, and stabilized without exocyclic *π*‐accepting substituents, remain exceedingly rare. In particular, the parent [NHC─H]^•^ radical continues to be spectroscopically under‐characterized. Here we show that controlled single‐electron reduction of imidazolium salts enables direct access to the neutral radicals [IDippH]^•^ and [IXylH]^•^ at cryogenic temperature and reveals their thermal evolution into the unusual and remarkably persistent 2,2‐dihydroimidazolium σ‐complex radical cations (Scheme 1H,I).

## 2 Results and Discussion

Our mechanistic objective was to directly observe the [NHC─H]^•^ radical postulated by Clyburne et al. [9] by controlled single‐electron reduction of imidazolium salts. For this, we employed photoinduced SET via excitation of the charge‐transfer (CT) band of ground‐state electron donor–acceptor (EDA) complexes [21, 22, 23, 24], a strategy that we and others have previously established for generating and characterizing fleeting radical species with high spectroscopic resolution [25, 26, 27, 28]. To maximize EDA complex formation, we selected the aryl‐substituted imidazolium cations [IXylH]^+^ and [IDippH]^+^ (IXylH^+^═1,3‐bis(2,6‐dimethylphenyl)imidazolium, IDippH^+^═1,3‐bis(2,6‐diisopropylphenyl)imidazolium), paired with electron‐rich triarylamine donors [29, 30, 31]. In aromatic donor–acceptor systems, association is dominated by dispersion and electrostatic contributions, with close face‐to‐face *π*–π stacking between extended aromatic surfaces providing the dominant stabilizing interaction [32, 33]. To ensure solubility in ethereal and aromatic solvents compatible with EDA chemistry, the parent chloride salts [IXylH]Cl and [IDippH]Cl were converted to their BArF_24_^−^ derivatives by salt metathesis with NaBArF_24_ [34] in methanol, affording [IXylH]BArF_24_ and [IDippH]BArF_24_ in 77% and 63% isolated yield, respectively, as analytically pure, pale‐yellow crystalline solids. Single‐crystal x‐ray diffraction confirmed the molecular structures of both salts (Figures S54 and S55) [35].

**FIGURE 1:**
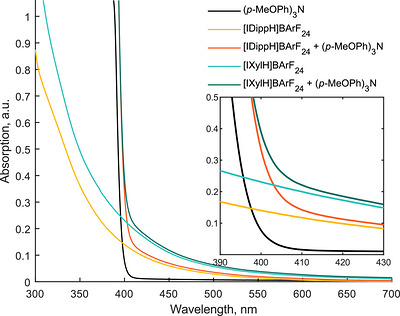
UV–vis absorption spectra of (*p*‐MeOPh)_3_N (black), [IDippH]BArF_24_ (yellow), and [IXylH]BArF_24_ (teal) in toluene (all 0.015 M), together with the corresponding equimolar mixtures of the imidazolium salt and donor (orange and dark green, respectively).

With these well‐defined acceptors in hand, we turned to evaluate the formation of EDA complexes with the electron‐rich donor (*p*‐MeOPh)_3_N. UV–vis spectra of equimolar mixtures of [IXylH]BArF_24_ or [IDippH]BArF_24_ with (*p*‐MeOPh)_3_N display an additional weak, but reproducible absorption feature in the 400–450 nm region (Figure 1), which is assigned to the CT transition of the ground‐state EDA complex [NHC─H^+^, (*p*‐MeOPh)_3_N]BArF_24_. Increasing the donor concentration to a 5:1 ratio results in a marked enhancement of this band (Figure S34), consistent with equilibrium formation of the donor–acceptor assembly. Time‐dependent DFT calculations on the [IDippH^+^, (*p*‐MeOPh)_3_N] encounter complex reproduce the experimentally observed spectral profile, predicting a modest increase in intensity superimposed on the intrinsic triarylamine absorption (Figure S36 and Tables S7 and S8). Analysis of the frontier molecular orbitals of the EDA complexes indicates that the charge‐transfer transition originates from the HOMO localized on the triarylamine donor to the LUMO centered on the imidazolium acceptor (Figure S37). Independent evidence for intimate donor–acceptor contact is provided by 1D NOESY spectroscopy of a 200 mM solution of the [IDippH]BArF_24_/(*p*‐MeOPh)_3_N mixture, which reveals intermolecular NOE interactions between the triarylamine protons and the imidazolium protons, confirming close spatial proximity of these two components in solution (Figure S39).

**SCHEME 2:**
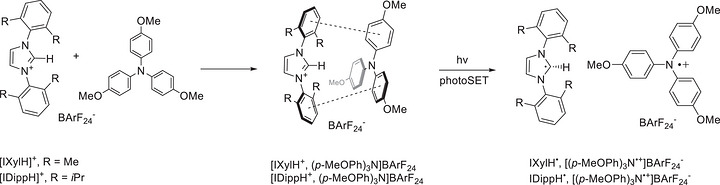
Formation of the amine–imidazolium EDA complex and subsequent photoinduced SET to generate the corresponding radical pair.

To rationalize the photophysical design, we performed a Mulliken‐type charge‐transfer analysis. The calculated at SCRF(toluene)/(U)*ω*B97X‐D/6‐311+G(d,p)//(U)*ω*B97X‐D/6‐31G(d) level of theory electron affinity of [IXylH]^+^ in toluene (−2.71 eV) and the ionization energy of (*p*‐MeOPh)_3_N (5.36 eV) place the lowest CT transition at approximately 2.6–2.7 eV (460–470 nm), in reasonable agreement with the experimentally observed CT band. The analogous values for [IDippH]^+^ (EA = −2.68 eV) predict nearly identical behavior. The full free‐energy profile for the [IXylH^+^, (*p*‐MeOPh)_3_N] system (Scheme 2) shows that formation of the closed‐shell EDA complex is mildly favorable (Δ*G* = −3.5 kcal·mol^−1^). Subsequent photoexcitation promotes SET to generate the open‐shell radical pair ^1^[IXylH]^•^[(*p*‐MeOPh)_3_N]^•+^ (Δ*G* = 72.1 kcal·mol^−1^), which can relax via intersystem crossing to the corresponding EPR‐active triplet ^3^[IXylH]^•^[(*p*‐MeOPh)_3_N]^•+^ (Δ*G* = 59.0 kcal·mol^−1^).

**FIGURE 2:**
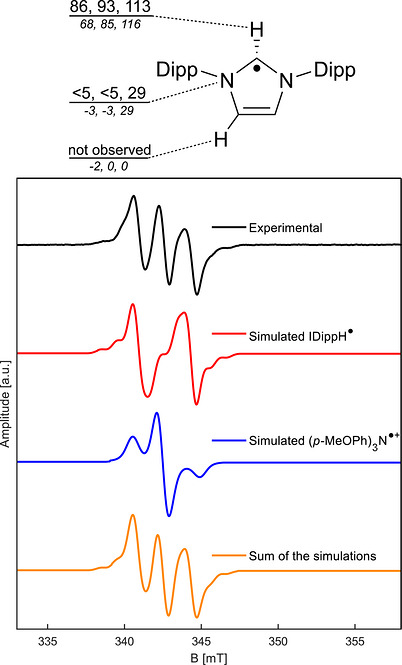
Top: characteristic hyperfine couplings (experimental and computed, *italic*, in MHz) of IDippH^•^. Bottom: Frozen‐matrix (100 K) X‐band EPR spectrum of an irradiated 2‐methyltetrahydrofuran solution of [IDippH]BArF_24_ (0.15 M) and (*p‐*MeOPh)_3_N (0.15 M), together with simulations showing the presence of [IDippH]^•^ and [(*p‐*MeOPh)_3_N]^•+^. Simulation parameters: [IDippH]^•^: *g* = [2.0035 2.0027 2.0035], *A* ^H^ = [86 93 113] MHz (C2‐*H*), 2×*A* ^N^ = 1 1 29 MHz, lwpp = 0.536195 and 0.104552 (Gaussian and Lorentzian), weight = 0.81. [(*p‐*MeOPh)_3_N]^•+^: *g* = [2.0046 2.0046 2.0025], *A* ^N^ = 1 1 61 MHz, lwpp = 0.574815 and 0.269137 (Gaussian and Lorentzian), weight = 1. Microwave frequency = 9.6054 GHz, microwave power = 0.3162 mW, modulation amplitude = 4 G.

We next sought direct spectroscopic evidence for the radical species generated upon CT‐band excitation under cryogenic conditions. Accordingly, 0.15 M solutions of [IDippH]BArF_24_ or [IXylH]BArF_24_ with equimolar (*p*‐MeOPh)_3_N in 2‐methyltetrahydrofuran [36] were frozen at 100 K directly in the resonator of the EPR spectrometer, which was equipped for in situ irradiation (Figure S40). Upon irradiation with 390 nm light, cw‐EPR signals immediately developed, which can be reproduced in both systems as the superposition of two *S* = 1/2 species (Figures 2 and S39): the 2‐imidazolyl radical ([IDippH]^•^ or [IXylH]^•^) and the triarylamine radical cation [(*p*‐MeOPh)_3_N]^•+^. No additional signals were observed, demonstrating clean and selective formation of the primary radical pair under these conditions, in contrast to earlier high‐energy (3 MeV) radiation chemistry experiments, where these species appeared only as minor components within complex radical mixtures [37, 38]. For the [IDippH]^•^ radical (Figure 2), the spectrum is described by an axial *g*‐tensor (*g*_⊥_ = 2.0035, *g*_∥_ = 2.0027) and pronounced hyperfine coupling to a single proton and two equivalent ^14^N nuclei. The strongly coupled proton exhibits a rhombic, nearly axial hyperfine tensor *A* ^H^ = [86, 93, 113] MHz, which translates to an isotropic hyperfine coupling of *A* ^H^_iso_ = 97 MHz. The two nitrogen atoms display an axial hyperfine tensor (*A* ^N^_⊥_ < 5 MHz, *A* ^N^_∥_ = 29 MHz), with principal axes rotated relative to the common *g* frame. This coupling pattern directly reflects a spin‐dense C2–H center with modest delocalization onto both imidazolium nitrogen atoms, fully consistent with the expected electronic structure of [IDippH]^•^. The accompanying radical is unambiguously assigned to [(*p*‐MeOPh)_3_N]^•+^, which exhibits an axial *g*‐tensor (*g*_⊥_ = 2.0046, *g*_∥_ = 2.0025) and an axial ^14^N hyperfine tensor (*A* ^N^_⊥_ = <5 MHz and *A* ^N^_∥_ = 61 MHz), in good agreement with reported isotropic values [31, 39]. Further support for the assignment of [IDippH]^•^ was obtained by examining the deuterated analogue [IDippD]^•^ generated from [IDippD]BArF_24_. As expected, replacing the C2‐bound proton by deuterium leads to a pronounced change in the hyperfine pattern, reflecting both the lower gyromagnetic ratio of deuterium (𝛾_p_/𝛾_D_ = 6.514) [40] and its different nuclear spin (*I*_H_ = ½, *I*_D_ = 1) (Figure S43). In an analogous experiment with [IXylH]BArF_24_, in situ irradiation in the presence of (*p*‐MeOPh)_3_N afforded the corresponding imidazolyl radical [IXylH]^•^, characterized by an axial *g*‐tensor (*g*_⊥_ = 2.0041, *g*_∥_ = 2.0031), a rhombic hyperfine coupling to the C2‐bound proton *A* ^H^ = [67, 88, 108] MHz, and an axial ^14^N coupling (*A* ^N^_⊥_ < 5 MHz, *A* ^N^_∥_ = 26 MHz); the [(*p*‐MeOPh)_3_N]^•+^ parameters remained unchanged (Figure S42).

Crucially, the experimentally observed proton hyperfine couplings closely match those of the muonium‐substituted analogue [IMes–Mu]^•^ reported by Clyburne et al. [11]. For [IMes─Mu]^•^, an isotropic hyperfine coupling to the muon of *A* ^µ^_iso_ = 287 MHz was measured. Using the muon‐to‐proton gyromagnetic ratio (𝛾_µ_/𝛾_p_ = 3.183) [41], this corresponds to a proton‐equivalent coupling of *A* ^H^_iso_ = 90 MHz, which is in excellent agreement with the values obtained here (*A* ^H^_iso_ = 97 MHz for [IDippH]^•^ and 88 MHz for [IXylH]^•^).

**FIGURE 3:**
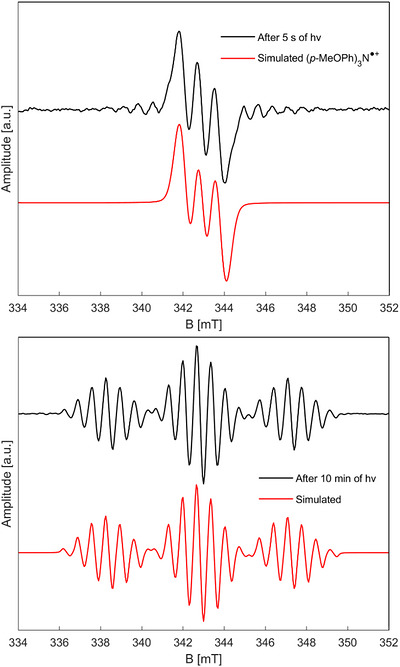
Top: room‐temperature X‐band EPR spectrum of an irradiated toluene solution of [IDippH]BArF_24_ (0.15 M) and (*p‐*MeOPh)_3_N (0.15 M), recorded 5 s after the onset of irradiation (390 nm), together with a simulation showing the presence of [(*p‐*MeOPh)_3_N]^•+^. Simulation parameters: *g*_iso_ = 2.0040, *A* ^N^_iso_ = 22.9 MHz, 6 × *A* ^H^_iso_ = 4.6 MHz (*o*‐Ph*H*), lwpp = 0.3303 and 0.0904 (Gaussian and Lorentzian). Microwave frequency = 9.6185 GHz; power = 3.162 mW; modulation amplitude = 4 G. Bottom: room‐temperature X‐band EPR spectrum of the same solution recorded 10 min after the onset of irradiation (390 nm), together with the corresponding simulation. Simulation parameters: *g*_iso_ = 2.00377, 2×*A* ^H^_iso_ = 123.5 MHz, 2×*A* ^N^_iso_ = 18.9 MHz, 2×*A* ^H^_iso_ = 20.3 MHz, lwpp = 0.1250 and 0.0832 (Gaussian and Lorentzian). Microwave frequency = 9.6185 GHz; power = 3.162 mW; modulation amplitude = 4 G.

**FIGURE 4:**
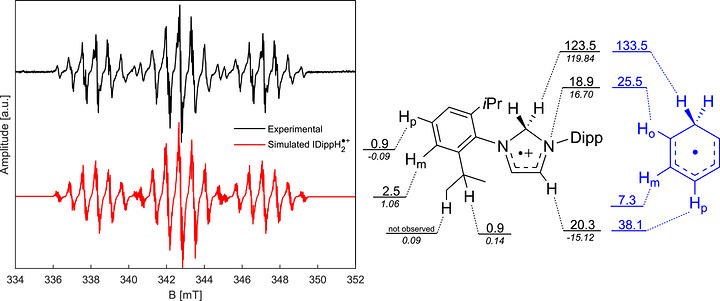
Left: room‐temperature X‐band EPR spectrum of an irradiated toluene solution of [IDippH]BArF_24_ (0.15 M) and (*p‐*MeOPh)_3_N (0.15 M), recorded with a reduced modulation amplitude (0.25 G instead of 4 G used in Figure 3), together with a simulation of [IDippH_2_]^•+^. Simulation parameters: *g*_iso_ = 2.00377, 2 × *A* ^H^_iso_ = 123.5 MHz (C2‐*H*), 2 × *A* ^N^_iso_ = 18.9 MHz, 2 × *A* ^H^_iso_ = 20.3 MHz (C4,5‐*H*), 2 × *A* ^H^_iso_ = 0.9 MHz (*p‐*Dipp*H*), 4 × *A* ^H^_iso_ = 2.5 MHz (*m*‐Dipp*H*), 4 × *A* ^H^_iso_ = 0.9 MHz, (C*H*(CH_3_)_2_), lwpp = 0.0158 and 0.0106 (Gaussian and Lorentzian). Microwave frequency = 9.6126 GHz; power = 3.162 mW, modulation amplitude = 0.25 G. Right: structural motif and characteristic hyperfine couplings (experimental and computed, *italic*, in MHz) of the 2,2‐dihydroimidazolium radical cation (black) and the cyclohexadienyl radical (blue) [42], highlighting the shared *σ*–*π* hyperconjugative stabilization.

While cryogenic photolysis traps the radical pair, increasing the temperature brings about a striking secondary transformation. When toluene solutions of [IDippH]BArF_24_/(*p*‐MeOPh)_3_N are irradiated at room temperature, the EPR spectrum recorded within the first seconds of illumination shows exclusively the triarylamine radical cation [(*p*‐MeOPh)_3_N]^•+^, as expected given the transient nature of [IDippH]^•^ (Figure 3, top). Over the course of several minutes, this signal decays and is replaced by a new, highly structured spectrum featuring a characteristic 1:2:1 triplet pattern with an exceptionally large hyperfine splitting of approximately 90 G (Figure 3, bottom). This species is remarkably persistent, with a measured lifetime of τ = 76 min at room temperature (Figure S45). The dominant triplet pattern indicates the presence of two equivalent, strongly coupled *I* = ½ nuclei (2 × *A*_iso_ = 123.5 MHz), accompanied by additional couplings to two *I* = 1 nuclei (2 × *A*_iso_ = 18.9 MHz) and two further *I* = ½ nuclei (2 × *A*_iso_ = 20.3 MHz). Based on the molecular structure, these interactions are assigned to the C2 methylene protons, the two nitrogen atoms of the imidazolium ring, and the two backbone protons, respectively. Using a reduced modulation amplitude, smaller hyperfine couplings attributable to the Dipp substituents could be resolved (2 × *A* ^H^_iso_ = 0.9 MHz, 4 × *A* ^H^_iso_ = 2.5 MHz, and 4 × *A* ^H^_iso_ = 0.9 MHz; Figure 4, left). This hyperfine pattern is fully consistent with assignment to the 2,2‐dihydroimidazolium *σ*‐complex radical cation [IDippH_2_]^•+^ (for details on [IXylH_2_]^•+^ see Figures S46).

**FIGURE 5:**
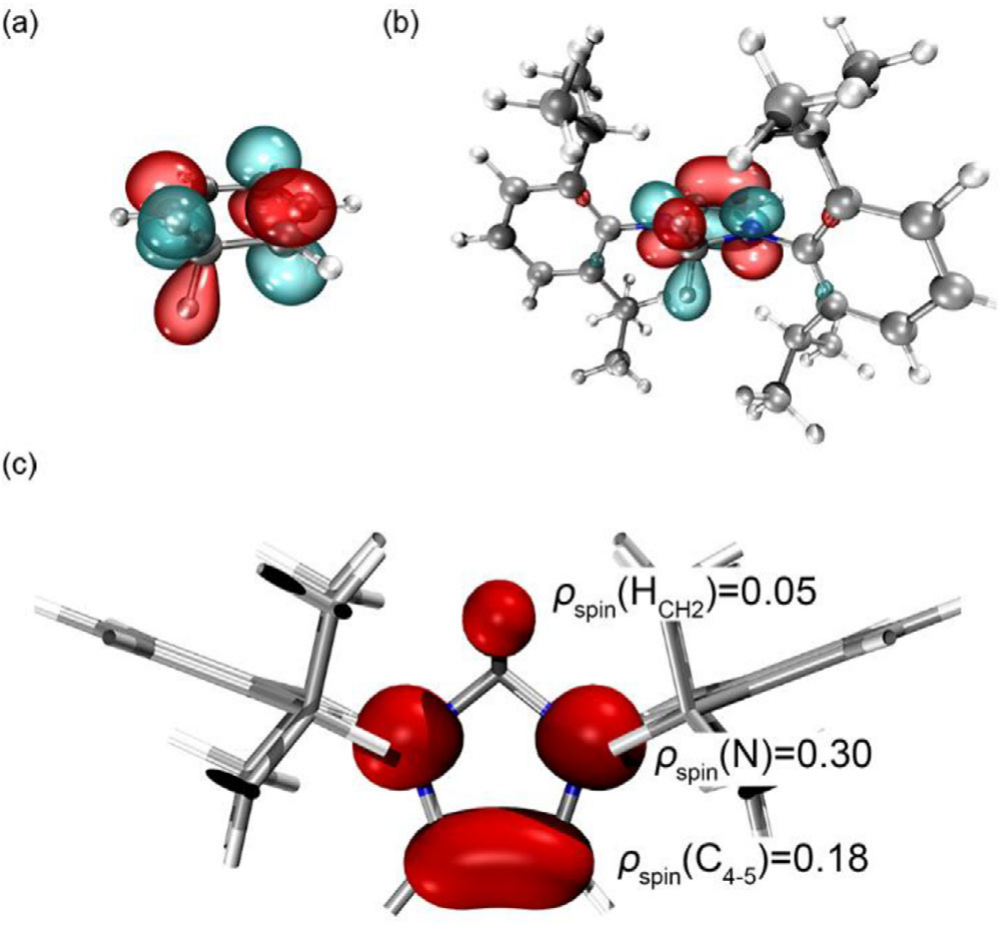
SOMO of (a) the cyclohexadienyl radical ([C_6_H_7_]^•^) and (b) the 1,3‐Dipp‐2,2‐dihydroimidazolium radical cation ([IDippH_2_]^•+^). (c) Mulliken spin density map of [IDippH_2_]^•+^.

The magnitude of the 2 × H hyperfine coupling immediately evokes the classical cyclohexadienyl radical ([C_6_H_7_]^•^), whose two methylene protons exhibit isotropic hyperfine coupling constants of approximately 134–135 MHz (≈48 G) [42, 43, 44, 45]. This characteristic feature is a manifestation of the Whiffen effect, in which symmetry‐enhanced σ(C─H)–π hyperconjugation in a cyclic *π*‐radical leads to anomalously large *β*‐proton couplings [46, 47]. In [IDippH_2_]^•+^, the nearly identical hyperfine coupling (123.5 MHz) indicates the same electronic motif: a localized CH_2_ fragment electronically integrated into a π‐delocalized ring (Figure 4, right) [37]. In this sense, [IDippH_2_]^•+^ is best described as an imidazolium‐embedded cyclohexadienyl radical analogue, in which *σ*–π hyperconjugation within the N─C─C─N unit stabilizes the open‐shell structure (Figure S56), thereby fundamentally distinguishing it from the persistent [NHC─R]^•^ radicals, where spin and charge are instead stabilized by exocyclic *π*‐accepting substituents. Spin‐density maps (Figure 5) and DFT‐calculated hyperfine couplings are in line with the experimentally found EPR parameters (Figure 4, right; Tables S2–S6), confirming that the unpaired electron is delocalized over the N─C─C─N core with pronounced *σ*–*π* hyperconjugation involving the methylene hydrogens.

**FIGURE 6:**
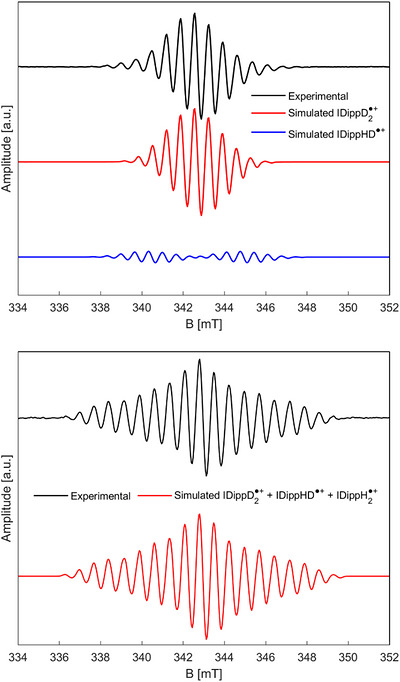
Top: room‐temperature X‐band EPR spectrum of an irradiated toluene solution of [IDippD]BArF_24_ (0.15 M) and (*p‐*MeOPh)_3_N (0.15 M), together with a simulation showing the presence of [IDippD_2_]^•+^ and [IDippHD]^•+^. Simulation parameters: [IDippD_2_]^•+^: *g*_iso_ = 2.0030, 2 × *A* ^D^_iso_ = 18.9 MHz (C2‐*D*), 2 × *A* ^N^_iso_ = 18.2 MHz, 2 × *A* ^H^_iso_ = 20.7 MHz (C4,5‐*H*), lwpp = 0.1185 and 0.0662 (Gaussian and Lorentzian); [IDippHD]^•+^: *g*_iso_ = 2.0030, *A* ^D^_iso_ = 18.8 MHz (C2‐*D*), *A* ^H^_iso_ = 123.5 MHz (C2‐H), 2 × *A* ^N^_iso_ = 18.4 MHz, 2 × *A* ^H^_iso_ = 20.1 MHz (C4,5‐*H*), lwpp = 0.0926 and 0.0921 (Gaussian and Lorentzian). Ratio [IDippD_2_]^•+^:[IDippHD]^•+^ = 1:0.19. Microwave frequency = 9.6079 GHz; power = 3.162 mW; modulation amplitude = 4 G. Bottom: room‐temperature X‐band EPR spectrum of an irradiated toluene solution of [IDippH]BArF_24_ (0.04 M), [IDippD]BArF_24_ (0.11 M) in a 1:3 ratio, and (*p‐*MeOPh)_3_N (0.15 M), together with a simulation showing the presence of [IDippD_2_]^•+^, [IDippHD]^•+^, and [IDippH_2_]^•+^ (1:1.1:1). For the details of the simulation and individual simulations, see Figure S50.

**SCHEME 3:**
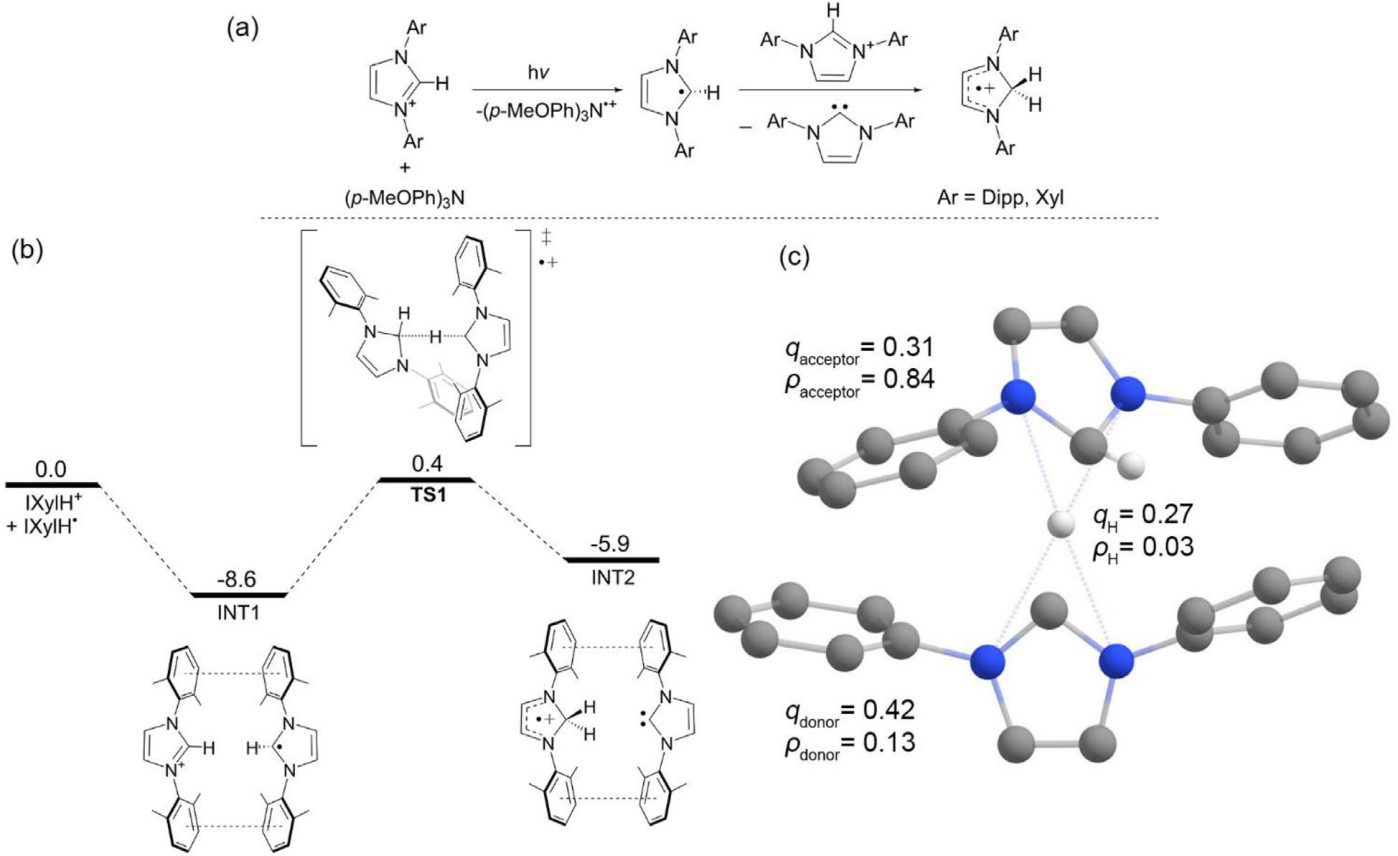
(a) Postulated mechanism of formation of the 2,2‐dihydroimidazolium radical cation. (b) Computed relative Gibbs free energies ΔG (kcal·mol^−1^) for the formation of [IXylH_2_]^•+^, obtained at the SCRF(Toluene)/(U)*ω*B97X‐D/6‐311+G(d,p) // (U)*ω*B97X‐D / 6–31G(d) level of theory. (c) Proton‐like hydrogen transfer transition state between the two imidazolium fragments and the respective natural charges *q*_NPA_ and natural spin densities ρ (U*ω*B97X‐D/6‐31G*). CH_3_ groups and most H atoms are omitted for clarity.

To determine the origin of the second methylene hydrogen atom of [IDippH_2_]^•+^, the C2 position of [IDippH]BArF_24_ was selectively deuterated by straightforward H/D exchange in CD_3_OD (quantitative yield; 4% residual [IDippH]BArF_24_ by ^1^H NMR [48], see Supporting Information). Under otherwise identical conditions, in situ irradiation of a toluene solution of [IDippD]BArF_24_ and (*p*‐MeOPh)_3_N results in collapse of the characteristic 1:2:1 triplet, producing a narrow spectrum dominated by [IDippD_2_]^•+^ with minor contributions from [IDippHD]^•+^ resulting from the residual IDippH^+^ (Figure 6, top); the same experiment with [IXylD]BArF_24_ afforded a similar spectrum (Figure S48). As expected, the hyperfine coupling of the C2‐bound deuterons is reduced by a factor of 6.5 relative to the corresponding protons, reflecting the proton‐to‐deuteron gyromagnetic ratio, while the change in nuclear spin alters the multiplet pattern. When protiated and deuterated salts are mixed in a 3:1 ratio, all isotopologues ([IDippH_2_]^•+^, [IDippHD]^•+^, [IDippD_2_]^•+^) are observed in the EPR spectrum (Figure 6, bottom), demonstrating H/D scrambling and establishing that formation of the *σ*‐complex radical cation proceeds via a bimolecular H/D transfer between the imidazolium salt and imidazolyl radical (Scheme 3a). Likewise, deuteration was used to confirm the assignment of the backbone hydrogens. Refluxing [IDippH]Cl in D_2_O / K_2_CO_3_ afforded [IDippD(2,4,5‐D_3_)]Cl [49], and subsequent aqueous anion exchange with NaBArF_24_ restored protium at C2 while retaining deuterium at C4/C5, giving [IDipp(4,5‐D_2_)H]BArF_24_ (see Supporting Information). In situ irradiation of a toluene solution of this salt with (*p*‐MeOPh)_3_N gave an EPR spectrum ([IDipp(4,5‐D_2_)H_2_]^•+^) with essentially the same spectral span and dominant CH_2_ hyperfine couplings as [IDippH_2_]^•+^, but a markedly simplified multiplet pattern, consistent with attenuation of the backbone proton couplings (Figure S49).

To further elucidate the mechanism of formation of [NHC─H_2_]^•+^, we performed DFT calculations at the SCRF(toluene)/(U)*ω*B97X‐D/6‐311+G(d,p)//(U)*ω*B97X‐D/6‐31G(d) level of theory for the computationally less demanding IXyl system (Scheme 3b). The calculations reveal favorable association of the neutral 2‐imidazolyl radical [IXylH]^•^ with an unreacted IXylH^+^ to form van de Waals complex [IXylH^+^, IXylH^•^] (INT1, Δ*G* = −8.6 kcal·mol^−1^), which is preorganized for facile proton transfer via a low barrier transition state (TS1, Δ*G* ^‡^ = 9.0 kcal·mol^−1^) affording [IXyl, IXylH_2_^•+^] (INT2, Δ*G* = −5.9 kcal·mol^−1^). Analysis of the electronic structure of the computed transition state establishes this step as a bimolecular open‐shell proton transfer, rather than hydrogen‐atom transfer. The migrating hydrogen is positively charged and spin‐poor (*q* = +0.23, *ρ*_s_ = 0.03), while most of the spin density remains localized on the accepting imidazolyl fragment (*ρ*_s_ = 0.84; Scheme 3c). Notably, this behavior is consistent with the 2‐imidazolyl radical being intrinsically more basic than the corresponding NHC.

Following *σ*‐complex formation, the liberated carbene is predicted to undergo rapid electron transfer with the available [(*p*‐MeOPh)_3_N]^•+^. This step is thermodynamically strongly favored based on the reported oxidation potentials of the related IMes (−0.55 V vs. Fc/Fc^+^ in THF) [9] and (*p‐*MeOPh)_3_N (+0.23 V vs. Fc/Fc^+^ in MeCN) [50]. Consistent with this prediction, independent mixing of chemically generated [(*p*‐MeOPh)_3_N]^•+^[PF_6_]^−^ with IDipp results in immediate bleaching of the characteristic blue color of the amine radical cation and almost complete loss of the EPR signal, indicating rapid decay of the transient [IDipp]^•+^ species [51] (Figure S53) back to IDippH^+^. Hence, while the formation of INT2 from INT1 is computed to be slightly endergonic, the NHC moiety in INT2 rapidly reacts with [(*p*‐MeOPh)_3_N]^•+^, thus providing additional driving force for the formation of [NHC─H_2_]^•+^. Although several carbenes are known to activate dihydrogen [52], [NHC─H_2_]^•+^ cannot arise from oxidative addition of H_2_ to the elusive NHC radical cation [53]. Instead, DFT calculations show that [IXyl]^•+^ reacts with H_2_ via facile hydrogen‐atom abstraction, directly yielding the closed‐shell imidazolium cation (Δ*G* ^‡^ = 1.4, Δ*G* = −24.1 kcal·mol^−1^; Figures S57 and S58).

The full energetic profile, therefore, accounts for both the selective accumulation and the remarkable persistence of the *σ*‐complex radical cations [NHC–H_2_]^•+^ on the EPR timescale. In parallel, ^1^H NMR monitoring the reaction before and after prolonged irradiation at 427 nm confirms that the imidazolium salt is effectively conserved, while the triarylamine donor undergoes progressive consumption (Figure S52), establishing (*p‐*MeOPh)_3_N as the sacrificial electron donor in the overall photoredox cycle. Importantly, although formation of free NHC is intrinsic to the reaction sequence, its steady‐state concentration remains negligible under the photoredox conditions due to rapid re‐oxidation by triarylamine radical cation, thereby preventing accumulation of persistent carbene in solution. [NHC─H_2_]^•+^ ultimately regenerates NHC─H^+^ by loss of H^•^, in direct analogy to the classical rearomatization of the cyclohexadienyl radical to benzene.

## 3 Conclusion

This work identifies imidazolium‐derived, *σ*‐delocalized 2,2‐dihydroimidazolium radical cations ([IDippH_2_]^•+^ and [IXylH_2_]^•+^) as a distinct and structurally well‐defined class of carbene‐based radicals accessible under mild photochemical conditions. Irradiation of the CT bands of imidazolium/triarylamine EDA complexes enables direct spectroscopic observation of the neutral 2‐imidazolyl ([NHC─H]^•^) intermediate and reveals its controlled thermal evolution to persistent σ‐complex radical cations. EPR spectroscopy combined with DFT analysis establishes these species as imidazolium‐embedded analogues of the cyclohexadienyl radical, stabilized by pronounced *σ*–*π* hyperconjugation within the N─C─C─N framework. By positioning these radicals on the continuum between transient [NHC–H]^•^ adducts and classical [NHC–R]^•^ systems, this study clarifies fundamental electronic factors governing carbene‐centered radical stability and highlights imidazolium salts as noninnocent redox platforms for rational access to well‐defined open‐shell chemistry.

## Author Contributions

**Filipp M. Kolomeychuk**: investigation, writing ‐ original draft, writing ‐ review and editing, data curation, methodology, validation, visualization. **Lars J. C. van der Zee**: conceptualization, investigation, methodology, writing ‐ review and editing. **Simon Mathew**: data curation, writing ‐ review and editing, methodology. **Bas de Bruin**: methodology, writing ‐ review and editing. **J. Chris Slootweg**: conceptualization, funding acquisition, writing ‐ review and editing, project administration, supervision, resources.

## Conflicts of Interest

The authors declare no conflicts of interest.

## Supporting information



The data that support the findings of this study are available in the Supporting Information of this article. The authors have cited additional references within the SI [49, 52, 54, 55, 56, 57, 58, 59, 60, 61, 62, 63, 64, 65, 66, 67, 68, 69, 70, 71].**Supporting File 1**: anie72383‐sup‐0001‐SuppMat.docx.

**Supporting File 2**: anie72383‐sup‐0002‐Data.zip.

## Data Availability

The data that support the findings of this study are available in the Supporting Information of this article.
