# Supplementary material for: From [NHC─H]• to Persistent σ‐Complex Radicals: Photoinduced Radical Chemistry of Imidazolium Salts
Source: Angew Chem Int Ed Engl. 2026 Apr 29;65(24):e5517752. doi: 10.1002/anie.5517752 (PMC13245608; doi:10.1002/anie.5517752)
Supplement: Supplementary file 1 — The data that support the findings of this study are available in the Supporting Information of this article. The authors have cited additional references within the SI [49, 52, 54, 55, 56, 57, 58, 59, 60, 61, 62, 63, 64, 65, 66, 67, 68, 69, 70, 71].Supporting File 1: anie72383‐sup‐0001‐SuppMat.docx. [file ANIE-65-e5517752-s002.docx]

Supporting Information

**From [NHC–H]^•^ to Persistent σ-Complex Radicals: Photoinduced Radical Chemistry of Imidazolium Salts**

Filipp M. Kolomeychuk, Lars J. C. van der Zee, Simon Mathew, Bas de Bruin, and J. Chris Slootweg*

Table of Contents

[I. Experimental Section 3](#_Toc223955393)

[Synthesis of [IXylH]BArF_24_ (1,3-Bis(2,6-dimethylphenyl)imidazolium tetrakis[3,5-bis(trifluoromethyl)phenyl]borate). 3](#_Toc223955394)

[Synthesis of [IDippH]BArF_24_ (1,3-Bis(2,6-diisopropylphenyl)imidazolium tetrakis[3,5-bis(trifluoromethyl)phenyl]borate). 5](#_Toc223955395)

[Synthesis of [IMes]BArF_24_ (1,3-dimesitylimidazolium tetrakis[3,5-bis(trifluoromethyl)phenyl]borate). 8](#_Toc223955396)

[Synthesis of [IXylD]BArF_24_ (2-Deuterio-1,3-bis(2,6-dimethylphenyl)imidazolium tetrakis[3,5-bis(trifluoromethyl)phenyl]borate). 9](#_Toc223955397)

[Synthesis of [IDippD]BArF_24_ (2-Deuterio-1,3-Bis(2,6-diisopropylphenyl)imidazolium tetrakis[3,5-bis(trifluoromethyl)phenyl]borate). 12](#_Toc223955398)

[Synthesis of [IDippD(2,4,5-D_3_)]Cl (2,4,5-Trideuterio-1,3-Bis(2,6-diisopropylphenyl)imidazolium chloride). 14](#_Toc223955399)

[Synthesis of [IDipp(4,5-D_2_)H]BArF_24_ (4,5-Dideuterio-1,3-Bis(2,6-diisopropylphenyl)imidazolium tetrakis[3,5-bis(trifluoromethyl)phenyl]borate). 15](#_Toc223955400)

[Synthesis of tris(4-anisyl)amine ((*p*-MeOPh)_3_N). 18](#_Toc223955401)

[II. Additional figures 19](#_Toc223955402)

[III. Single-crystal X-ray Diffraction 27](#_Toc223955403)

[IV. DFT calculations 30](#_Toc223955404)

[General information 30](#_Toc223955405)

[Transition state analysis 30](#_Toc223955406)

[Calculated and experimental EPR parameters 32](#_Toc223955407)

[TD-DFT Analysis of Excited States 34](#_Toc223955408)

[Coordinates of optimized structures 36](#_Toc223955409)

[V. References 46](#_Toc223955410)

# I. Experimental Section

**General Procedures**. The preparation of the EPR samples was carried out under an atmosphere of nitrogen inside a glovebox. Toluene and 2-methyltetrahydrofuran were distilled from potassium and stored over a sodium mirror. Dichloromethane-*d*_2_ (CD_2_Cl_2_) and chloroform-*d*_1_ (CDCl_3_) were distilled from P_2_O_5_, degassed and stored over activated molecular sieves (4 Å). IXyl·HCl, IDipp·HCl, IMes·HCl, NaBArF_24_, K_2_CO_3_, D_2_O, CD_3_OD and C_6_D_6_ were obtained from commercial sources and used as received. NMR spectra (δ in ppm) were recorded using a Bruker Avance AMX 400 or Bruker Avance Neo 300 spectrometer and were referenced to internal CDHCl_2_ (^1^H δ = 5.32, ^13^C δ = 53.84) or external BF_3_(OEt_2_) (^11^B), CFCl_3_ (^19^F) and 85% H_3_PO_4_ (^31^P). The EPR spectra were recorded on a Bruker EMXnano equipped with a variable temperature control and further analyzed and simulated using EasySpin 6.0.6^[1]^ and cwEPR 3.6.0^[2]^. Kessil lights PR160L-390 (390 nm, 55 W) and PR160L- 427 (427 nm, 45 W) and were used as light source. IR spectra were recorded on a Bruker Alpha-P spectrometer equipped with a single-reflection ATR sampling module. UV–vis spectra were recorded on a Shimadzu UV 2700 spectrophotometer. Mass spectra were measured on a AccuTOF GC v4g, JMS-T100GCV Mass spectrometer (JEOL, Japan). Melting points were measured in glass capillaries using a Büchi M-565 melting point apparatus and are uncorrected.

## Synthesis of [IXylH]BArF_24_ (1,3-Bis(2,6-dimethylphenyl)imidazolium tetrakis[3,5-bis(trifluoromethyl)phenyl]borate).

To a methanolic solution (5 mL) of NaBArF_24_ (425 mg, 0.5 mmol), a solution of IXyl·HCl (150 mg, 0.5 mmol) in methanol (5 mL) was added. The mixture was stirred at room temperature for 24 h. Subsequent addition of water (5 mL) induced precipitation, and a pale-yellow solid product was collected by filtration. Single crystals suitable for X-ray diffraction were obtained by vapor diffusion of *n*-pentane into a saturated *o*-difluorobenzene solution. Yield: 0.421 mg (369 mmol, 77%). m.p. = 160.0–162.2 °C. ^1^H NMR (300 MHz, CDCl_3_, 300 K): *δ* 8.17 (t, ^4^*J*(H,H) = 1.6 Hz, 1H, C2-*H*), 7.68 (br s, 8H, *o*-BArF_24_*H*), 7.49 (br s, 4H, *p*-BArF_24_*H*), 7.45 (d, ^4^*J*(H,H) = 1.6 Hz, 2H, C4,5–*H*), 7.42 (t, ^3^*J*(H,H) = 7.4 Hz, 2H, *p*-Xyl*H*), 7.24 (d, ^3^*J*(H,H) = 7.7 Hz, 4H, *m*-IXyl*H*), 2.08 (s, 12H, C*H*_3_). ^11^B NMR (96 MHz, CDCl_3_) δ −6.6. ^13^C NMR (75 MHz, CDCl_3_) δ 161.8 (q, ^1^*J*(C,B) = 49.7 Hz, *ipso*-BArF_24_^-^), 135.2 (s, *C*2), 134.9 (s, *o*-BArF_24_^-^), 134.0 (s, *o*-Xyl), 132.5 (s, *p*-Xyl), 132.0 (s, *ipso*-Xyl), 130.0 (s, *m*-Xyl), 129.0 (br q, ^2^*J*(C,F) = 31.8 Hz, *m*-BArF_24_^-^), 125.0 (s, *C*4,5), 124.7 (q, ^1^*J*(C,F) = 272.7 Hz, *C*F_3_), 117.6 (s, *p*-BArF_24_^-^), 17.2 (s, *C*H_3_). ^19^F NMR (282 MHz, CDCl_3_) δ −62.4 (s, 24F; C*F*_3_). HR-MS (ESI): calcd for C_19_H_21_N_2_ [M-BArF_24_^-^]^+^: 277.1705, found: 277.1694. IR (neat, cm^-1^): *ν* 3164, 1609, 1543, 1473, 1354, 1281, 1208, 1157, 1109, 949, 933, 885, 835, 773, 755, 744, 667, 712, 681, 577, 567, 447.


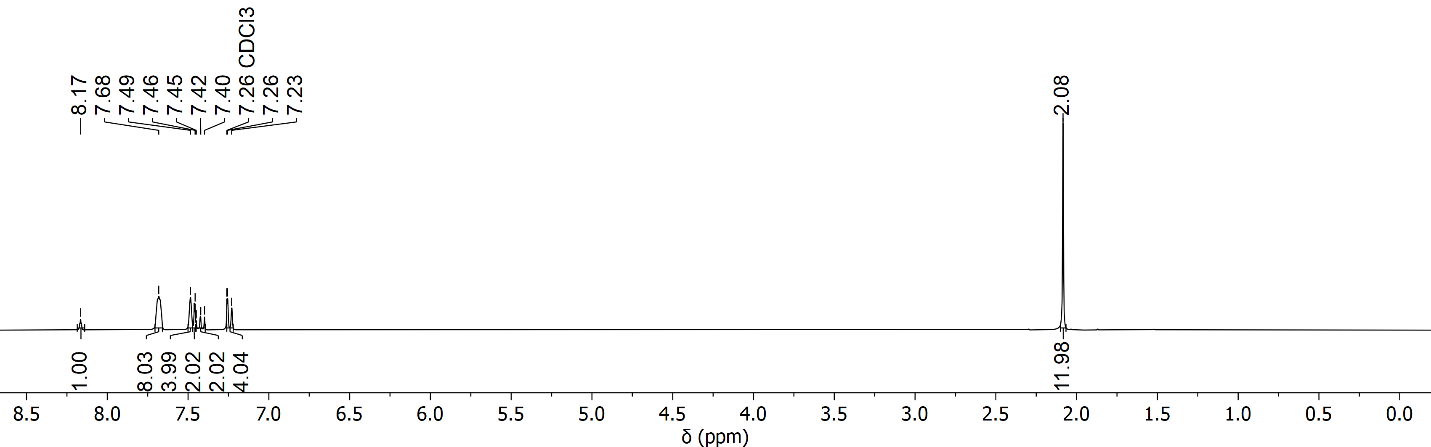


**Figure S1.** ^1^H NMR spectrum (300 MHz, CDCl_3_) of [IXylH]BArF_24_.


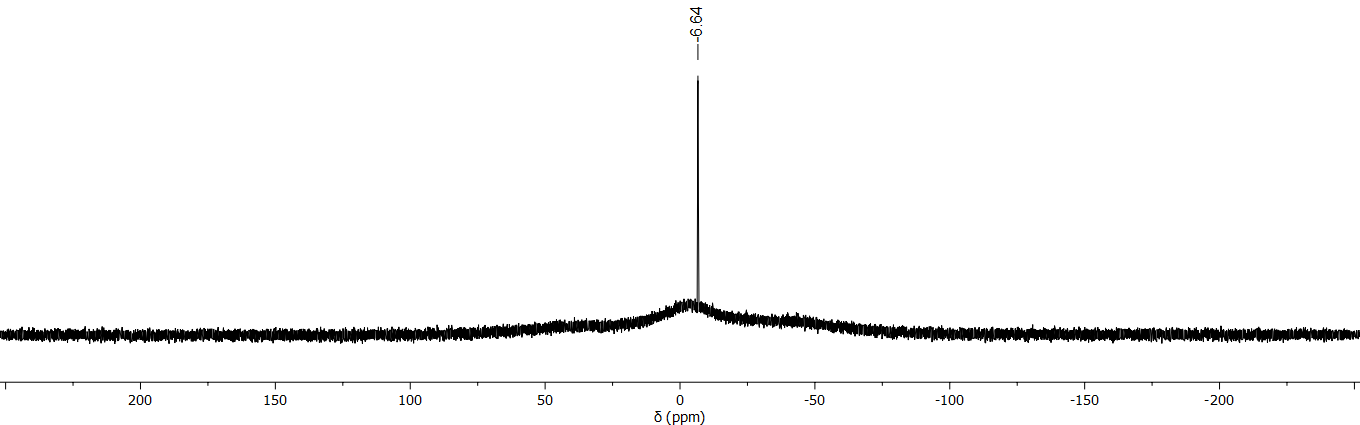


**Figure S2.** ^11^B NMR spectrum (96 MHz, CDCl_3_) of [IXylH]BArF_24_.


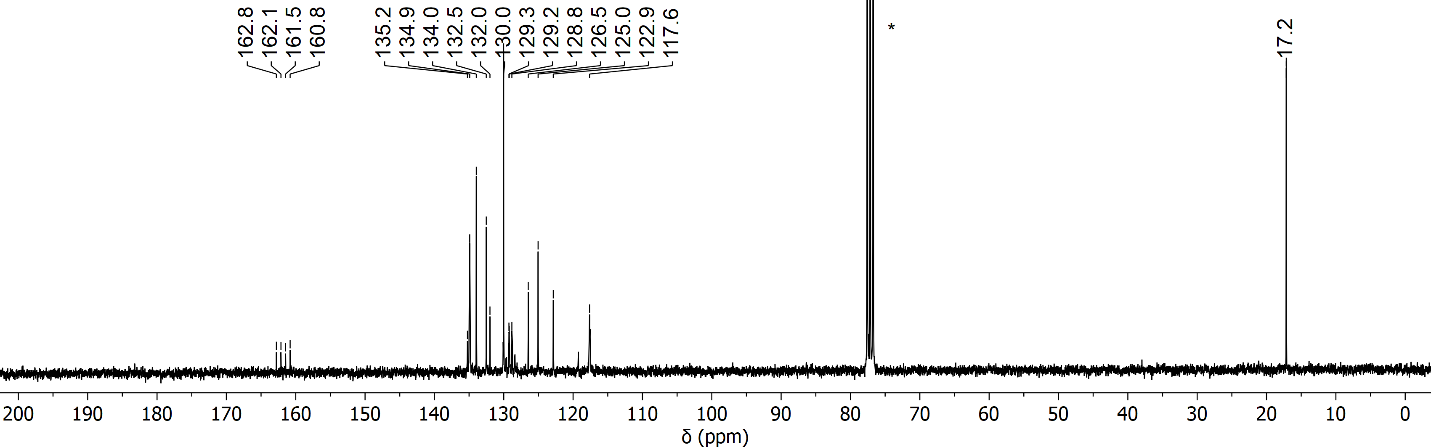


**Figure S3.** ^13^C NMR spectrum (75 MHz, CDCl_3_) of [IXylH]BArF_24_. *CDCl_3_.3


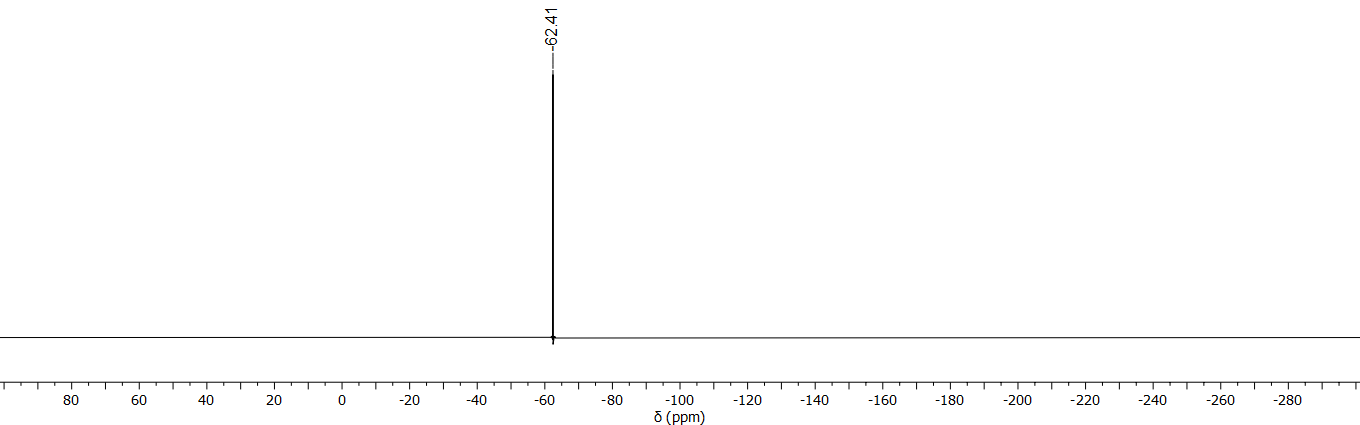


**Figure S4.** ^19^F NMR spectrum (282 MHz, CDCl_3_) of [IXylH]BArF_24_.


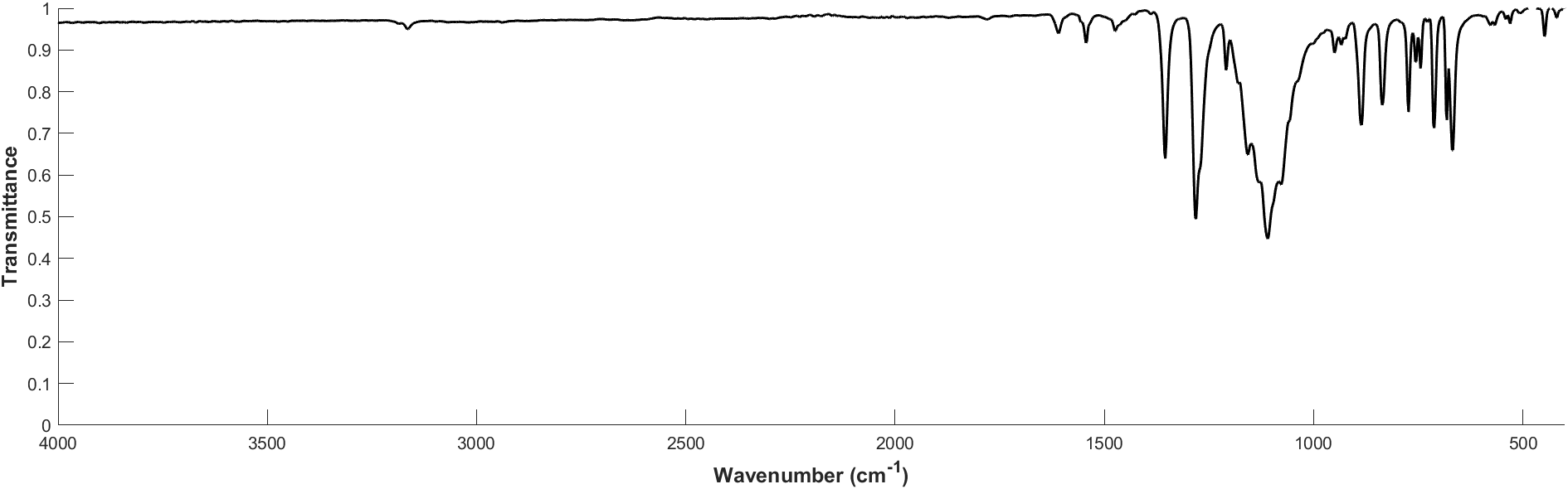


**Figure S5.** IR (neat) spectrum of [IXylH]BArF_24_.

## Synthesis of [IDippH]BArF_24_ (1,3-Bis(2,6-diisopropylphenyl)imidazolium tetrakis[3,5-bis(trifluoromethyl)phenyl]borate).

To a methanolic solution (7 mL) of NaBArF_24_ (798 mg, 0.5 mmol), a solution of IDipp·HCl (383 mg, 0.5 mmol) in methanol (7 mL) was added. The mixture was stirred at room temperature for 24 h. Subsequent addition of water (10 mL) induced precipitation, and a pale-yellow solid product was collected by filtration. Single crystals suitable for X-ray diffraction were obtained by layering a saturated toluene solution with *n*-pentane. Yield: 707 mg (564 mmol, 63%). m.p. = 160.4–162.5 °C. ^1^H NMR (300 MHz, CDCl_3_, 300K) δ 8.19 (t, ^4^*J* (H,H) = 1.6 Hz, 1H, C2-*H*), 7.67 (br s, 8H, *o*-BArF_24_*H*), 7.62 (t, ^3^*J* = 7.9 Hz, 2H, *p*-Dipp*H*), 7.52 (d, ^4^J(H,H) = 1.6 Hz, 2H, C4,5-*H*), 7.49 (br s, 4H, *p*-BArF_24_*H*), 7.38 (d, ^3^*J*(H,H) = 7.9 Hz, 4H, *m*-Dipp*H*), 2.30 (hept, ^3^*J*(H,H) = 7.0 Hz, 4H, C*H*(CH_3_)_2_), 1.24 (d, ^3^*J* = 6.8 Hz, 12H, CH(C*H*_3_)_2_), 1.17 (d, ^3^*J* = 6.9 Hz, 12H, CH(C*H*_3_)_2_). ^11^B NMR (96 MHz, CDCl_3_) δ −6.6. ^13^C NMR (75 MHz, CDCl_3_) δ 161.8 (q, ^1^*J*(C,B) = 49.9 Hz, *ipso*-BArF_24_^-^), 144.9 (s, *o*-Dipp), 136.5 (s, *C*2), 134.9 (s, *o*-BArF_24_^-^), 133.4 (s, *p*-Dipp), 129.0 (br q, ^2^*J*(C,F) = 31.3 Hz, *m*-BArF_24_^-^), 128.8 (s, *ipso*-Dipp), 125.9 (s, *C*4,5), 125.5 (s, *m*-Dipp), 124.7 (q, ^1^*J*(C,F) = 272.7 Hz, *C*F_3_), 117.6 (s, *p*-BArF_24_^-^), 29.4 (s, *C*H(CH_3_)_2_), 24.6 (s, CH(*C*H_3_)_2_), 23.7 (s, CH(*C*H_3_)_2_). ^19^F NMR (282 MHz, CDCl_3_) δ −62.4 (s, 24F; C*F*_3_). HR-MS (ESI): calcd for C_27_H_37_N_2_ [M-BArF_24_^-^]^+^: 389.2957, found: 389.2962. IR (neat, cm^-1^): *ν* 3148, 2971, 1610, 1540, 1466, 1352, 1271, 1161, 1118, 1059, 950, 929, 886, 837, 805, 757, 743, 713, 680, 668, 579, 446.


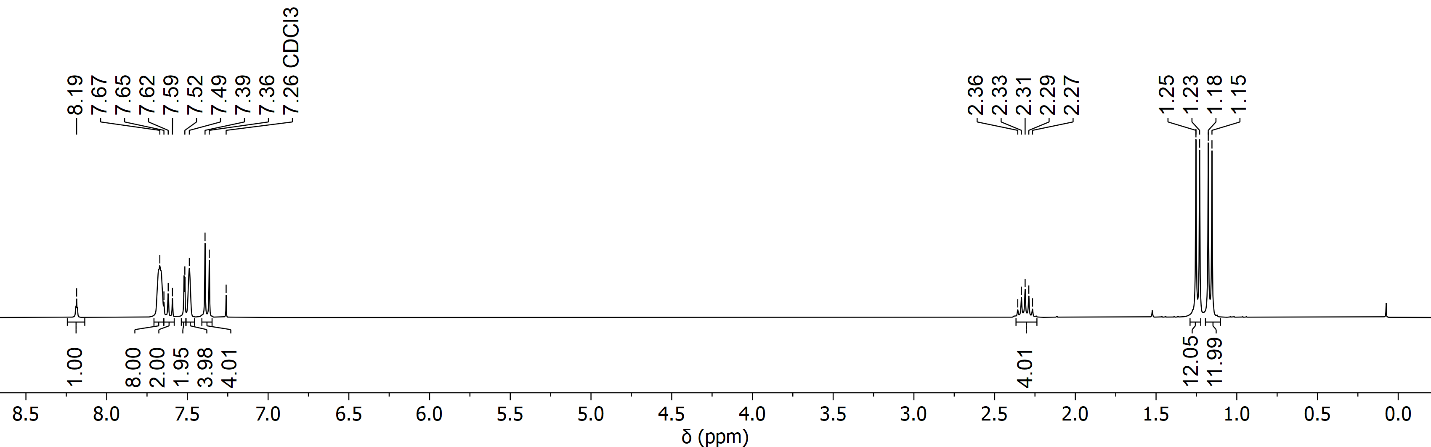


**Figure S6.** ^1^H NMR spectrum (300 MHz, CDCl_3_) of [IDippH]BArF_24_.


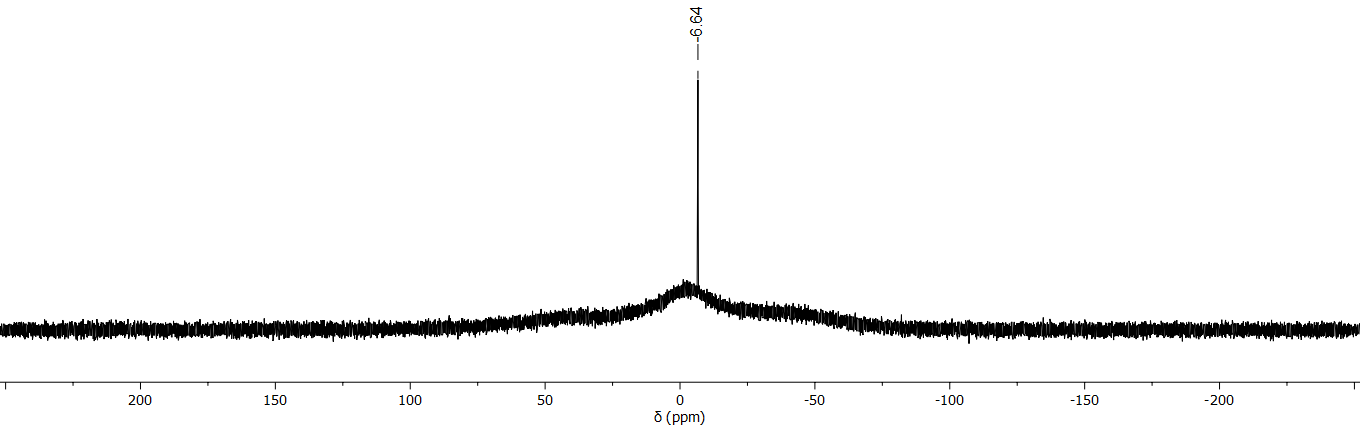


**Figure S7.** ^11^B NMR spectrum (96 MHz, CDCl_3_) of [IDippH]BArF_24_.


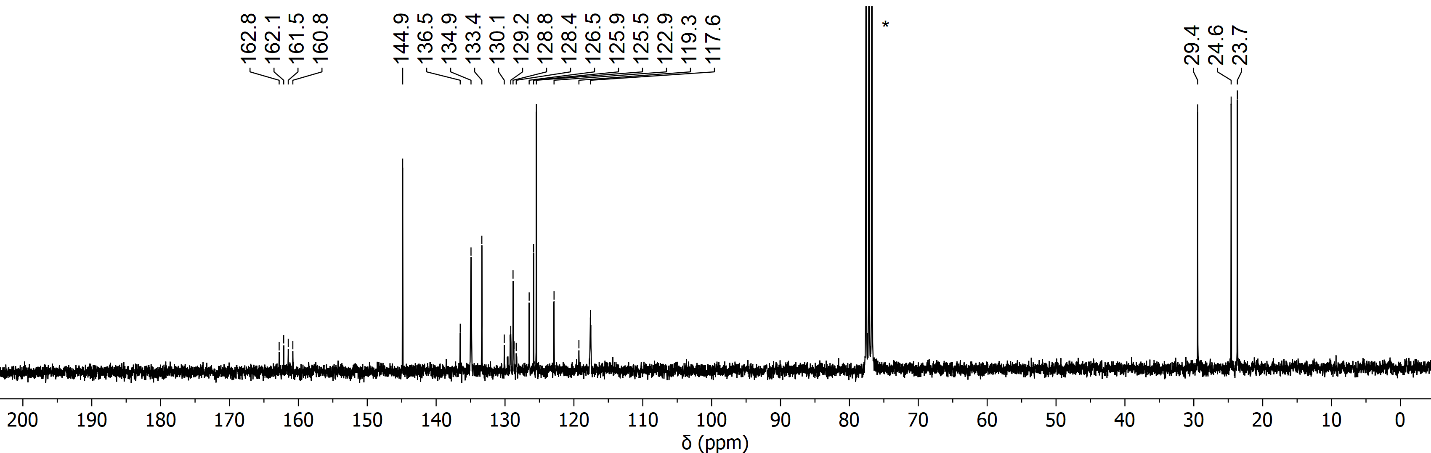


**Figure S8.** ^13^C NMR spectrum (75 MHz, CDCl_3_) of [IDippH]BArF_24_. *CDCl_3_.


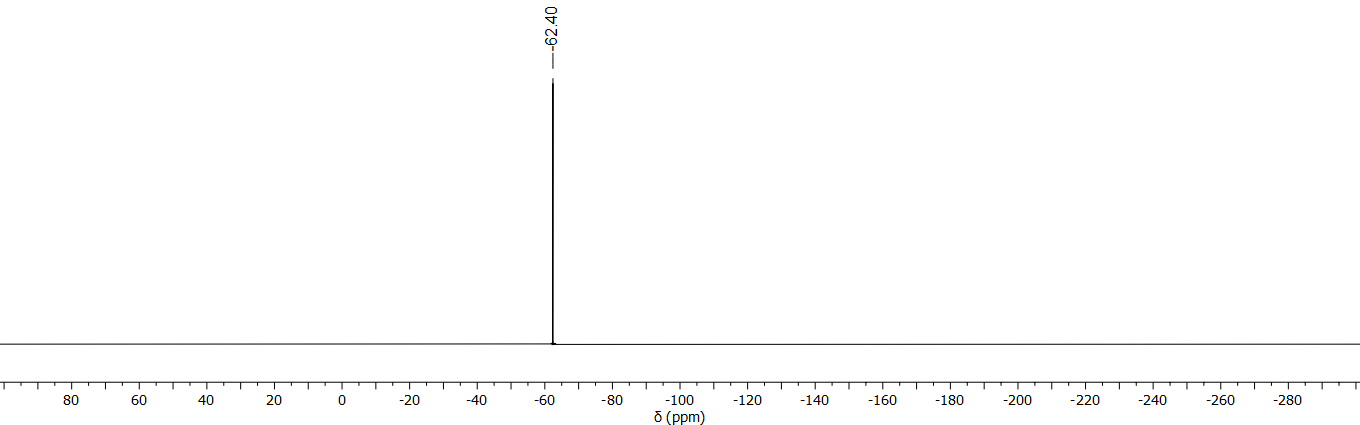


**Figure S9.** ^19^F NMR spectrum (282 MHz, CDCl_3_) of [IDippH]BArF_24_.


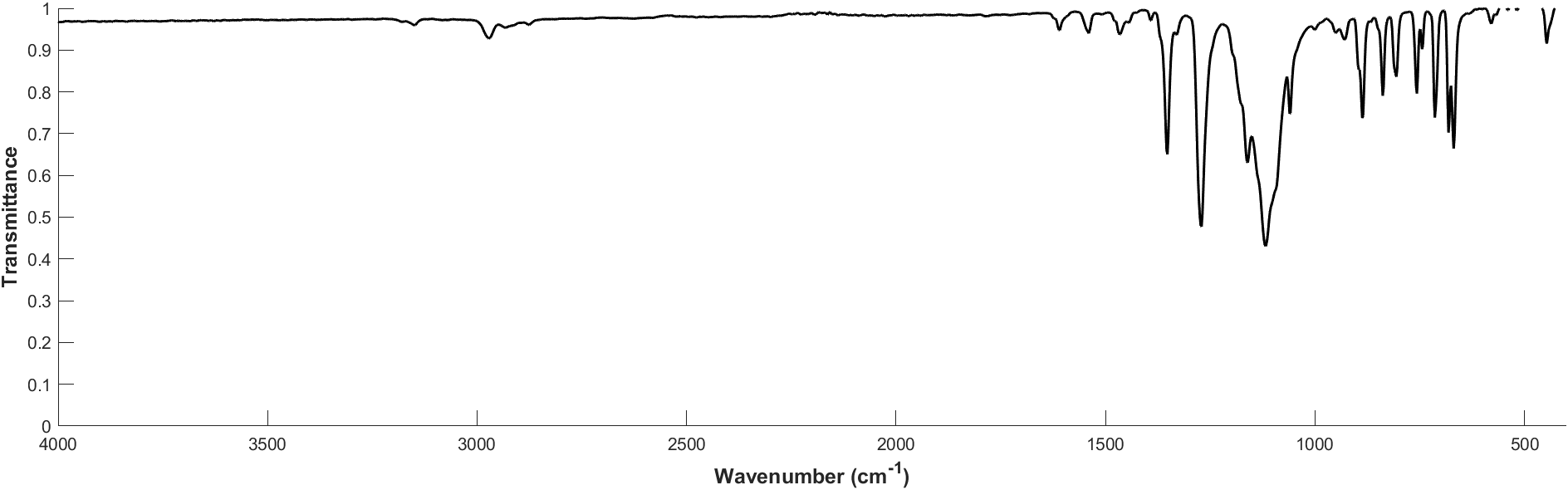


**Figure S10.** IR (neat) spectrum of [IDippH]BArF_24_.

## Synthesis of [IMes]BArF_24_ (1,3-dimesitylimidazolium tetrakis[3,5-bis(trifluoromethyl)phenyl]borate).

To a methanolic solution (7 mL) of NaBArF_24_ (520 mg, 0.59 mmol), a solution of IMes·HCl (200 mg, 0.59 mmol) in methanol (7 mL) was added. The mixture was stirred at room temperature for 24 h. Subsequent addition of water (10 mL) induced precipitation. The suspension was filtered to afford [IMesH]BArF_24_ as an off-white, viscous, tacky goo that was difficult to handle and did not form a free-flowing powder. The material was used only for preliminary characterization and was not pursued further in subsequent studies due to its poor physical handling properties. Yield: 0.421 mg (0.36 mmol, 61%). ^1^H NMR (300 MHz, CDCl_3_, 300K) δ 8.31 (br s, 1H, C2-*H*), 7.73 (br s, 8H, *o*-BArF_24_*H*), 7.56 (br s, 4H, *p*-BArF_24_*H*), 7.54 (br. s, 2H, C4,5-*H*), 7.13 (s, 4H, *m*-Mes*H*), 2.37 (s, 6H, *p*-C*H*_3_), 2.11 (s, 12H, *o*-C*H*_3_). ^13^C NMR (75 MHz, CD_2_Cl_2_) δ = 162.18 (q, ^1^*J*(C,B) = 49.8 Hz, *ipso*-BArF_24_^-^), 143.21 (s, *p*-Mes), 136.1 (s, *C*2), 135.3 (s, *o*-Dipp), 134.3 (s, *o*-Mes), 130.7 (s, *m*-Mes), 130.2 (s, *ipso-*Mes), 129.3 (br q, ^2^*J*(C,F) = 31.6 Hz, *m*-BArF_24_^-^), 125.6 (s, *C*4,5), 125.0 (q, ^1^*J*(C,F) = 272.4 Hz, *C*F_3_), 117.9 (s, *p*-BArF_24_^-^), 21.24 (s, *p*-*C*H_3_), 17.43 (s, *o*-*C*H_3_).


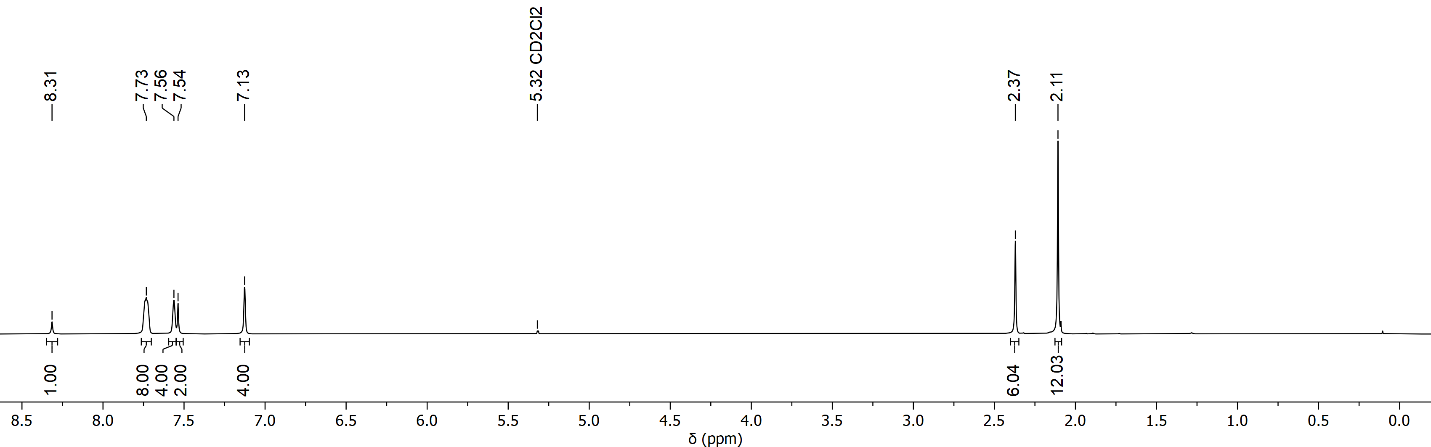


**Figure S11.** ^1^H NMR spectrum (300 MHz, CD_2_Cl_2_) of [IMesH]BArF_24_.


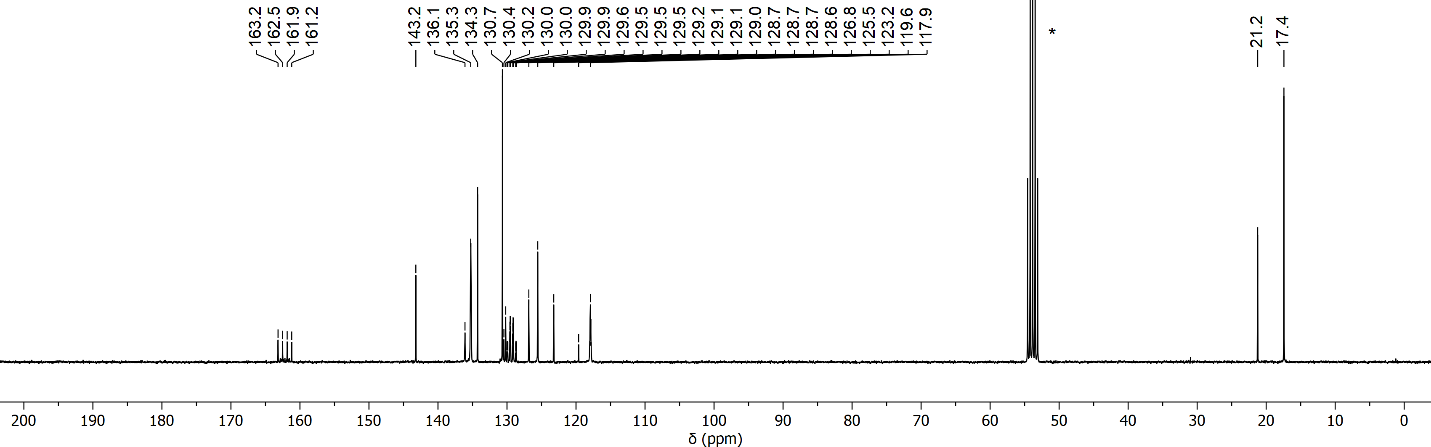


**Figure S12.** ^13^C NMR spectrum (75 MHz, CD_2_Cl_2_) of [IMesH]BArF_24_. *CD_2_Cl_2_.

## Synthesis of [IXylD]BArF_24_ (2-Deuterio-1,3-bis(2,6-dimethylphenyl)imidazolium tetrakis[3,5-bis(trifluoromethyl)phenyl]borate).

[IXylH]BArF_24_ (217 mg, 0.19 mmol) was dissolved in methanol-*d*_4_ (2 mL) in a Schlenk flask. The vessel was sealed, and the solution was stirred overnight at 50 °C. Evaporation of the solvent afforded the product as a pale-yellow powder. Yield: 217 mg (0.19 mmol, 100%). m.p. = 160.0–162.0 °C. ^1^H NMR (300 MHz, CD_2_Cl_2_, 300 K): *δ* 8.31 (t, 0.02H, residual C2-*H*), 7.70 (br s, 8H, *o*-BArF_24_*H*), 7.60 (s, 2H, C4,5–*H*), 7.55 (br s, 4H, *p*-BArF_24_*H*), 7.49 (t, ^3^*J*(H,H) = 7.4 Hz, 2H, *p*-Xyl*H*), 7.33 (d, ^3^*J*(H,H) = 7.7 Hz, 4H, *m*-IXyl*H*), 2.17 (s, 12H, C*H*_3_). ^2^H NMR (61 MHz, CH_2_Cl_2_) δ 8.35 (s, 1D, C2-*D*). ^11^B NMR (96 MHz, CDCl_3_) δ −6.6. ^13^C NMR (75 MHz, CDCl_3_) δ 161.8 (q, ^1^*J*(C,B) = 49.7 Hz, *ipso*-BArF_24_^-^), 134.9 (s, *o*-BArF_24_^-^), 134.0 (s, *o*-Xyl), 132.5 (s, *p*-Xyl), 132.0 (s, *ipso*-Xyl), 130.0 (s, *m*-Xyl), 129.0 (br q, ^2^*J*(C,F) = 31.8 Hz, *m*-BArF_24_^-^), 125.0 (s, *C*4,5), 124.7 (q, ^1^*J*(C,F) = 272.7 Hz, *C*F_3_), 117.6 (s, *p*-BArF_24_^-^), 17.1 (s, *C*H_3_). ^19^F NMR (282 MHz, CDCl_3_) δ −62.4 (s, 24F; C*F*_3_). HR-MS (ESI): calcd for C_19_H_20_DN_2_ [M-BArF_24_^-^]^+^: 278.1767, found: 278.1773. IR (neat, cm^-1^): *ν* 3165, 2961, 1609, 1521, 1473, 1354, 1281, 1226, 1209, 1157, 1108, 1076, 951, 933, 885, 837, 799, 773, 743, 723, 711, 681, 667, 578, 539, 521, 461, 447.


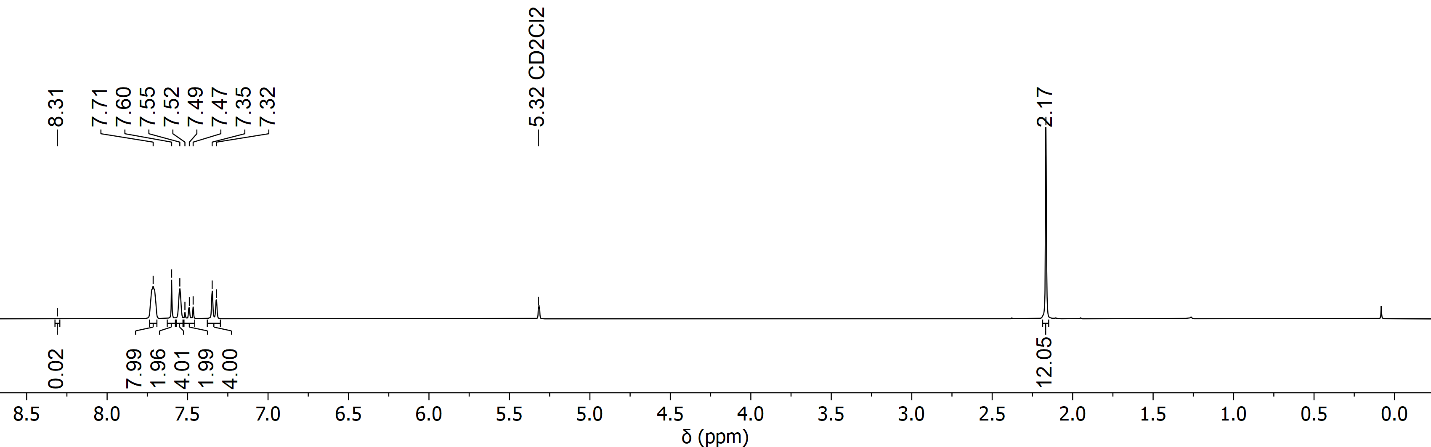


**Figure S13.** ^1^H NMR spectrum (300 MHz, CD_2_Cl_2_) of [IXylD]BArF_24_.


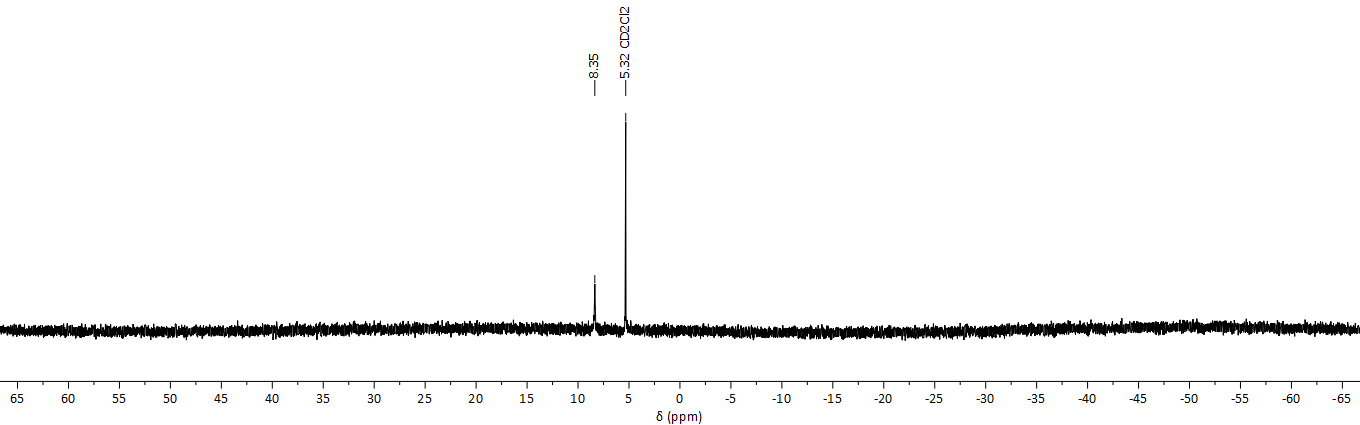


**Figure S14.** ^2^H NMR spectrum (61 MHz, CH_2_Cl_2_) of [IXylD]BArF_24_.


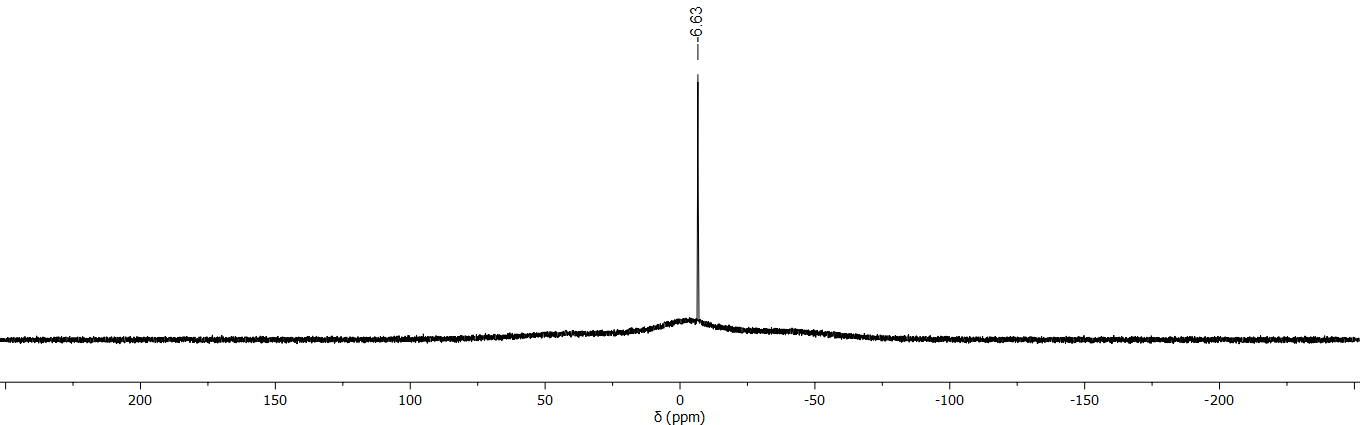


**Figure S15.** ^11^B NMR spectrum (96 MHz, CDCl_3_) of [IXylD]BArF_24_.


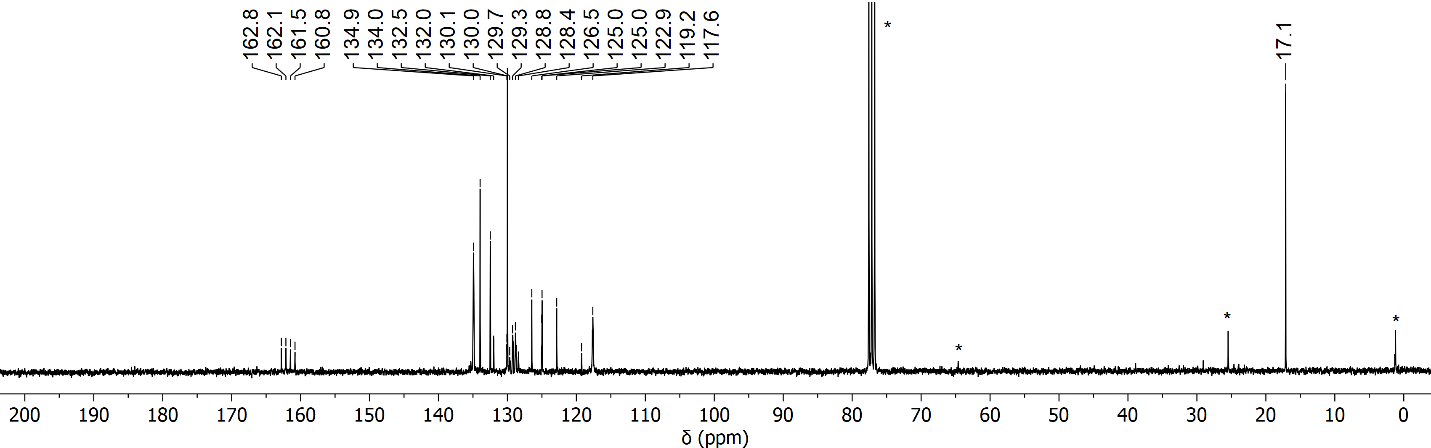


**Figure S16.** ^13^C NMR spectrum (75 MHz, CDCl_3_) of [IXylD]BArF_24_. *CDCl_3_, residual silicone grease and THF.


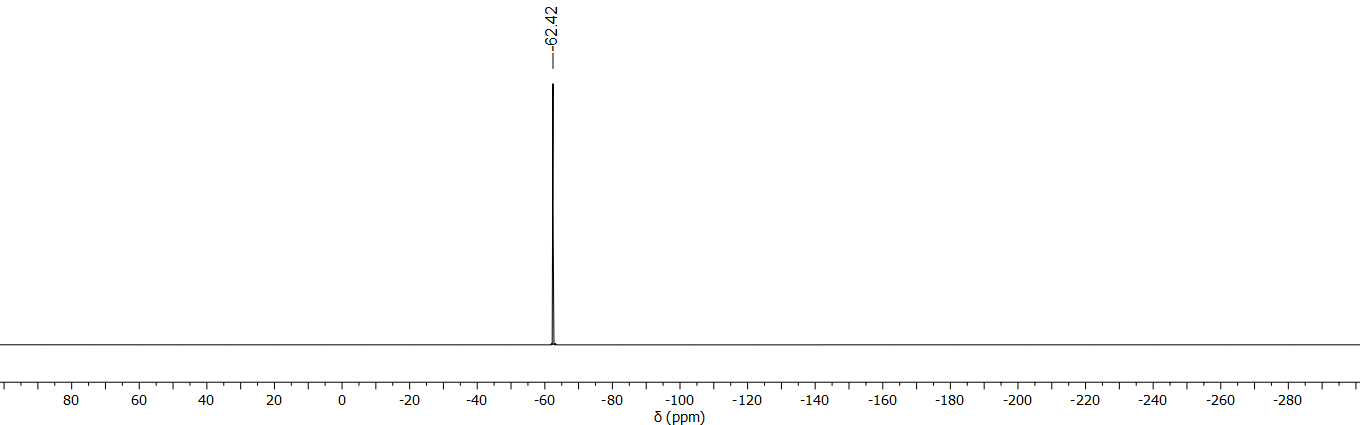


**Figure S17.** ^19^F NMR spectrum (282 MHz, CDCl_3_) of [IXylD]BArF_24_.


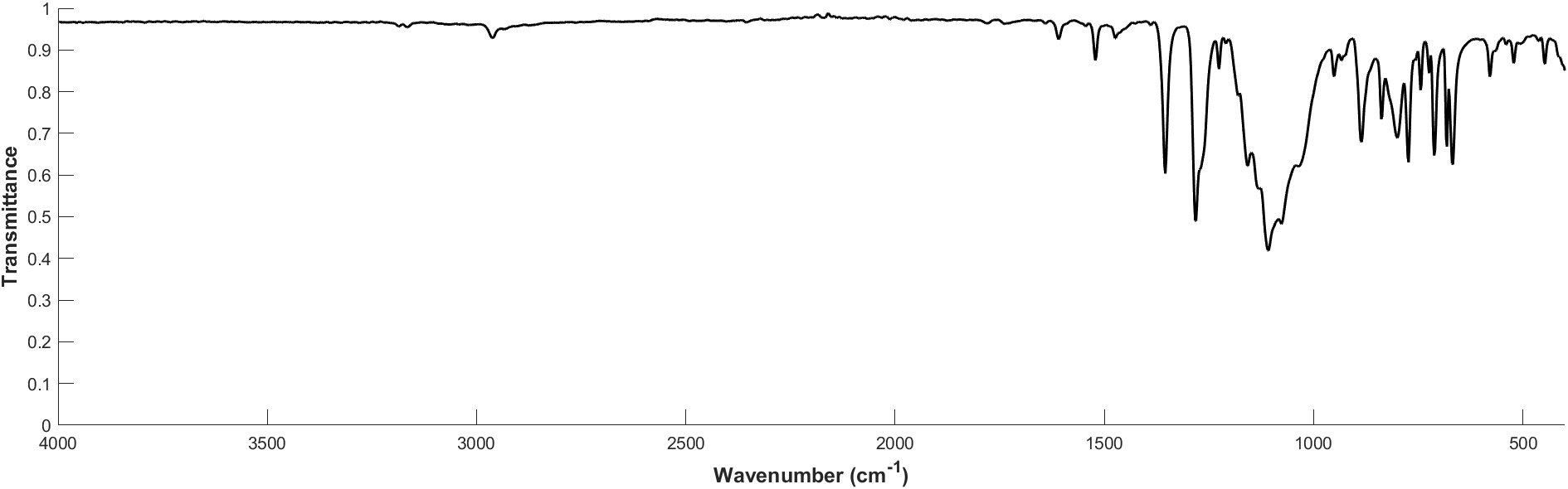


**Figure S18.** IR (neat) spectrum of [IXylD]BArF_24_.

## Synthesis of [IDippD]BArF_24_ (2-Deuterio-1,3-Bis(2,6-diisopropylphenyl)imidazolium tetrakis[3,5-bis(trifluoromethyl)phenyl]borate).

[IDippH]BArF_24_ (180 mg, 0.14 mmol) was dissolved in methanol-*d*_4_ (2 mL) in a Schlenk flask. The vessel was sealed, and the solution was stirred overnight at 50 °C. Evaporation of the solvent afforded the product as pale-yellow powder. Yield: 180 mg (0.14 mmol, 100%). m.p. = 160.5–162.6 °C. ^1^H NMR (300 MHz, CDCl_3_, 300K) δ 8.19 (t, 0.04H, residual C2-*H*), 7.72 (br s, 8H, *o*-BArF_24_*H*), 7.66 (t, ^3^*J*(H,H) = 7.9 Hz, 2H, *p*-Dipp*H*), 7.63 (s, 2H, C4,5-*H*), 7.55 (br s, 4H, *p*-BArF_24_*H*), 7.44 (d, ^3^*J*(H,H) = 7.9 Hz, 4H, *m*-Dipp*H*), 2.36 (hept, ^3^*J*(H,H) = 7.0 Hz, 4H, C*H*(CH_3_)_2_), 1.27 (d, ^3^*J* = 6.8 Hz, 12H, CH(C*H*_3_)_2_), 1.19 (d, ^3^*J* = 6.9 Hz, 12H, CH(C*H*_3_)_2_). ^2^H NMR (77 MHz, CH_2_Cl_2_) δ 8.34 (s, 1D, C2-*D*). ^11^B NMR (96 MHz, CDCl_3_) δ −6.6. ^13^C NMR (101 MHz, CDCl_3_) δ 161.8 (q, ^1^*J*(C,B) = 49.8 Hz, *ipso*-BArF_24_^-^), 144.8 (s, *o*-Dipp), 136.3 (t, ^1^*J*(C,D) = 34.0 Hz, *C*2-D), 134.9 (s, *o*-BArF_24_^-^), 133.4 (s, *p*-Dipp), 129.0 (br q, ^2^*J*(C,F) = 32.8 Hz, *m*-BArF_24_^-^), 128.8 (s, *ipso*-Dipp), 125.8 (s, *C*4,5), 125.4 (s, *m*-Dipp), 124.8 (q, ^1^*J*(C,F) = 272.7 Hz, *C*F_3_), 117.6 (s, *p*-BArF_24_^-^), 29.4 (s, *C*H(CH_3_)_2_), 24.6 (s, CH(*C*H_3_)_2_), 23.7 (s, CH(*C*H_3_)_2_). ^19^F NMR (282 MHz, CDCl_3_) δ −62.4 (s, 24F, C*F*_3_). HR-MS (ESI): calcd for C_27_H_36_DN_2_ [M-BArF_24_^-^]^+^: 390.3019, found: 390.3005. IR (neat, cm^-1^): *ν* 3152, 2968, 2932, 2347, 1643, 1610, 1517, 1465, 1391, 1352, 1329, 1271, 1216, 1184, 1160, 1118, 1059, 952, 928, 886, 837, 805, 778, 755, 743, 726, 713, 680, 668, 596, 579, 566.9, 540, 510, 446.


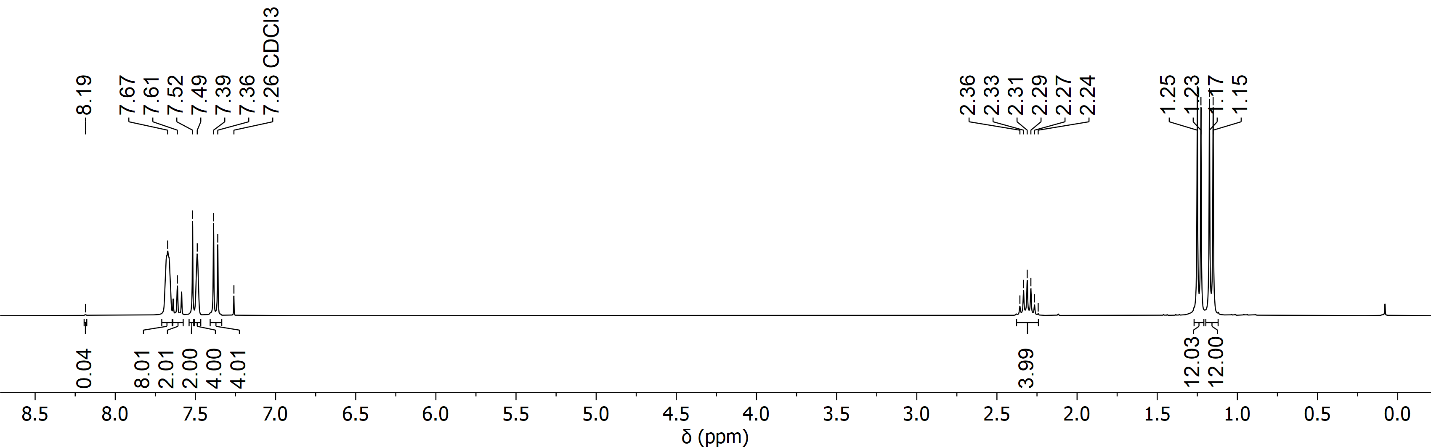


**Figure S19.** ^1^H NMR spectrum (300 MHz, CDCl_3_) of [IDippD]BArF_24_.


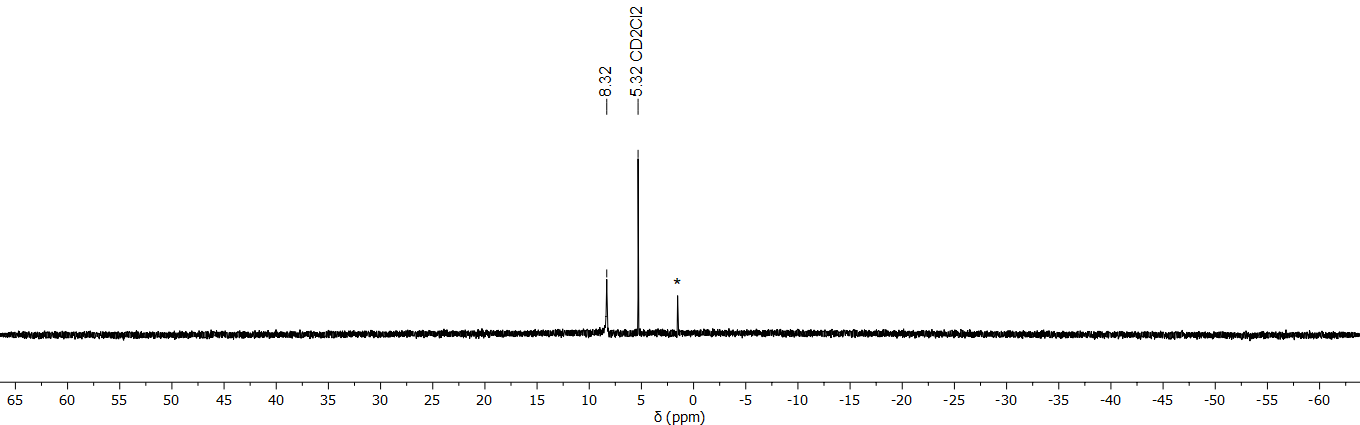


**Figure S20.** ^2^H NMR spectrum (77 MHz, CH_2_Cl_2_) of [IDippD]BArF_24_. *residual water.


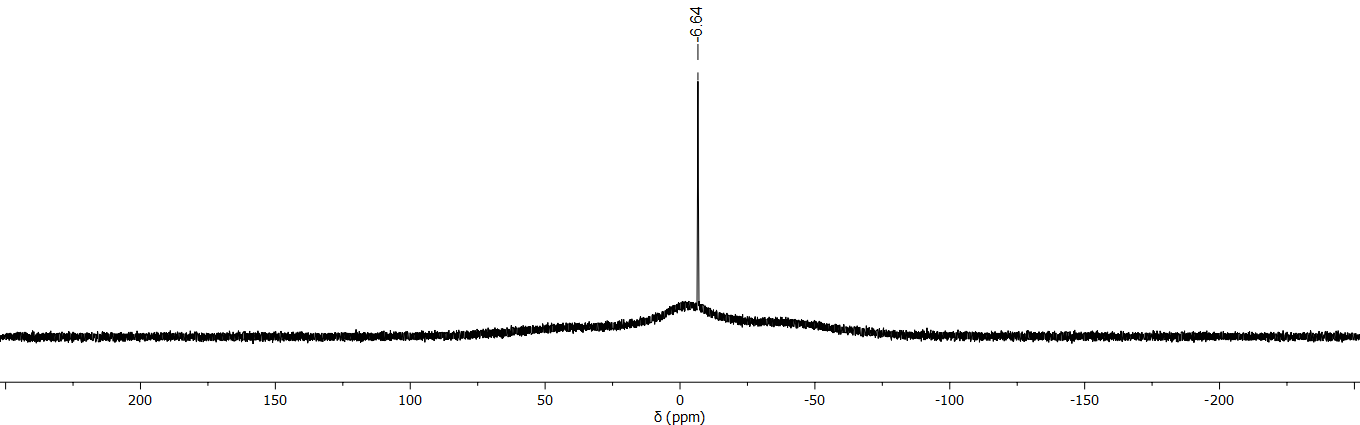


**Figure S21.** ^11^B NMR spectrum (96 MHz, CDCl_3_) of [IDippD]BArF_24_.


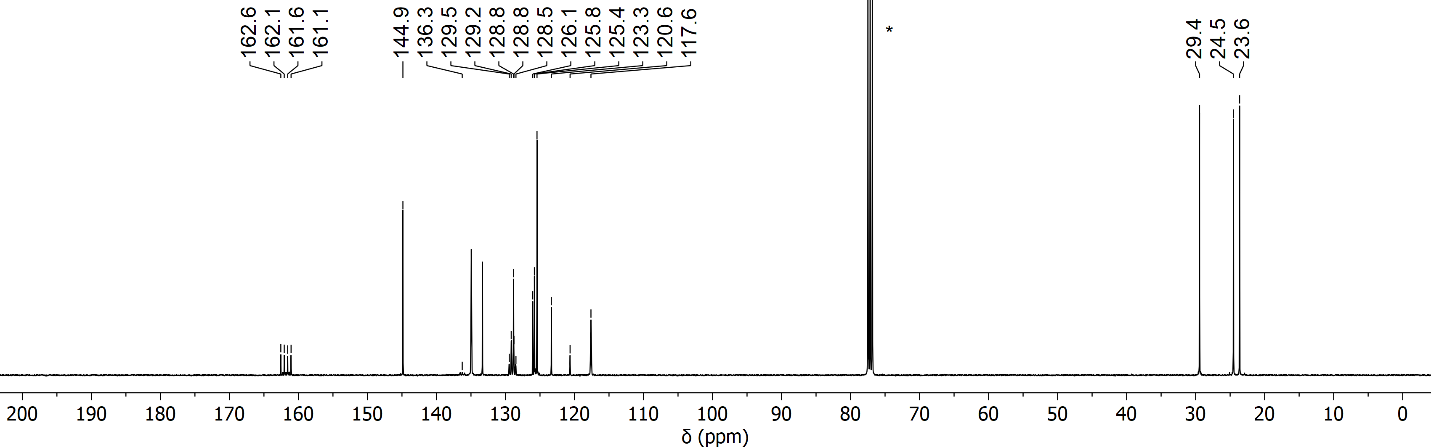


**Figure S22.** ^13^C NMR spectrum (101 MHz, CDCl_3_) of [IDippD]BArF_24_. *CDCl_3_.


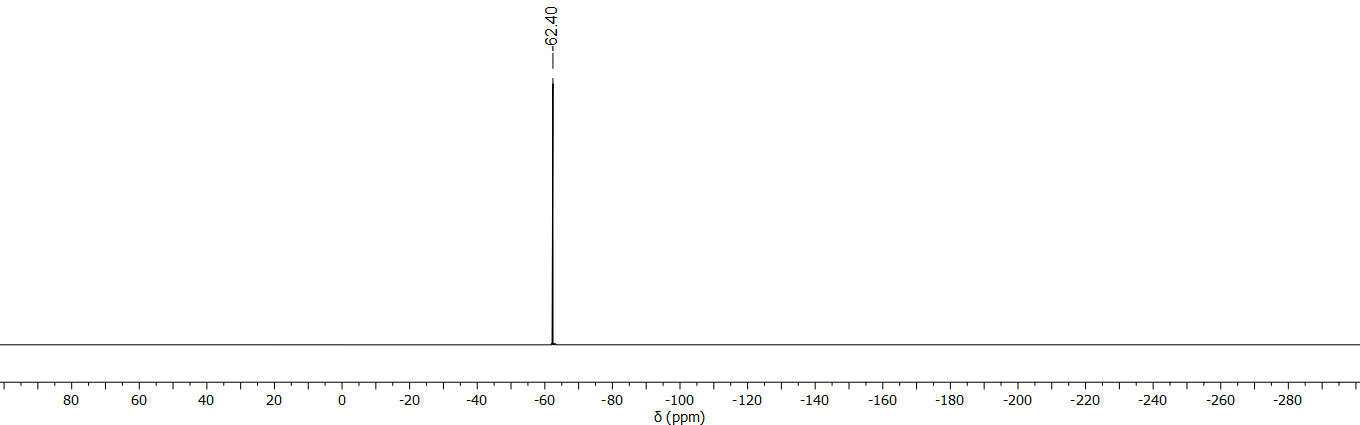


**Figure S23.** ^19^F NMR spectrum (282 MHz, CDCl_3_) of [IDippD]BArF_24_.


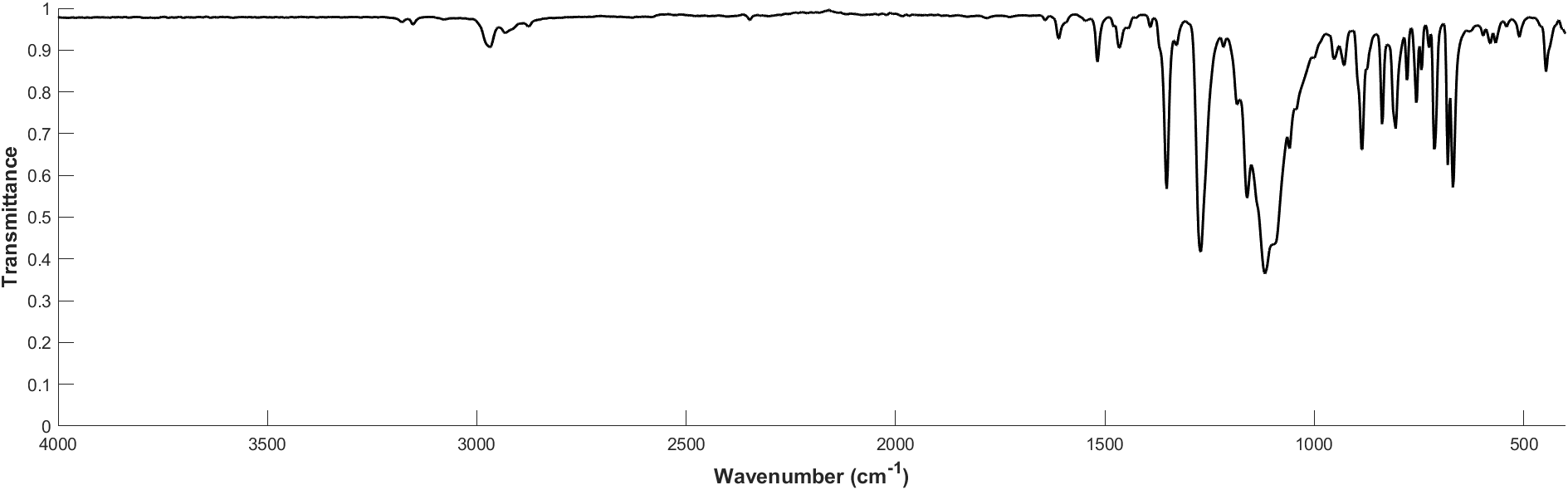


**Figure S24.** IR (neat) spectrum of [IDippD]BArF_24_.

## Synthesis of [IDippD(2,4,5-D_3_)]Cl (2,4,5-Trideuterio-1,3-Bis(2,6-diisopropylphenyl)imidazolium chloride).

[IDippD(2,4,5-D_3_)]Cl was prepared according to a literature procedure.^[3]^

^1^H NMR (300 MHz, CDCl_3_, 300K) δ 8.31 (t, 0.04H, residual C2-*H*), 7.57 (t, ^3^*J*(H,H) = 7.8 Hz, 2H, *p*-Dipp*H*), 7.35 (d, ^3^*J*(H,H) = 7.9 Hz, 4H, *m*-Dipp*H*), 2.45 (hept, ^3^*J*(H,H) = 6.8 Hz, 4H, C*H*(CH_3_)_2_), 1.28 (d, ^3^*J* = 6.8 Hz, 12H, CH(C*H*_3_)_2_), 1.24 (d, ^3^*J* = 6.9 Hz, 12H, CH(C*H*_3_)_2_). ^2^H NMR (77 MHz, CH_2_Cl_2_) δ 11.34 (s, 1D, C2-*D*), 7.79 (s, 2D, C4,5-*D*).


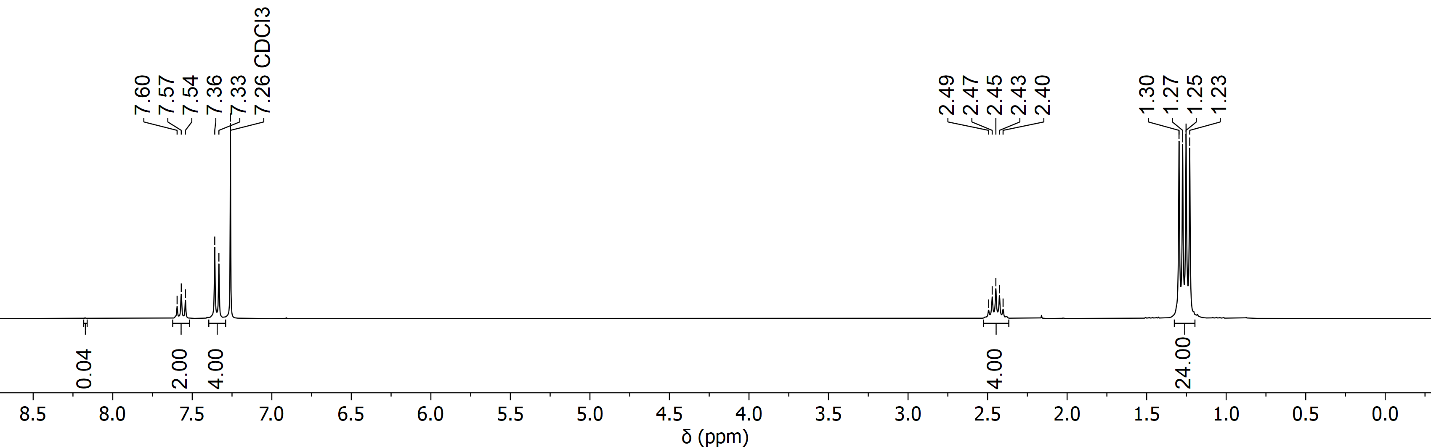


**Figure S25.** ^1^H NMR spectrum (300 MHz, CDCl_3_) of [IDippD(2,4,5-D_3_)]Cl.


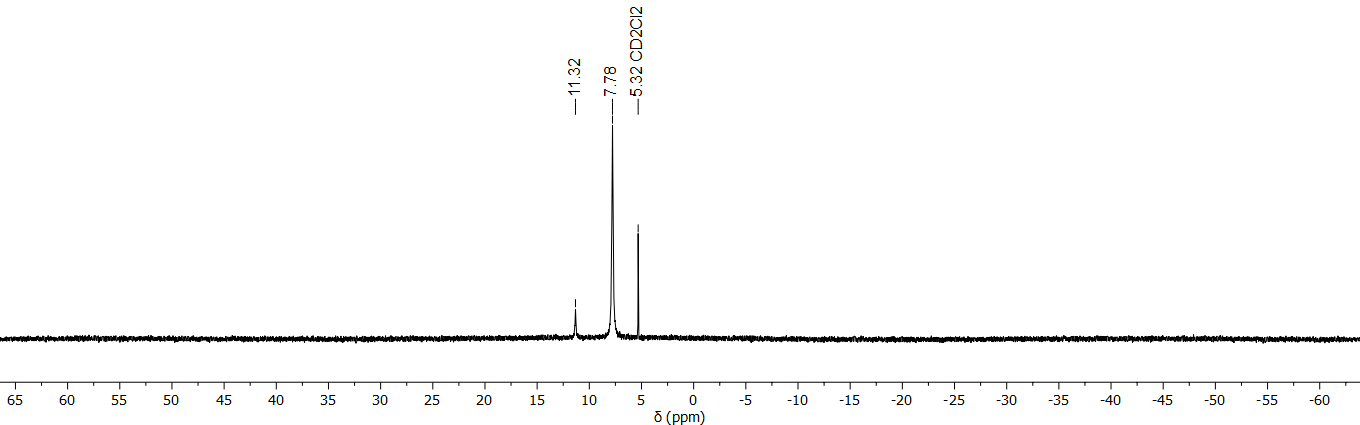


**Figure S26.** ^2^H NMR spectrum (77 MHz, CH_2_Cl_2_) of [IDippD(2,4,5-D_3_)]Cl.

## Synthesis of [IDipp(4,5-D_2_)H]BArF_24_ (4,5-Dideuterio-1,3-Bis(2,6-diisopropylphenyl)imidazolium tetrakis[3,5-bis(trifluoromethyl)phenyl]borate).

[IDippD(2,4,5-D_3_)]Cl (150 mg, 350 μmol) was dissolved in water (250 mL), and then NaBArF_24_ (311 mg, 350 μmol) was added as a solid. Dichloromethane (20 ml) was added to the mixture to solubilize the reagents, and the resulting biphasic mixture was stirred vigorously at room temperature for 30 minutes. The organic layer was separated, and the aqueous phase was extracted with dichloromethane (2 × 20 ml). The combined organic extracts were dried over anhydrous MgSO_4_, filtered, and concentrated under reduced pressure to yield the title compound as a pale-yellow powder. Yield: 400 mg, (319 μmol, 91 %).

m.p. = 158.7 – 159.8 °C. ^1^H NMR (400 MHz, CDCl_3_, 300K) δ 8.18 (s, 1H, C2-*H*), 7.67 (br s, 8H, *o*-BArF_24_*H*), 7.61 (t, ^3^*J*(H,H) = 7.9 Hz, 2H, *p*-Dipp*H*), 7.49 (br s, 4H, *p*-BArF_24_*H*), 7.38 (d, ^3^*J*(H,H) = 7.9 Hz, 4H, *m*-Dipp*H*), 2.31 (hept, ^3^*J*(H,H) = 6.8 Hz, 4H, C*H*(CH_3_)_2_), 1.24 (d, ^3^*J* = 6.9 Hz, 12H, CH(C*H*_3_)_2_), 1.17 (d, ^3^*J* = 6.9 Hz, 12H, CH(C*H*_3_)_2_). ^2^H NMR (61 MHz, CH_2_Cl_2_) δ 7.65 (s, 2D, C4,5-*D*). ^11^B NMR (96 MHz, CDCl_3_) δ −6.6. ^13^C NMR (126 MHz, CDCl_3_) δ 161.8 (q, ^1^*J*(C,B) = 49.9 Hz, *ipso*-BArF_24_^-^), 144.8 (s, *o*-Dipp), 136.4 (s, *C*2-H), 134.9 (s, *o*-BArF_24_^-^), 133.4 (s, *p*-Dipp), 129.0 (br q, ^2^*J*(C,F) = 31.7 Hz, *m*-BArF_24_^-^), 128.8 (s, *ipso*-Dipp), 125.4 (s, *m*-Dipp), 124.7 (q, ^1^*J*(C,F) = 272.5 Hz, *C*F_3_), 117.6 (s, *p*-BArF_24_^-^), 29.4 (s, *C*H(CH_3_)_2_), 24.5 (s, CH(*C*H_3_)_2_), 23.6 (s, CH(*C*H_3_)_2_). ^19^F NMR (282 MHz, CDCl_3_) δ −62.4 (s, 24F, C*F*_3_). HR-MS (ESI): calcd for C_27_H_35_D_2_N_2_ [M-BArF_24_^-^]^+^: 391.3082, found: 391.3076. IR (neat, cm^-1^): *ν* 3147, 2969, 1610, 1531, 1466, 1353, 1272, 1160, 1117, 1059, 999, 929, 906, 886, 837, 806, 791, 756, 743, 712, 681, 668, 572, 516, 446.


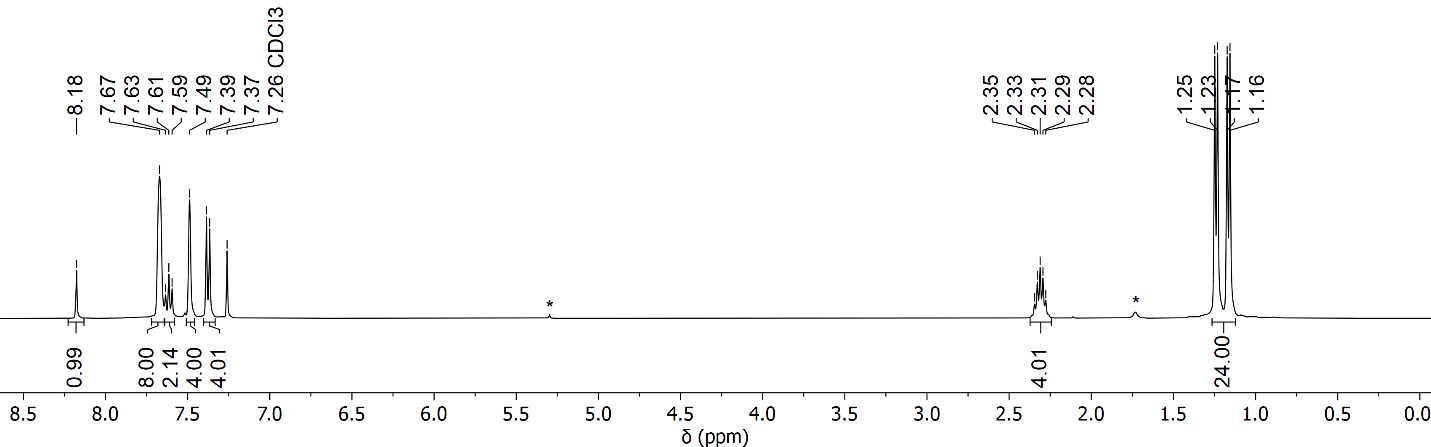


**Figure S27.** ^1^H NMR spectrum (400 MHz, CDCl_3_) of [IDipp(4,5-D_2_)H]BArF_24_.*residual DCM and water.


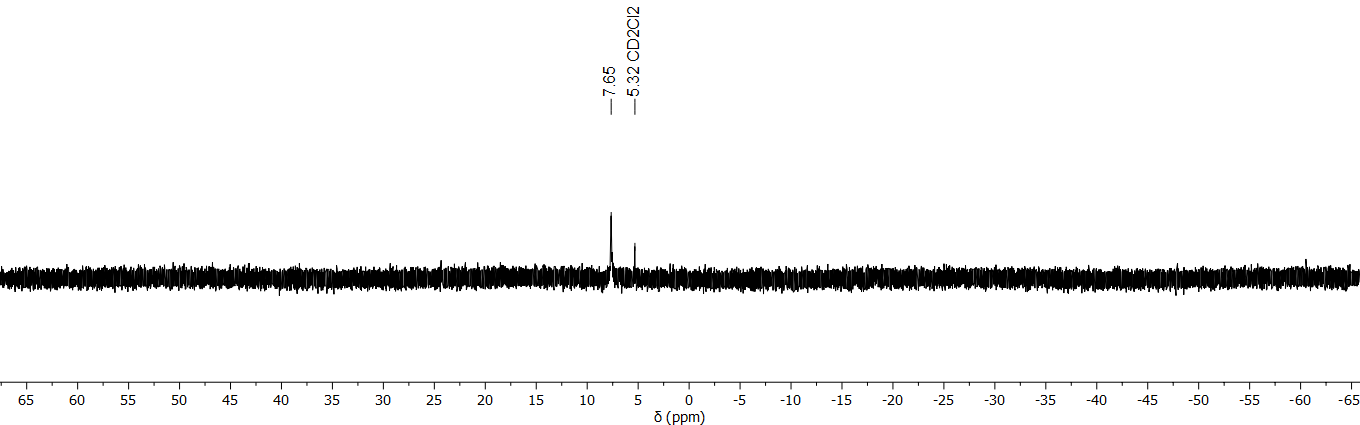


**Figure S28.** ^2^H NMR spectrum (61 MHz, CH_2_Cl_2_) of [IDipp(4,5-D_2_)H]BArF_24_.


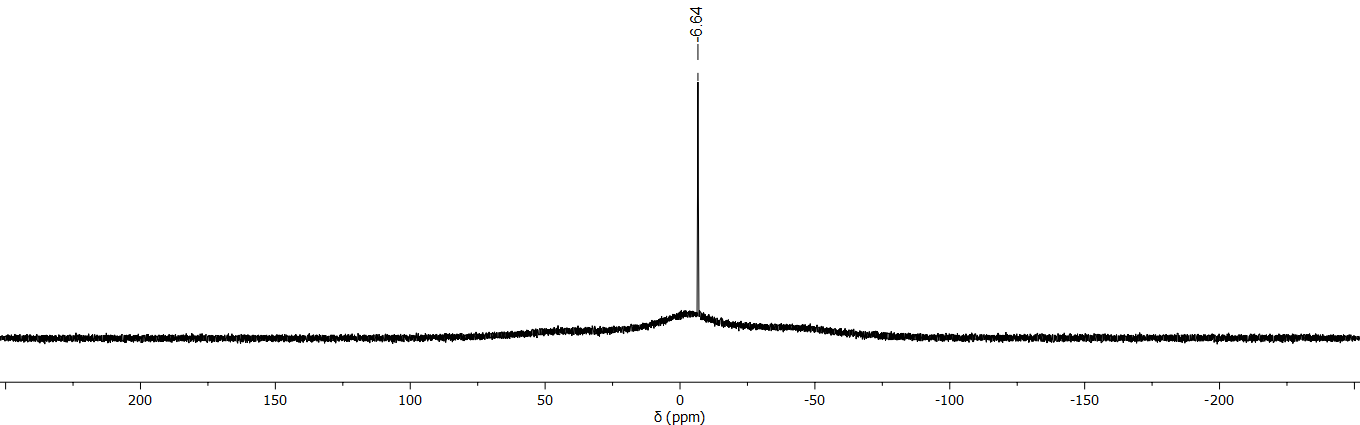


**Figure S29.** ^11^B NMR spectrum (96 MHz, CDCl_3_) of [IDipp(4,5-D_2_)H]BArF_24_.


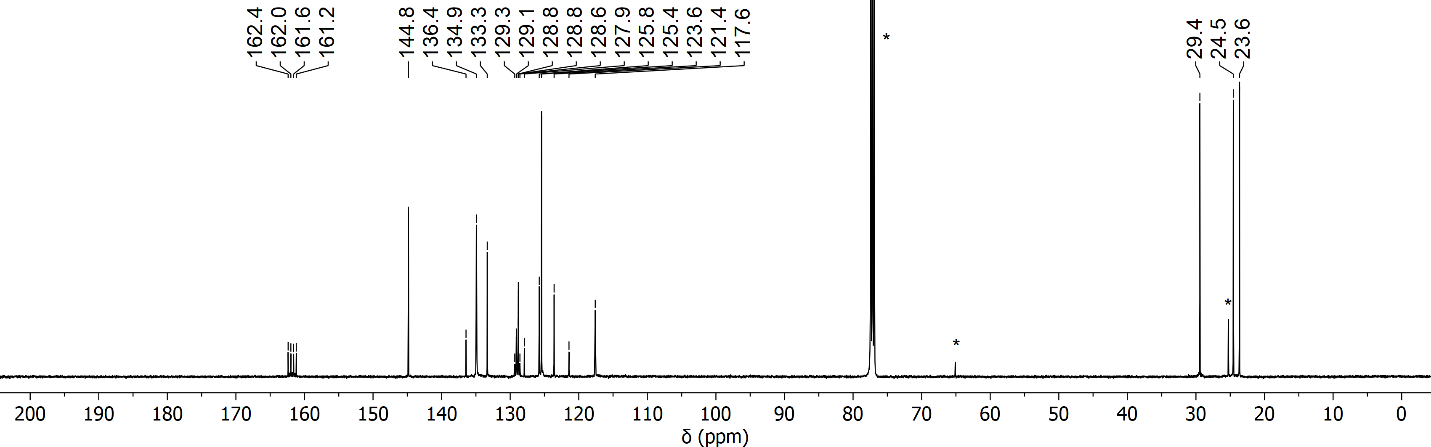


**Figure S30.** ^13^C NMR spectrum (126 MHz, CDCl_3_) of [IDipp(4,5-D_2_)H]BArF_24_. *CDCl_3_ and residual THF.


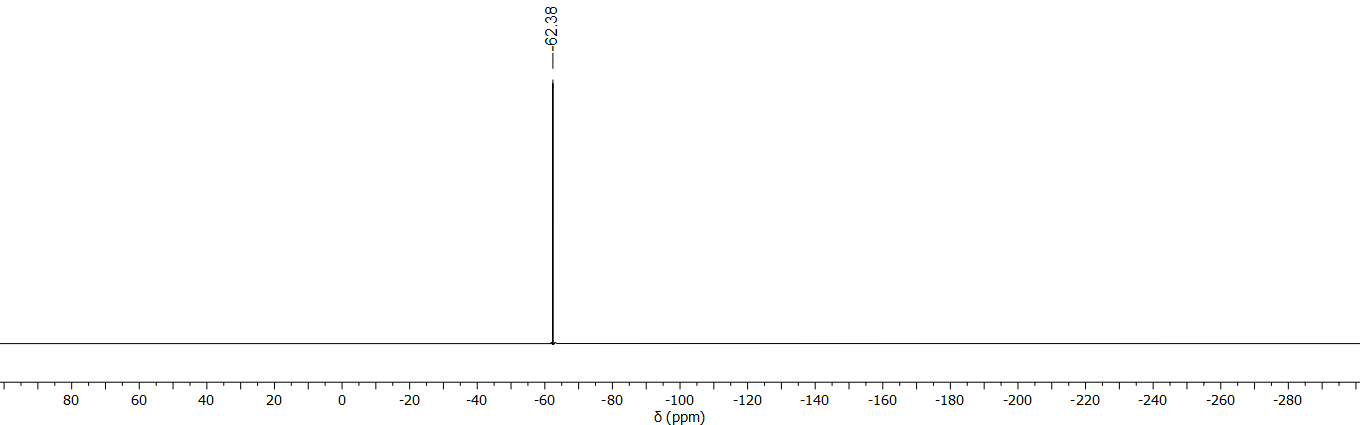


**Figure S31.** ^19^F NMR spectrum (282 MHz, CDCl_3_) of [IDipp(4,5-D_2_)H]BArF_24_.


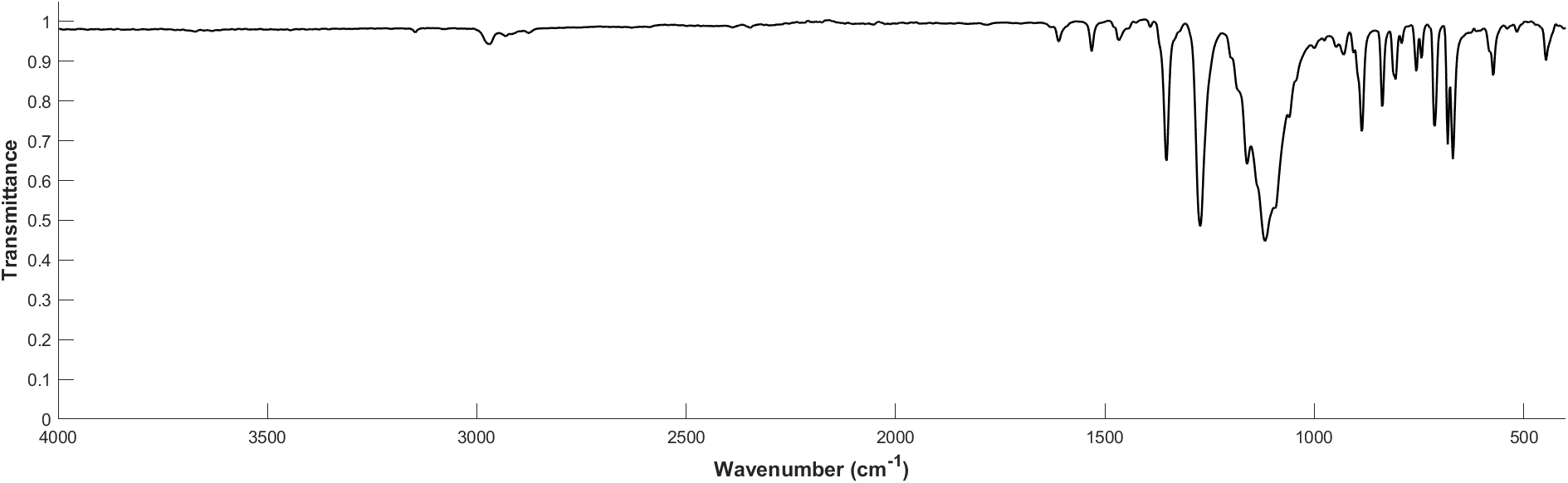


**Figure S32.** IR (neat) spectrum of [IDipp(4,5-D_2_)H]BArF_24_.

## Synthesis of tris(4-anisyl)amine ((*p*-MeOPh)_3_N).

(*p*-MeOPh)_3_N was prepared according to a literature procedure^[4]^ and recrystallized twice from ethanol before use.

1H NMR (300 MHz, C_6_D_6_, 300 K): δ 7.14–7.05 (m, 6H, *o*-Ph*H*‒OMe), 6.81–6.66 (m, 6H, *m*-Ph*H*‒OMe), 3.31 (s, 9H, OC*H*_3_).


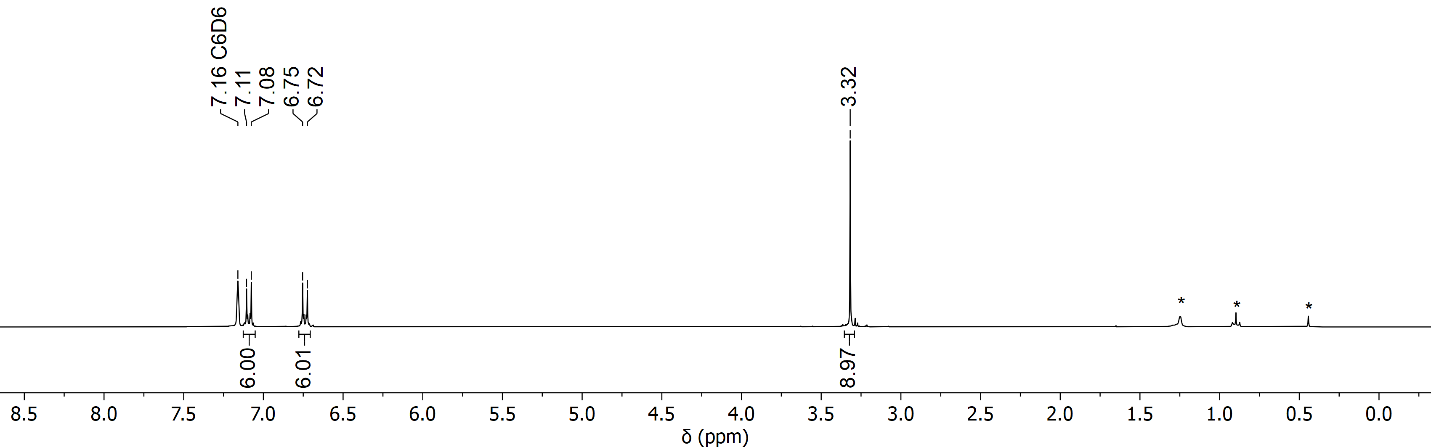


**Figure S33.** ^1^H NMR spectrum (300 MHz, C_6_D_6_) of (*p*-MeOPh)_3_N. *residual water and ethanol.

# II. Additional figures


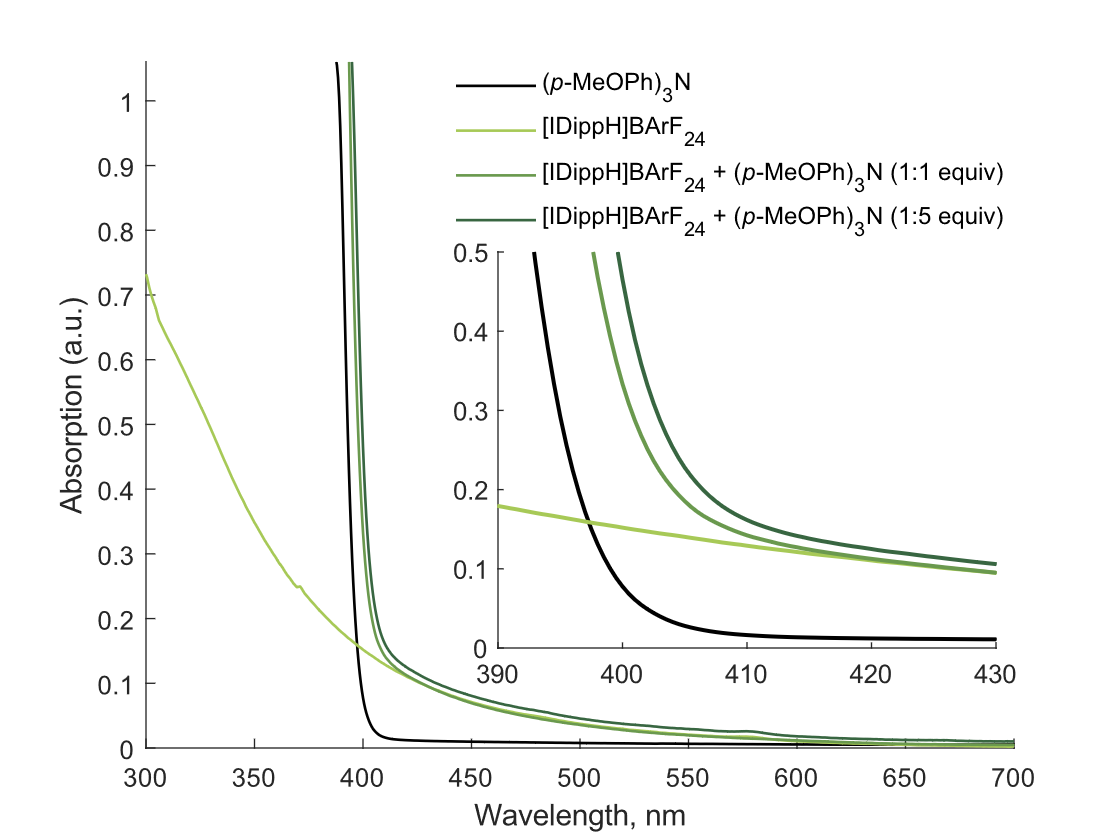


**Figure** **S34.** UV–vis spectra in toluene (298 K) of [IDippH]BArF_24_^-^ (0.015 M), (*p*-MeOPh)_3_N (0.15 M), and their mixtures (1:1 and 5:1 (*p*-MeOPh)_3_N:[IDippH]BArF_24_^-^). The mixed solutions display an additional absorption in the 400–450 nm region, which becomes more pronounced at 5:1 stoichiometry and is assigned to a donor-acceptor charge-transfer band of the EDA complex.


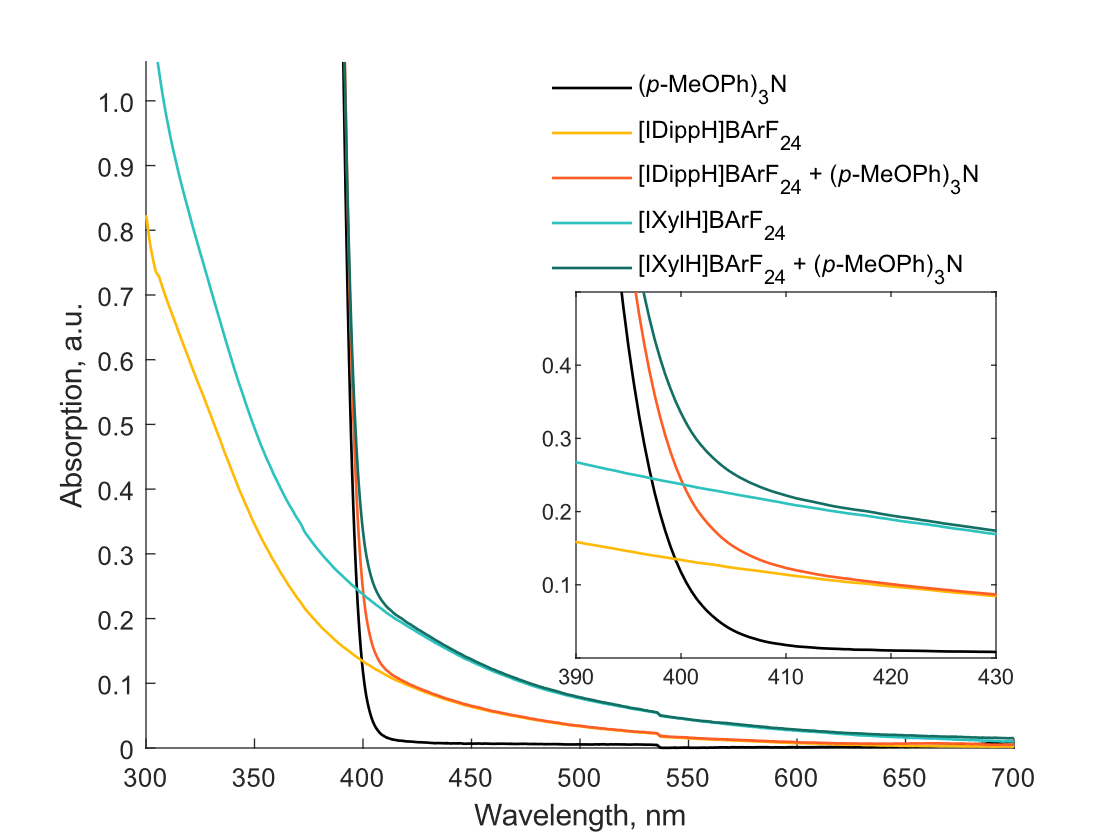


**Figure S35**. UV-vis absorption spectra of (*p*-MeOPh)_3_N (black), [IDippH]BArF_24_ (yellow), and [IXylH]BArF_24_ (teal) in 2-methyltetrahydrofuran (all 0.015 M), together with the corresponding equimolar mixtures of the imidazolium salt and donor (orange and dark green, respectively).


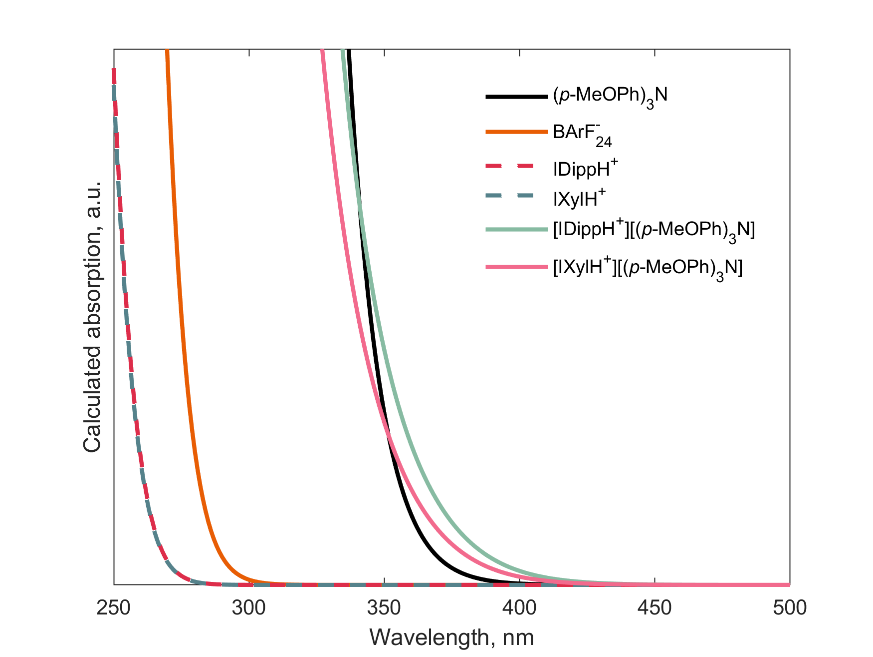


**Figure S36.** Calculated UV–vis spectra simulated by TD-DFT at the *ω*B97X-D/6 311++G(d,p) level of theory (solvent: toluene) for optimized structures of (*p-*MeOPh)_3_N, IDippH^+^, IXylH^+^, and the corresponding [imidazolium···(*p-*MeOPh)_3_N] encounter complexes. The computed spectrum of the complex closely matches the band pattern of (*p-*MeOPh)_3_N but exhibits systematically larger intensities (increased oscillator strengths), while IDippH^+^ and IXylH^+^ show essentially no absorption beyond 300 nm.


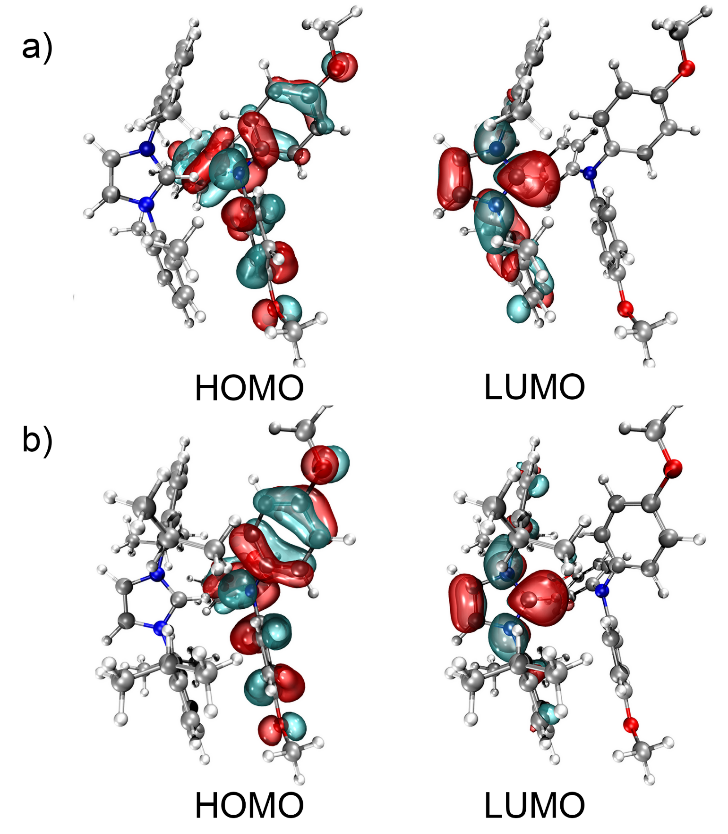


**Figure S37**. Frontier molecular orbitals of IXylH^+^/(*p*-MeOPh)_3_N (a) and IDippH^+^/(*p*-MeOPh)_3_N (b) encounter complexes (*ω*B97X-D/6-311G(d,p)).


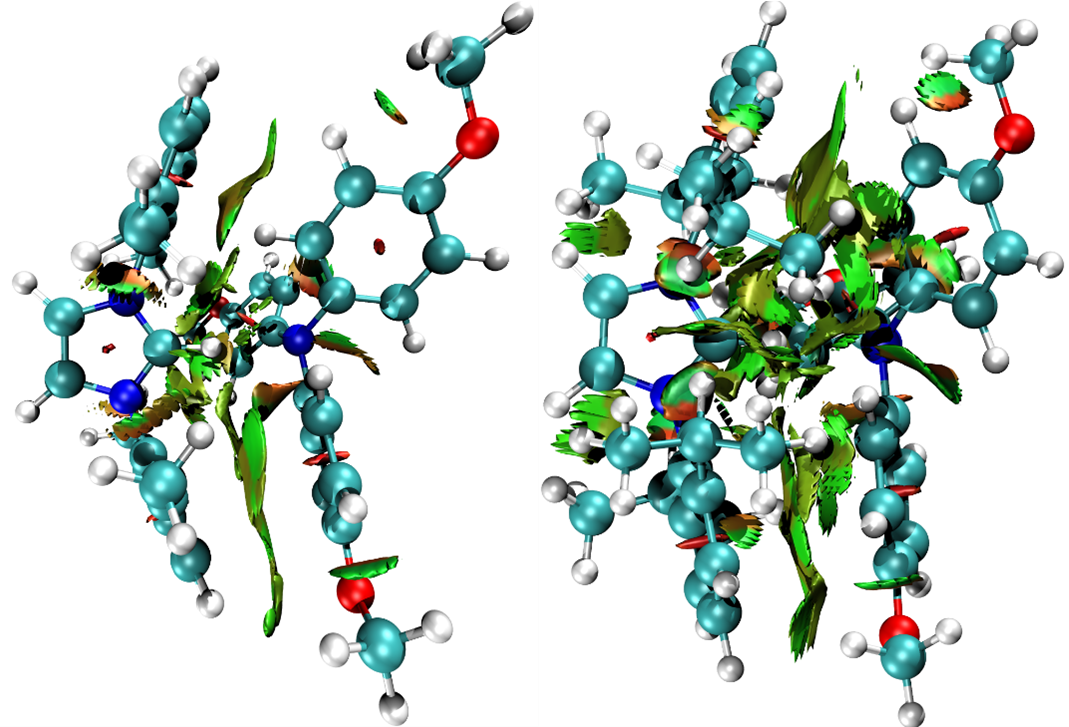


**Figure S38.** Reduced density gradient (RDG) isosurfaces for the calculated encounter complexes IXylH^+^/(*p*-MeOPh)_3_N (left) and IDippH^+^/(*p*-MeOPh)_3_N (right), colored by sign(λ2)ρ, highlighting intermolecular non-covalent interaction regions between the imidazolium aryl substituents and the anisyl rings of (*p*-MeOPh)_3_N.


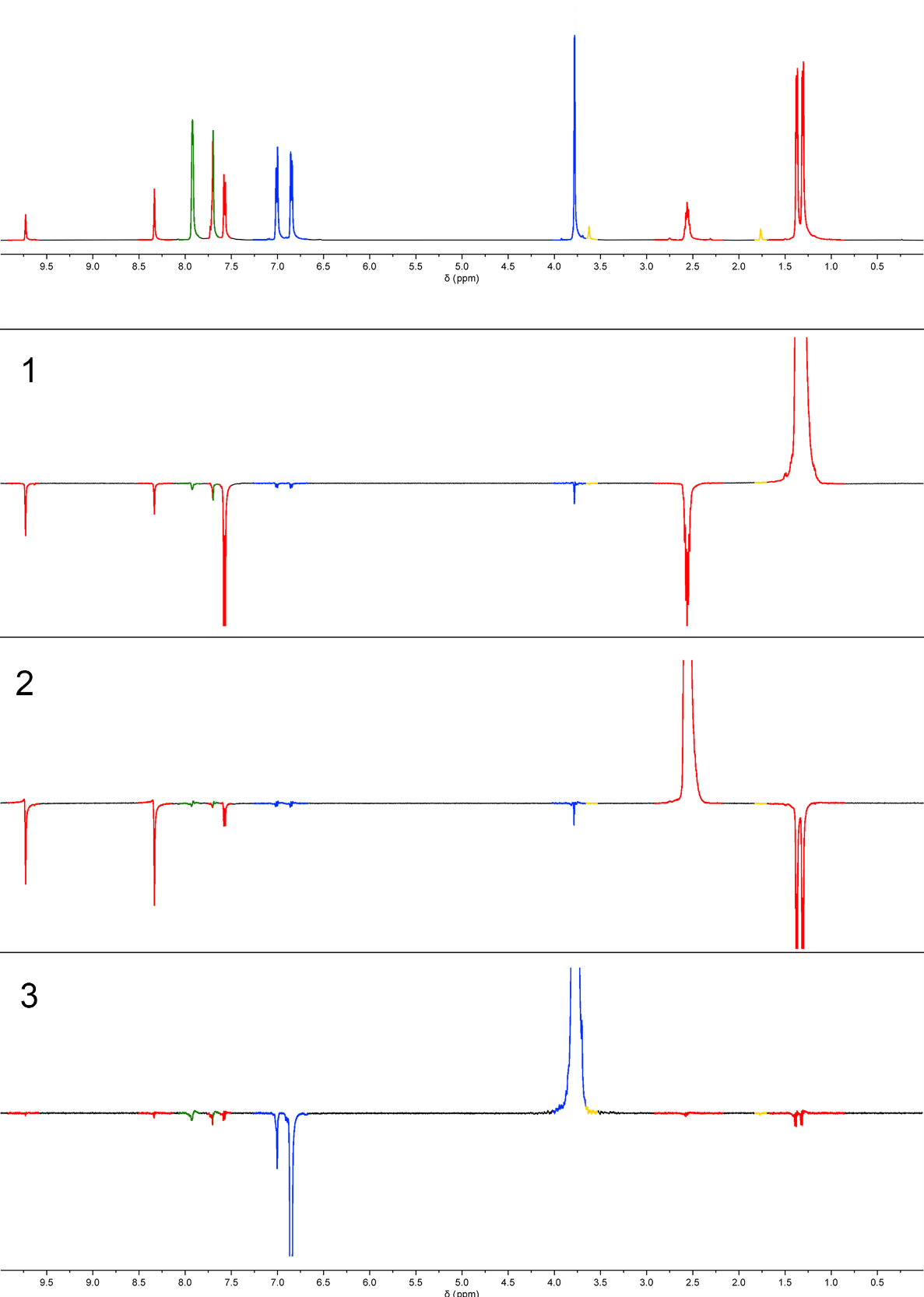


**Figure S39.** 1D NOESY of IDippH^+^/(*p*-MeOPh)_3_N EDA pair. Top: ^1^H NMR spectrum of [IDippH]BArF_24_ and (*p*-MeOPh)_3_N in THF-d_8_ (200 mM each, 300 K). Bottom: 1D selective NOESY spectra recorded with a mixing time *τ*_m_ = 100 ms, with selective irradiation at (1) δ 1.27–1.35 ppm (CH_3_, IDippH^+^), (2) δ 2.50–2.55 ppm (CH, IDippH^+^), and (3) δ 3.73–3.75 ppm (OMe, (*p*-MeOPh)_3_N). In each experiment, NOE responses are observed on resonances belonging to the other molecular fragment, consistent with close spatial proximity of the donor and acceptor in solution. ^1^H NMR resonances are color coded: IDippH^+^ (red), (*p*-MeOPh)_3_N (blue), BArF_24_^-^ (green), and residual THF (yellow).


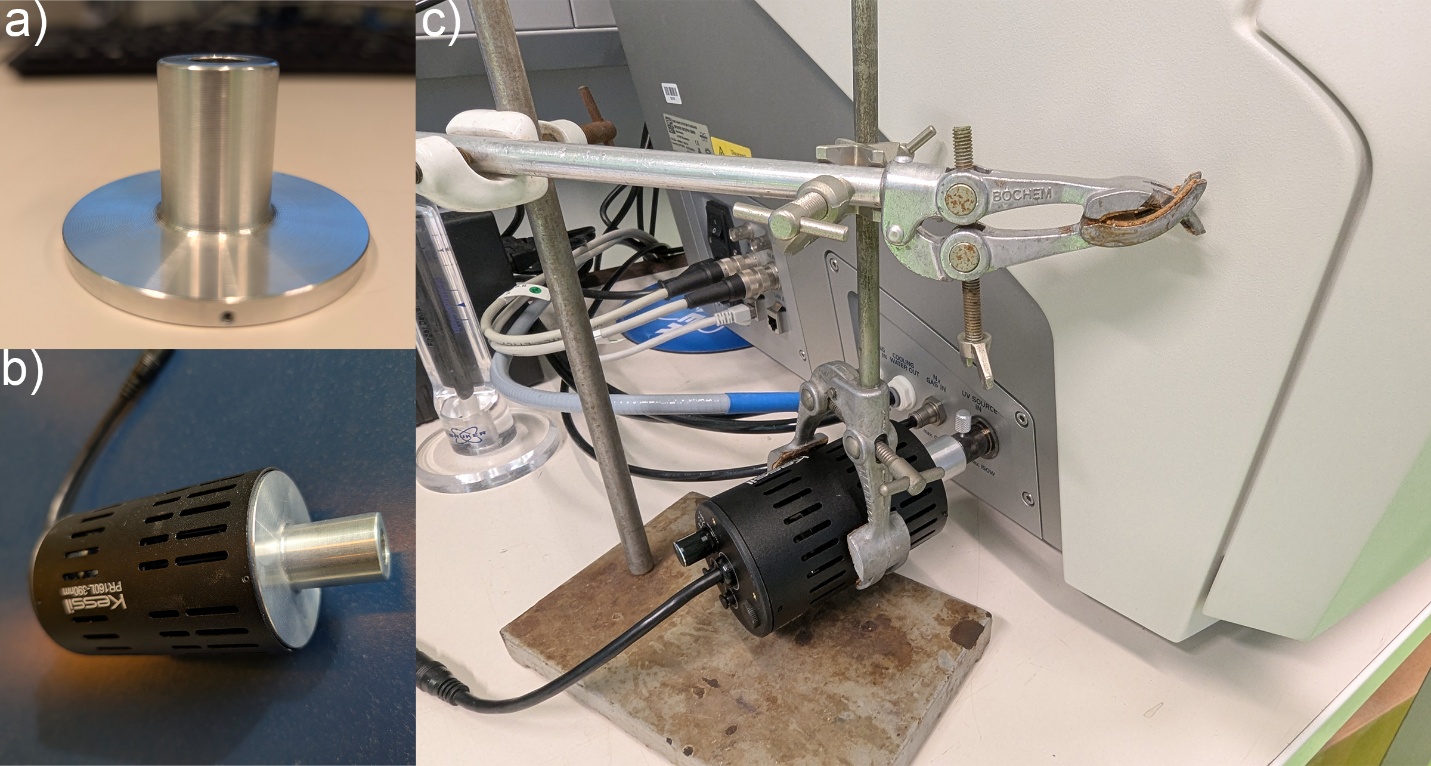


**Figure S40.** Photographs of the *in situ* irradiation setup used for EPR experiments: a) custom-made aluminum adapter; b) Kessil lamp fitted with the adapter; c) Kessil lamp fitted with the adapter and mounted to the EPR spectrometer. This arrangement enabled *in situ* irradiation of the sample during EPR measurements.


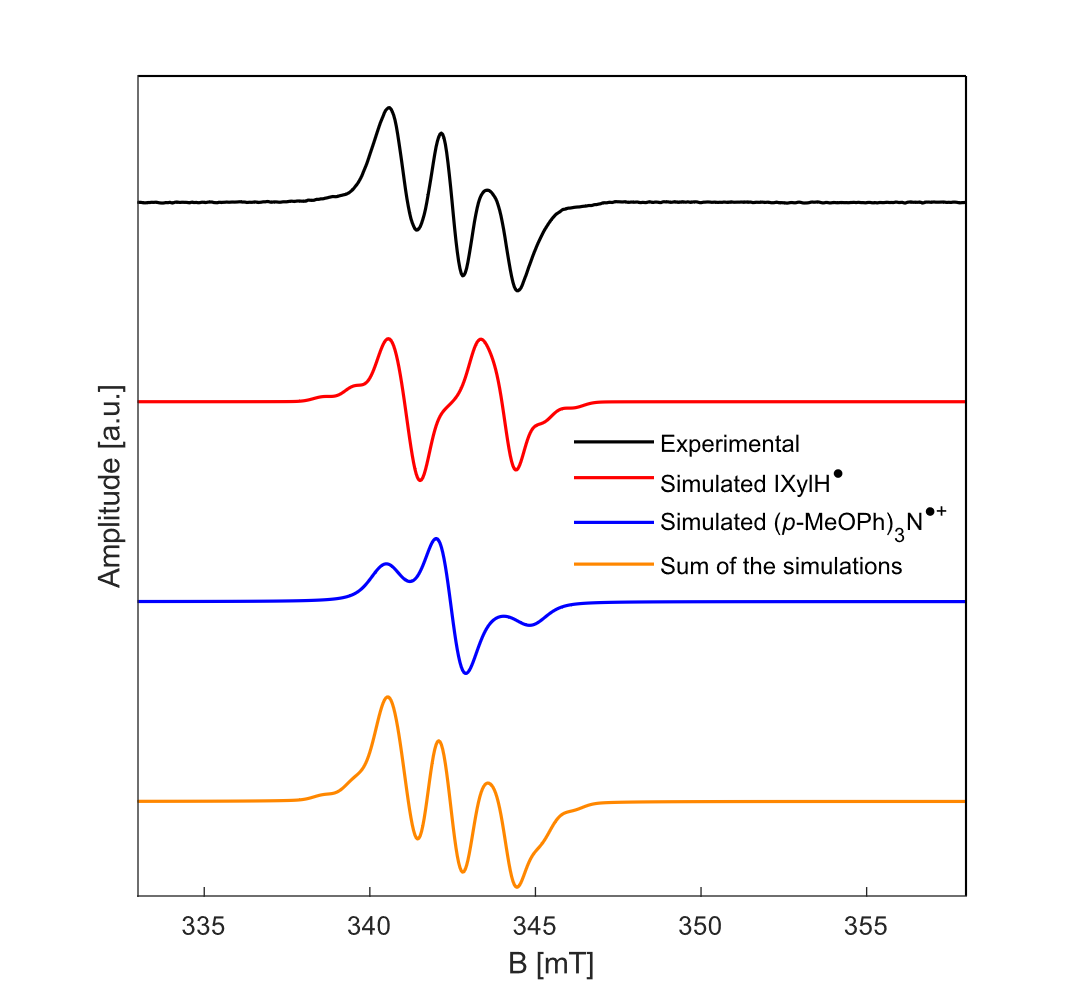


**Figure S41.** Frozen-matrix (100 K) X-band EPR spectrum of an irradiated 2-methyltetrahydrofuran solution of [IXylH]BArF_24_ (0.15 M) and (*p-*MeOPh)_3_N (0.15 M), together with simulations showing the presence of [IXylH]^•^ and [(*p-*MeOPh)_3_N]^•+^. Simulation parameters: [IXylH]^•^: *g* = [2.0041 2.0030 2.0041], *A*^H^ = [67 88 108] MHz (C2-*H*), 2×*A*^N^ = [1 1 26] MHz, lwpp = 0.558467 & 0.1461973 (Gaussian & Lorentzian) weight = 0.53. [(*p-*MeOPh)_3_N]^•+^: *g* = [2.0046 2.0046 2.0025], *A*^N^ = [1 1 61] MHz, lwpp = 0.608758 & 0.369051 (Gaussian & Lorentzian), weight = 1. Microwave frequency = 9.6054 GHz, power = 0.3162 mW, modulation amplitude = 4 G.


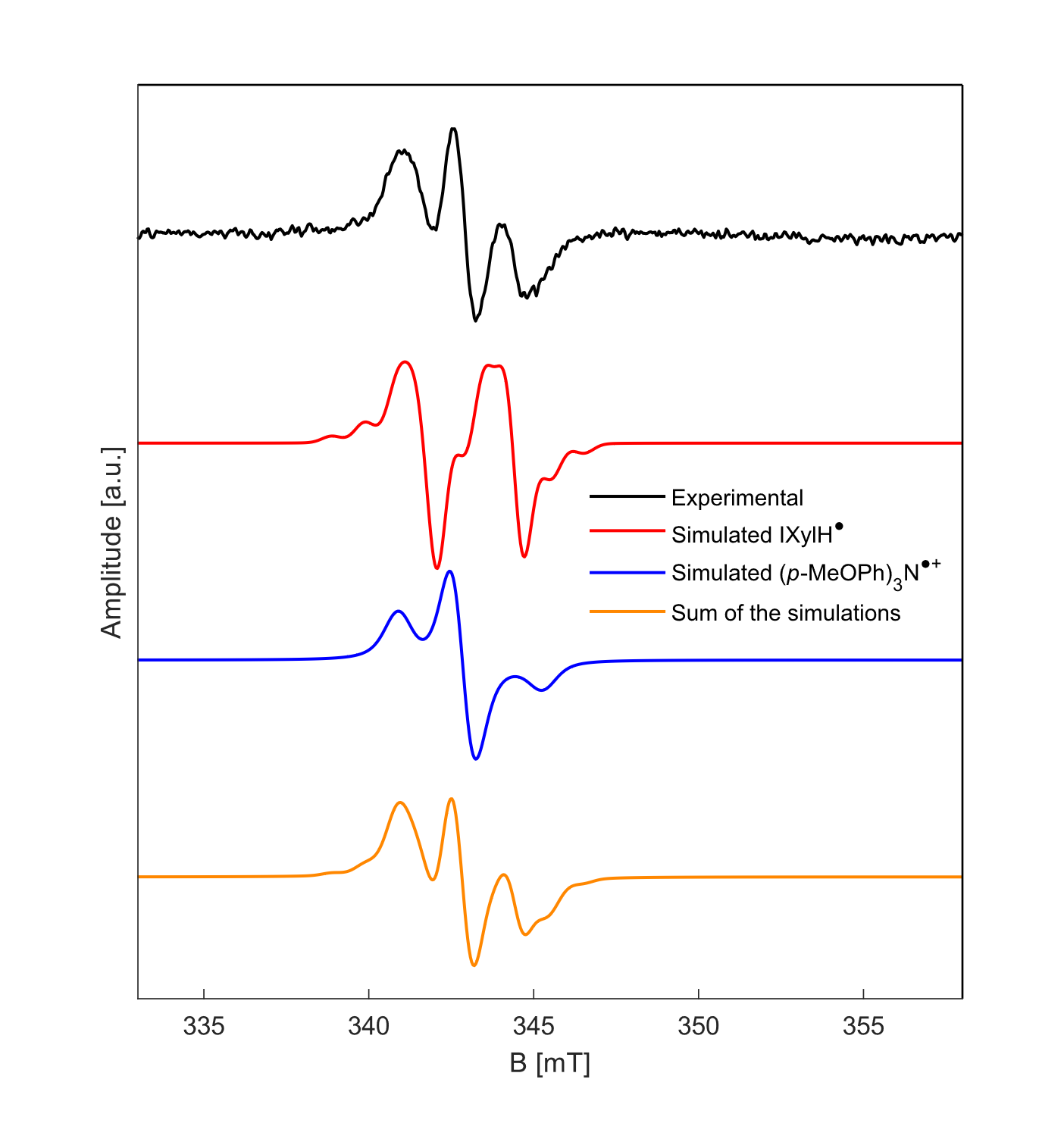


**Figure S42.** Frozen-matrix (100 K) X-band EPR spectrum of an irradiated toluene solution of [IXylH]BArF_24_ (0.15 M) and (*p-*MeOPh)_3_N (0.15 M), together with simulations showing the presence of [IXylH]^•^ and [(p-MeOPh)_3_N]^•+^. Simulation parameters: [IXylH]^•^: g = [2.0038 2.0026 2.0048] A^H^ = [60 83 103] MHz (C2-*H*), 2×*A*^N^ = [1 1 28] MHz, lwpp= 0.508485 & 0.0461882 (Gaussian & Lorentzian), weight = 0.30. [(*p-*MeOPh)_3_N]^•+^: g = [2.0046 2.0046 2.0025], A^N^ = 1 61, lwpp = 0.474546 & 0.387262 (Gaussian & Lorentzian), weight = 1. Microwave frequency = 9.6166 MHz, microwave power = 0.3162 mW, modulation amplitude = 4 G.


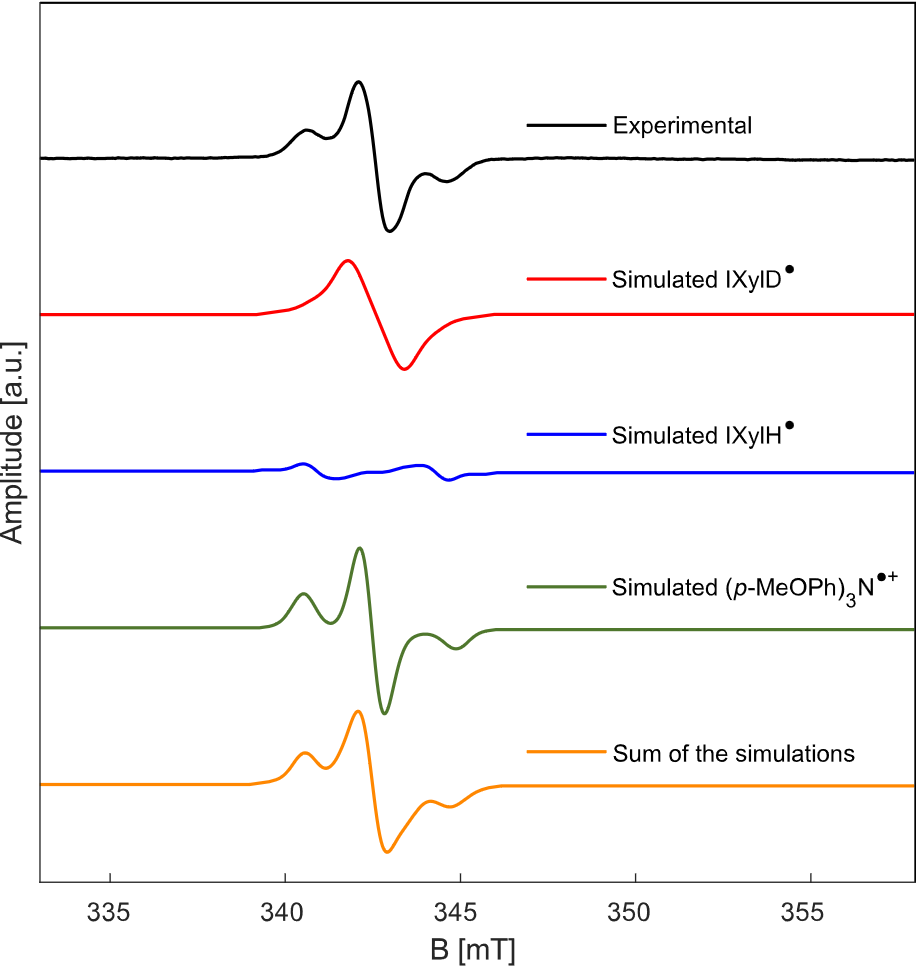


**Figure S43.** Frozen-matrix (100 K) X-band EPR spectrum of an irradiated *in situ* 2-methyltetrahydrofuran solution of [IXylD]BArF_24_ (0.15 M) and (*p-*MeOPh)_3_N (0.15 M), together with simulations showing the presence of [IXylD]^•^, minor amounts of [IXylH]^•^ and [(*p-*MeOPh)_3_N]^•+^. Simulation parameters: [IXylD]^•^: *g* = [2.0041 2.0031 2.0043], *A*^D^ = [10 13 17] MHz (C2-*D*), 2×*A*^N^ = [1 1 29] MHz, lwpp = 0.511769 & 0.04619 (Gaussian & Lorentzian) weight = 0.88; [IXylH]^•^: *g* = [2.0041 2.0031 2.0043], *A*^H^ = [66 88 108] MHz (C2-*H*), 2×*A*^N^ = [1 1 26] MHz, lwpp = 0.56793 & 0.04619 (Gaussian & Lorentzian) weight = 0.12. [(*p-*MeOPh)_3_N]^•+^: *g* = [2.0046 2.0046 2.0025], *A*^N^ = [1 1 61] MHz, lwpp = 0.54111 & 0.16915 (Gaussian & Lorentzian), weight = 1. Microwave frequency = 9.6068 GHz; microwave power = 0.3162 mW; modulation amplitude = 4 G.


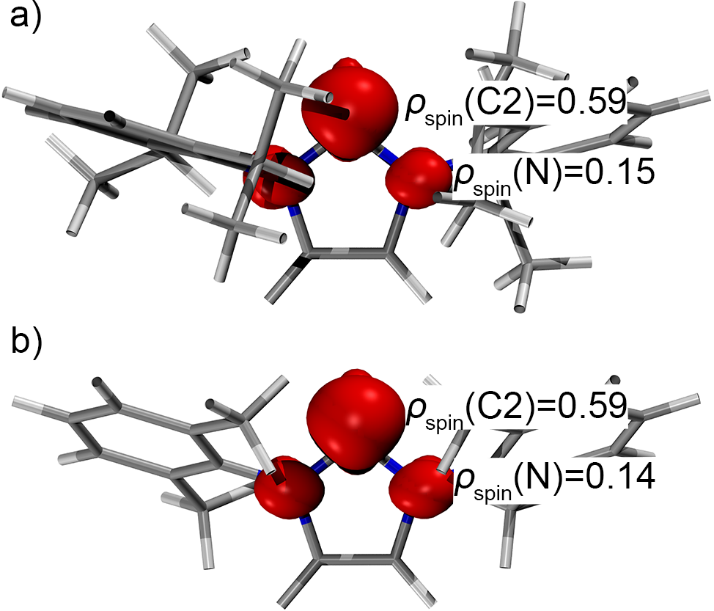


**Figure S44.** Mulliken spin densities of [IDippH]^•^ (a) and [IXylH]^•^ (b).


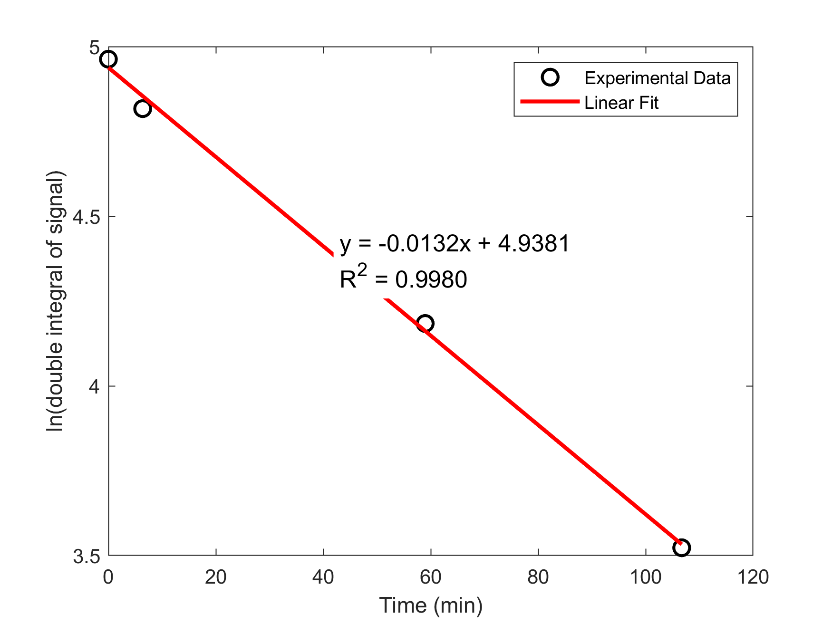


**Figure** **S45.** The radical concentration monitored by EPR spectroscopy using the double-integrated EPR signal of irradiated (390 nm) for 20 minutes solution of [IDippH]BArF_24_ and (*p*-MeOPh)_3_N in toluene over time after switching off the irradiation. Assuming first-order decay in $\mathrm{IDipp}H_{2}^{\cdot+}$: $\ln\left( A_{t} \right)=-\mathrm{kt}+\ln\left( A_{0} \right)$, giving $k=0.0132 \mathrm{mi}n^{-1}$, $R^{2}=0.998$; $\tau=1/k\approx76\text{\textbackslash min}$. Experimental details: power = 0.3162 mW, modulation amplitude = 4 G.


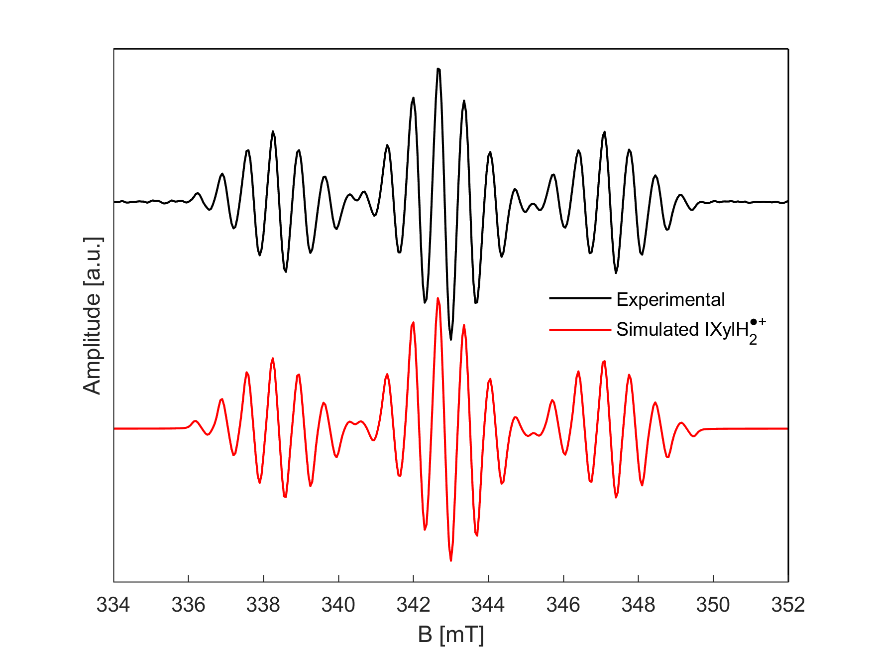


**Figure S46.** Room-temperature X-band EPR spectrum of an irradiated toluene solution of [IXylH]BArF_24_ (0.15 M) and (*p*-MeOPh)_3_N (0.15 M), recorded after 10 minutes after the start of irradiation (390 nm light) with the simulation. Simulation parameters: [IXylH_2_]^•+^: *g*_iso_ = 2.0029, 2×*A*^H^_iso_ = 123.9 MHz, 2×*A*^N^_iso_ = 18.9 MHz, 2×*A*^H^_iso_ = 19.7 MHz, lwpp = 0.12184 & 0.06762 (Gaussian & Lorentzian). Microwave frequency = 9.6116 GHz, power = 0.3162 mW, modulation amplitude = 4 G.


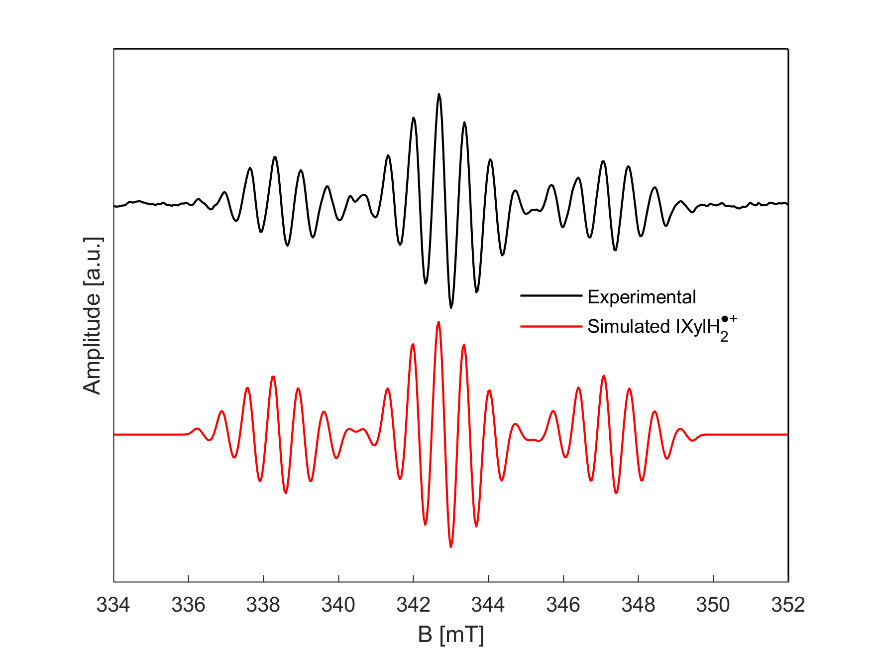


**Figure S47.** Room-temperature X-band EPR spectrum of an irradiated 2-methyltetrahydrofuran solution of [IXylH]BArF_24_ (0.15 M) and (*p-*MeOPh)_3_N (0.15 M), together with a simulation showing the presence of [IXylH_2_]^•+^. Simulation parameters: [IXylH_2_]^•+^: *g*_iso_ = 2.0029, 2×*A*^H^_iso_ = 122.5 MHz (C2-*H*), 2×*A*^N^_iso_ = 18.8 MHz, 2×*A*^H^_iso_ = 19.7 MHz (C4,5-*H*), lwpp = 0.217936 5 & 0.0119103 (Gaussian & Lorentzian). Microwave frequency = 9.6118 GHz, power = 0.3162 mW, modulation amplitude = 4 G.


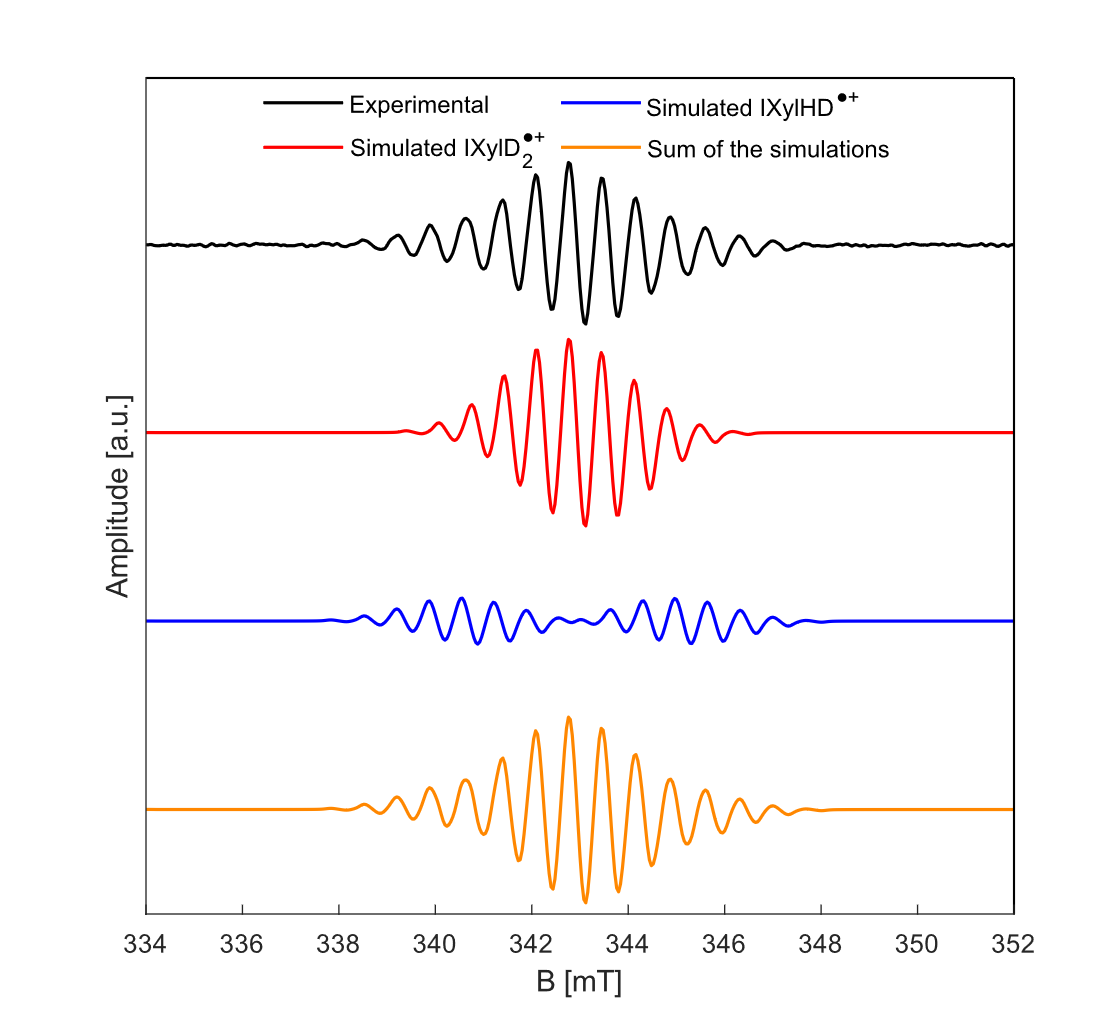


**Figure S48.** Room-temperature X-band EPR spectrum of an irradiated toluene solution of [IXylD]BArF_24_ (0.15 M) and (*p*-MeOPh)_3_N (0.15 M) with a simulation showing the presence of [IXylD_2_]^•+^ and [IXylHD]^•+^. Simulation details: [IXylD_2_]^•+^: *g*_iso_ = 2.0031, 2×*A*^D^_iso_ = 18.9 MHz (C2-*D*), 2×*A*^N^_iso_ = 18.2 MHz, 2×*A*^H^_iso_ = 20.7 MHz (C4,5-*H*), lwpp = 0.1185 & 0.0662 (Gaussian & Lorentzian); [IXylHD]^•+^: *g*_iso_ = 2.0031, *A*^D^_iso_ = 18.8 MHz (C2-*D*), *A*^H^_iso_ = 124.2 MHz (C2-*H*), 2×*A*^N^_iso_ = 18.4 MHz, 2×*A*^H^_iso_ = 20.1 MHz (C4,5-*H*), lwpp=0.1104 & 0.0922 (Gaussian & Lorentzian). Ratio [IXylD_2_]^•+^:[IXylHD]^•+^ 1:0.48. Microwave frequency = 9.6146 GHz, power = 0.3162 mW, modulation amplitude = 4 G.


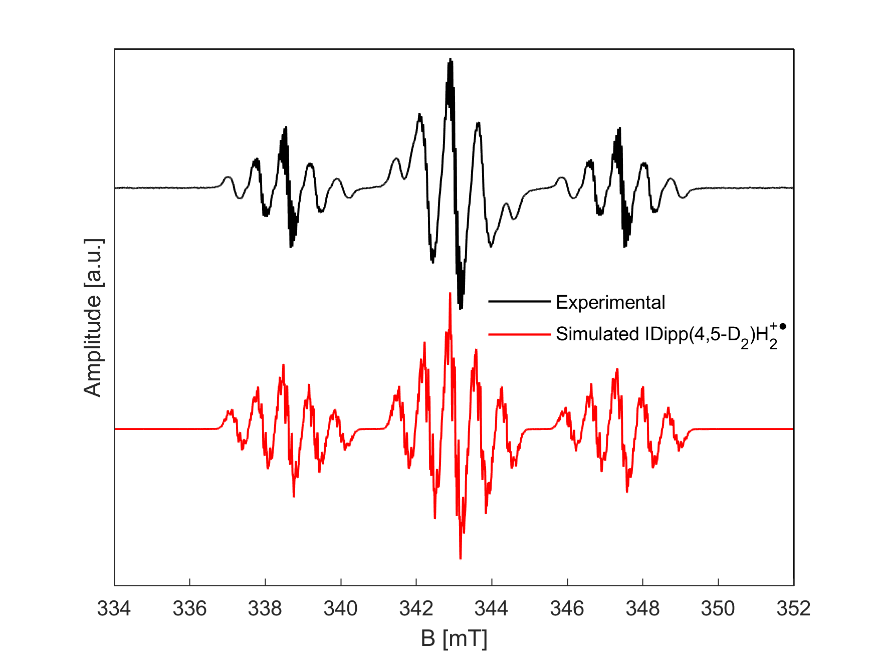


**Figure S49.** Room-temperature X-band EPR spectrum of an irradiated toluene solution of [IDipp(4,5-D_2_)H]BArF_24_ (0.15 M) and (*p-*MeOPh)_3_N (0.15 M), together with a simulation of [IDipp(4,5-D_2_)H_2_]^•+^. Simulation parameters: *g*_iso_ = 2.00383, 2×*A*^H^_iso_ = 123.9 MHz (C2-*H*), 2×*A*^N^_iso_ = 19.1 MHz, 2×*A*^D^_iso_ = 3.0 MHz (C4,5-*D*), 2×*A*^H^_iso_ = 1.0 MHz (*p-*Dipp*H*), 4×*A*^H^_iso_ = 2.5 MHz (*m*-Dipp*H*), 4×*A*^H^_iso_ = 0.9 MHz, (C*H*(CH_3_)_2_), lwpp = 0.015053 & 0.0111604 (Gaussian & Lorentzian). Microwave frequency = 9.6208 GHz; power = 0.3162 mW, modulation amplitude = 0.25 G.


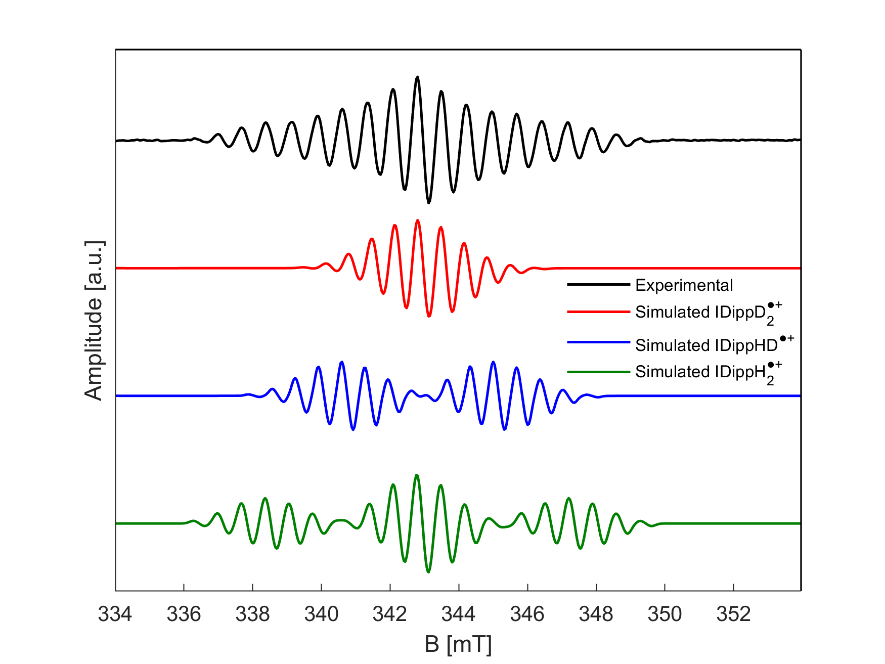


**Figure S50.** Room-temperature X-band EPR spectrum of an irradiated toluene solution of [IDippH]BArF_24_ (0.04 M), [IDippD]BArF_24_ (0.11 M) in a 1:3 ratio, and (*p-*MeOPh)_3_N (0.15 M), together with a simulation showing the presence of [IDippD_2_]^•+^, [IDippHD]^•+^, and [IDippH_2_]^•+^. Simulation parameters: [IDippD_2_]^•+^: *g*_iso_ = 2.0031, 2×*A*^D^_iso_ = 18.5 MHz (C2-*D*), 2×*A*^N^_iso_ = 18.8 MHz, 2×*A*^H^_iso_ = 20.1 MHz (C4,5-*H*), lwpp = 0.2225 & 0.0642 (Gaussian & Lorentzian); [IDippHD]^•+^: *g*_iso_ = 2.0037, *A*^D^_iso_ = 18.9 MHz (C2-*D*), *A*^H^_iso_ = 123.5 MHz (C2-*H*), 2×*A*^N^_iso_ = 18.7 MHz, 2×*A*^H^_iso_ = 20.1 MHz (C4,5-*H*), lwpp = 0.1391 & 0.0542 (Gaussian & Lorentzian); [IDippH_2_]^•+^: *g*_iso_ = 2.0037, 2×*A*^H^_iso_ = 123.5 MHz (C2-*H*), 2×*A*^N^_iso_ = 19.0 MHz, 2×*A*^H^_iso_ = 20.0 MHz (C4,5-*H*), lwpp = 0.2278 & 0.0238 (Gaussian & Lorentzian). Ratio [IDippD_2_]^•+^ : [IDippHD]^•+^ : [IDippH_2_]^•+^ = 1:1.1:1. Microwave frequency = 9.6185 GHz; power = 3.162 mW; modulation amplitude = 4 G.


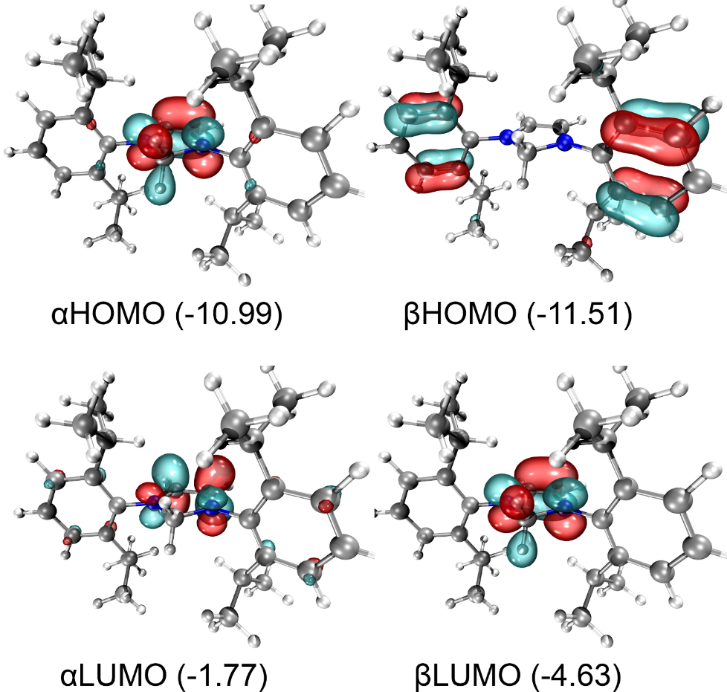


**Figure S51.** Frontier molecular orbitals of [IDippH_2_]^+•^ (isosurfaces 0.05 a.u.) and their corresponding energies (eV).


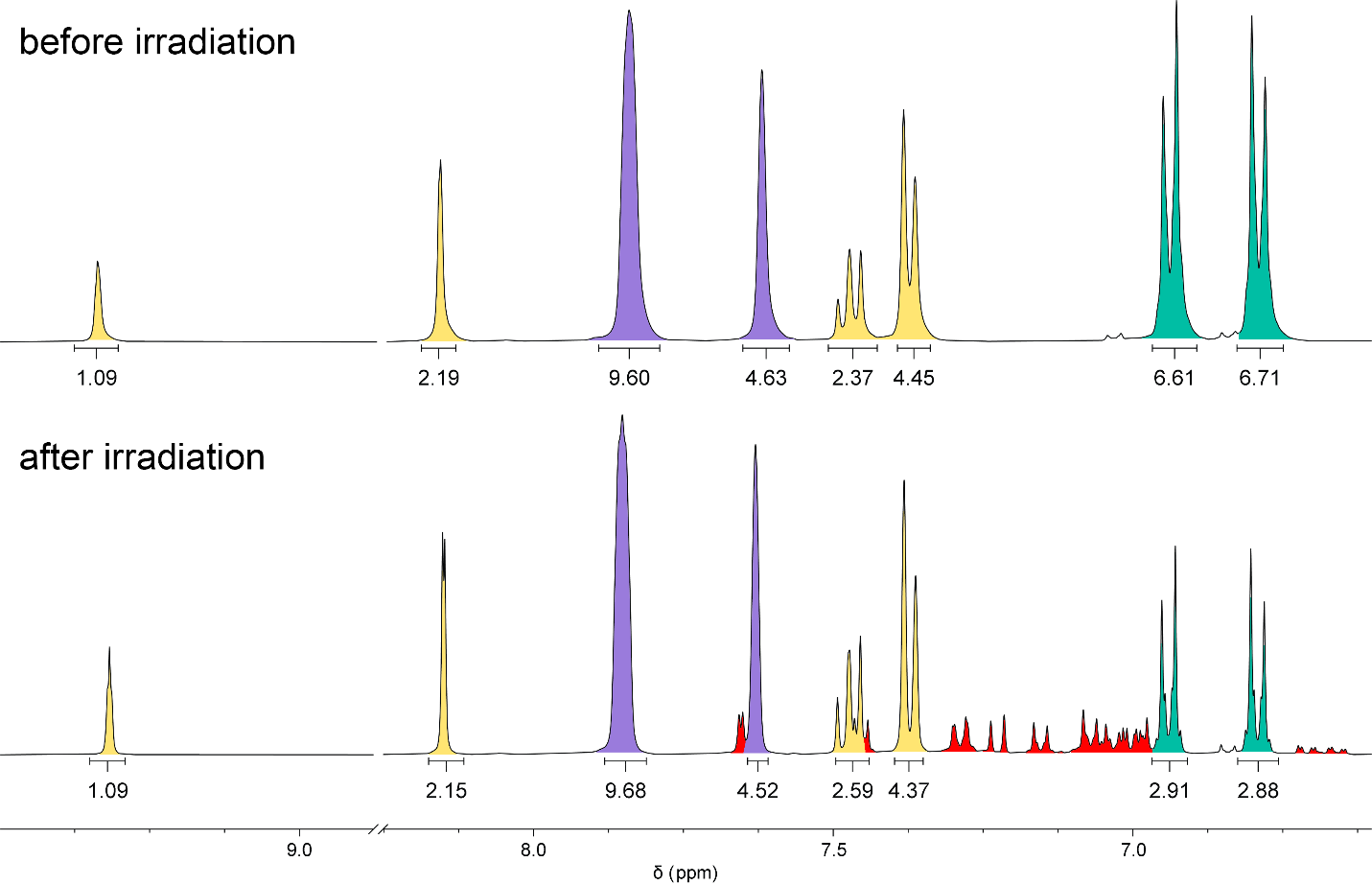


**Figure S52.** Aromatic region of ^1^H NMR spectra of a 0.15 M THF-d_8_ solution of [IXylH]BArF_24_ and (*p*-MeOPh)_3_N recorded before (top) and after (bottom) 12 h irradiation at 427 nm (NMR tube 6 cm from the light source). Integrals are normalized to the residual THF signal at 1.73 ppm. Color code: yellow, resonances of IXylH^+^; purple, BArF_24_^-^; teal, (*p*-MeOPh)_3_N red, (*p*-MeOPh)_3_N-derived decomposition products.


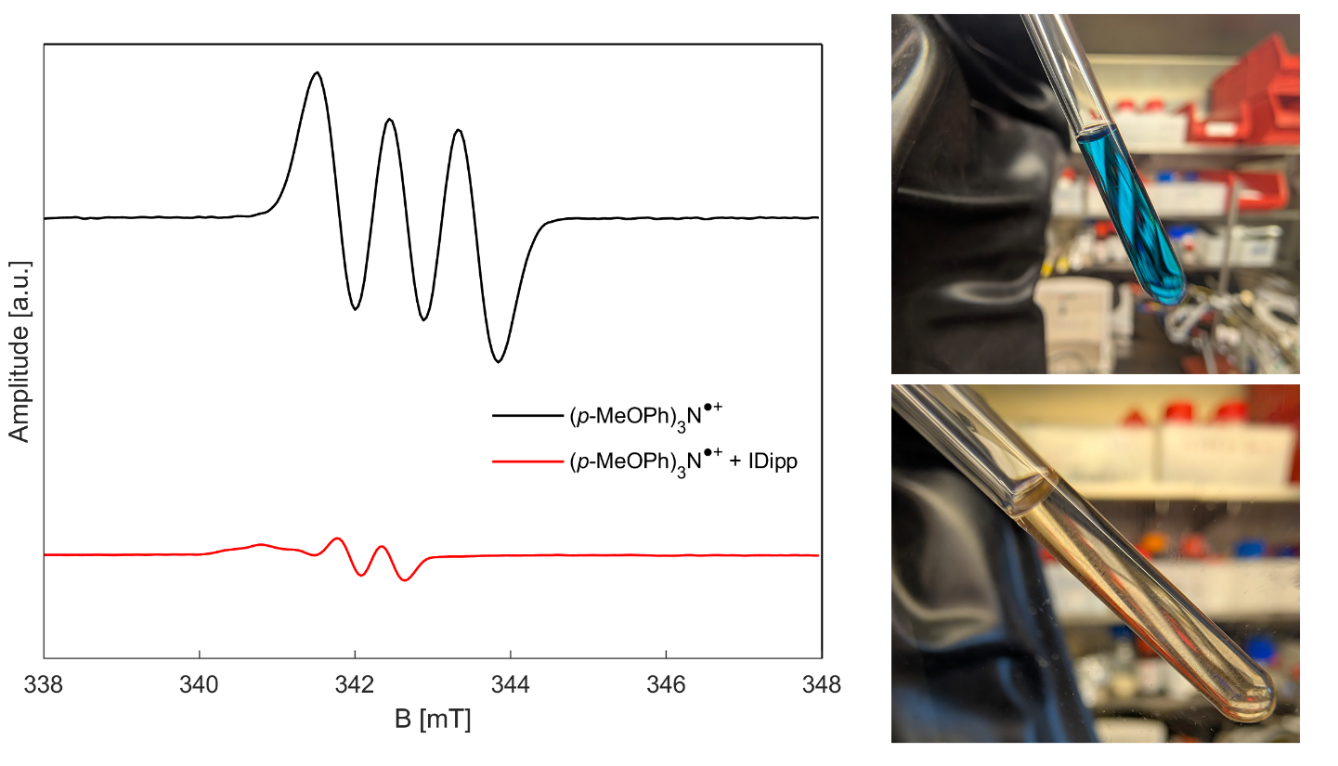


**Figure S53.** Room-temperature X-band EPR spectrum of [(*p*-MeOPh)_3_N]^•+^ in toluene (5 mM) generated by mixing (*p*-MeOPh)_3_N and NOPF_6_ (1:1 equiv.) before (top) and after (bottom) the addition of 1 equiv. of IDipp.

# III. Single-crystal X-ray Diffraction

X-ray diffraction data of **[IXylH]BArF_24_** and **[IDippH]BArF_24_** were measured on a Bruker D8 Quest Eco diffractometer using graphite-monochromated (Triumph) Mo-Kα radiation (λ = 0.71073 Å) and a CPAD Photon III C14 detector. The sample was cooled with N_2_ to 100 K with a Cryostream 700 (Oxford Cryosystems). Intensity data were integrated using the SAINT software.^[5]^ Absorption correction and scaling was executed with SADABS.^[6]^ The structures were solved using intrinsic phasing with the program SHELXT 2018/2^[7]^ against *F*^2^ of all reflections. Least-squares refinement was performed with SHELXL-2019/2.^[8]^ All non-hydrogen atoms were refined with anisotropic displacement parameters. The hydrogen atoms were introduced at calculated positions with a riding model. CheckCIF revealed no A- or B-level alerts. The X-ray crystallographic data for **[IXylH]BArF_24_** (2517460) and **[IDippH]BArF_24_** (2517461) were deposited at the Cambridge Crystallographic Data Centre (CCDC).

**Table S1.** Crystallographic data and structure refinement details for **[IXylH]BArF₂₄** and **[IDippH]BArF_24_**.

| Compound | [IXylH]BArF_24_ | [IDippH]BArF_24_ |
| --- | --- | --- |
| CCDC number | 2517460 | 2517461 |
| Empirical formula | C_51_H_33_BF_24_N_2_ | C_59_H_49_BF_24_N_2_ |
| Formula weight | 1140.60 | 1252.81 |
| Temperature [K] | 100(2) | 100(2) |
| Crystal system | monoclinic | monoclinic |
| Space group (number) | $P2_{1}/c$ (14) | $P2_{1}/c$ (14) |
| *a* [Å] | 17.3311(8) | 18.6588(9) |
| *b* [Å] | 16.6324(8) | 17.6535(9) |
| *c* [Å] | 16.7859(8) | 19.6283(14) |
| α [°] | 90 | 90 |
| β [°] | 91.537(2) | 118.262(2) |
| γ [°] | 90 | 90 |
| Volume [Å^3^] | 4836.9(4) | 5694.7(6) |
| *Z* | 4 | 4 |
| *ρ*_calc_ [gcm^−3^] | 1.566 | 1.461 |
| *μ* [mm^−1^] | 0.156 | 0.140 |
| *F*(000) | 2296 | 2552 |
| Crystal size [mm^3^] | 0.504×0.189×0.066 | 0.486×0.267×0.114 |
| Crystal colour | colourless | colourless |
| Crystal shape | plate | plate |
| Radiation | Mo*K_α_* (λ=0.71073 Å) | Mo*K_α_* (λ=0.71073 Å) |
| 2θ range [°] | 4.14 to 54.94 (0.77 Å) | 4.61 to 66.51 (0.65 Å) |
| Index ranges | −22 ≤ h ≤ 22 −21 ≤ k ≤ 21 −21 ≤ l ≤ 21 | −28 ≤ h ≤ 28 −27 ≤ k ≤ 27 −30 ≤ l ≤ 30 |
| Reflections collected | 201782 | 434774 |
| Independent reflections | 11065 *R*_int_ = 0.0540 *R*_sigma_ = 0.0187 | 21876 *R*_int_ = 0.0861 *R*_sigma_ = 0.0318 |
| Completeness to θ = 25.242° | 99.9 % | 99.9 % |
| Data / Restraints / Parameters | 11065/0/735 | 21876/768/838 |
| Goodness-of-fit on *F*^2^ | 1.038 | 1.029 |
| Final *R* indexes [*I*≥2σ(*I*)] | *R*_1_ = 0.0390, w*R*_2_ = 0.0937 | *R*_1_ = 0.0511, w*R*_2_ = 0.1252 |
| Final *R* indexes [all data] | *R*_1_ = 0.0517, w*R*_2_ = 0.1027 | *R*_1_ = 0.0837, w*R*_2_ = 0.1529 |
| Largest peak/hole [eÅ^−3^] | 0.61/-0.36 | 0.55/-0.38 |


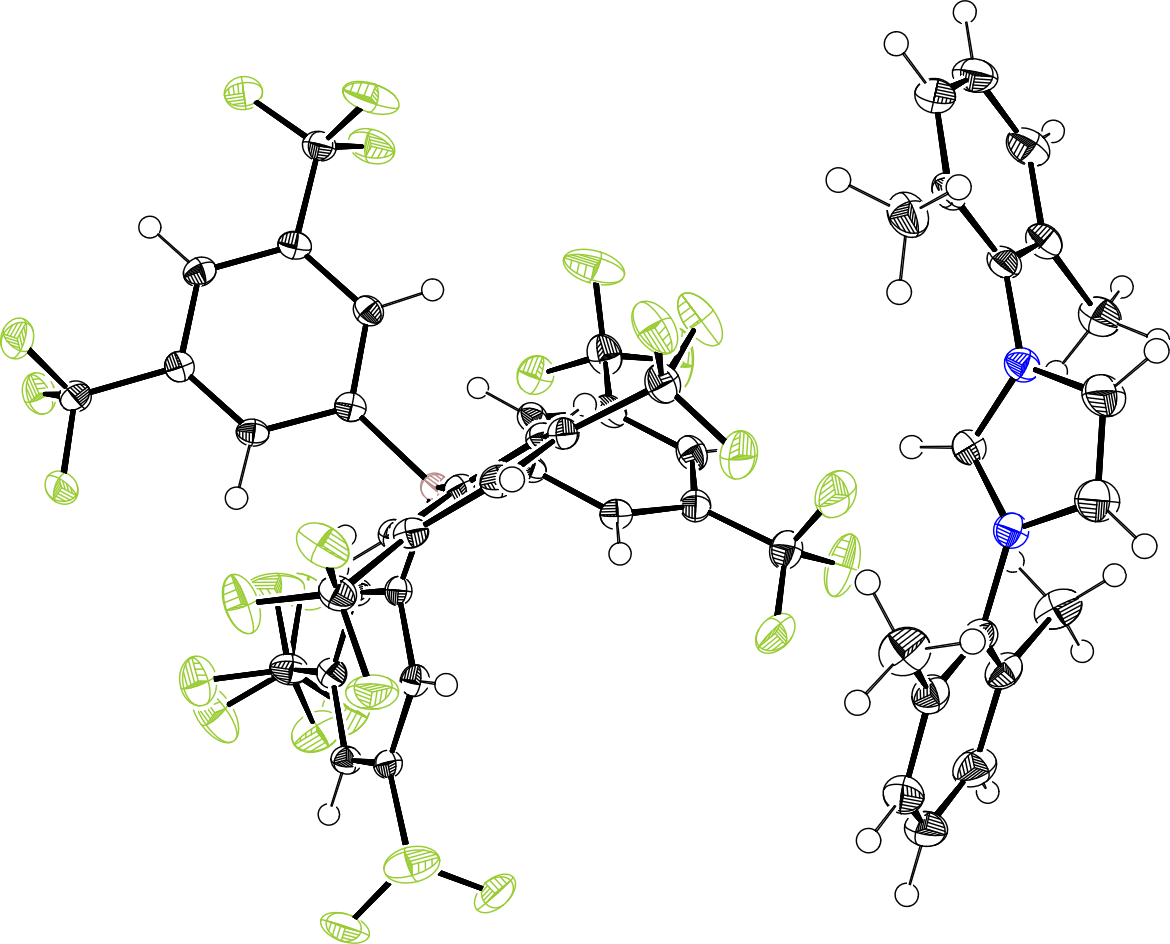


**Figure S54**. Molecular structure of [IXylH]BArF_24_ in the solid-state. Displacement ellipsoids are drawn at 50% probability level.


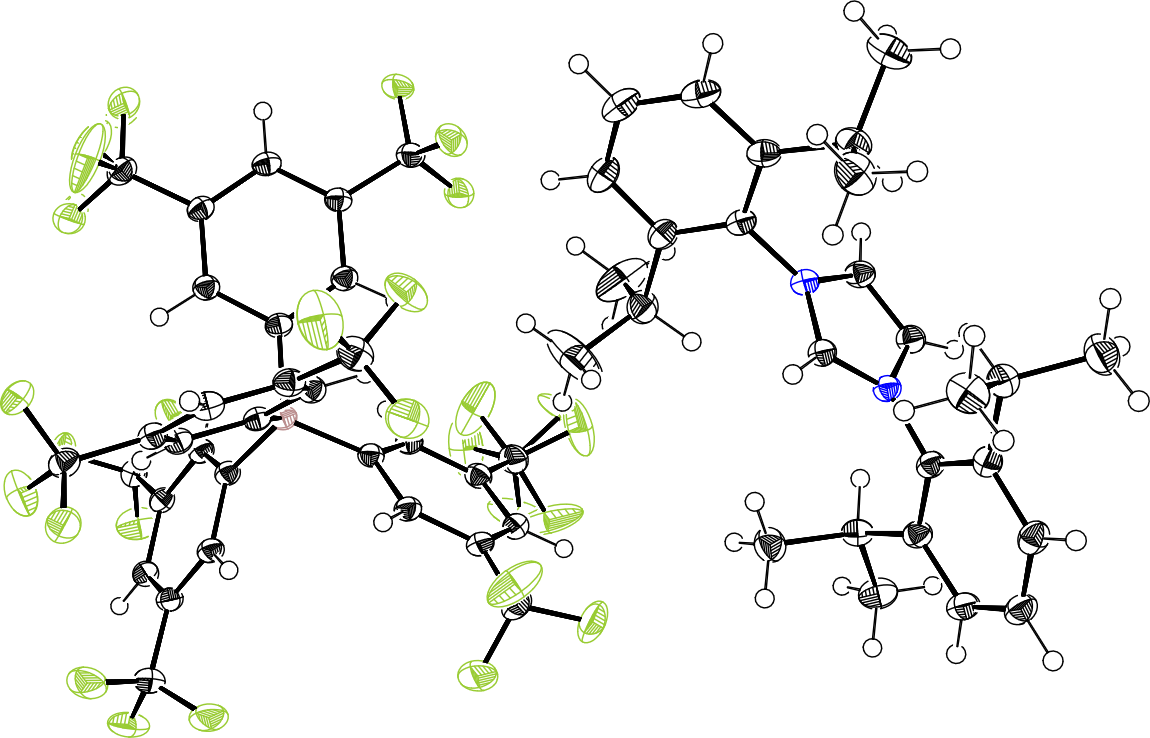


**Figure S55**. Molecular structure of [IDippH]BArF_24_ in the solid-state. Displacement ellipsoids are drawn at 50% probability level.

# IV. DFT calculations

## General information

All geometry optimizations were calculated using the (U)*ω*B97X-D density functional^[9]^ and the 6-31G(d)^[10,11]^ basis set as implemented in Gaussian 16 (Revision C.01)^[12]^ without symmetry constraints. The obtained geometries were characterized as true minima having no imaginary frequency via harmonic frequency calculation. Connectivities between minima and transition states were validated either by intrinsic reaction coordinate (IRC) calculations.^[13]^ Single-point calculations on the optimized structures were performed using (U)*ω*B97X-D and the 6-311+G(d,p)^[9]^ basis set taking solvents effects (toluene) into account by means of the self-consistent reaction field (SCRF) method using the polarizable continuum model (PCM).^[14]^ Time-dependent DFT (TD-DFT) calculations (with 20 states modelled) were carried out at the ωB97X-D/6-311++G(d,p) level with PCM(toluene). EPR parameters (g-tensors and isotropic hyperfine coupling constants) were computed in ORCA 6.1.1 at the ωB97X-D3/IGLO-II level of theory. The energy diagrams were created in Energy Diagram Plotter (CDXML).^[15]^ Natural bond orbital (NBO) analyses were performed with the NBO 7.0 program interfaced to Gaussian^[16]^. Molecular orbitals surfaces were created in Multiwfn^[17]^ and rendered in VMD^[18]^, molecular structures were rendered in ChemCraft.^[19]^

## Natural bond analysis


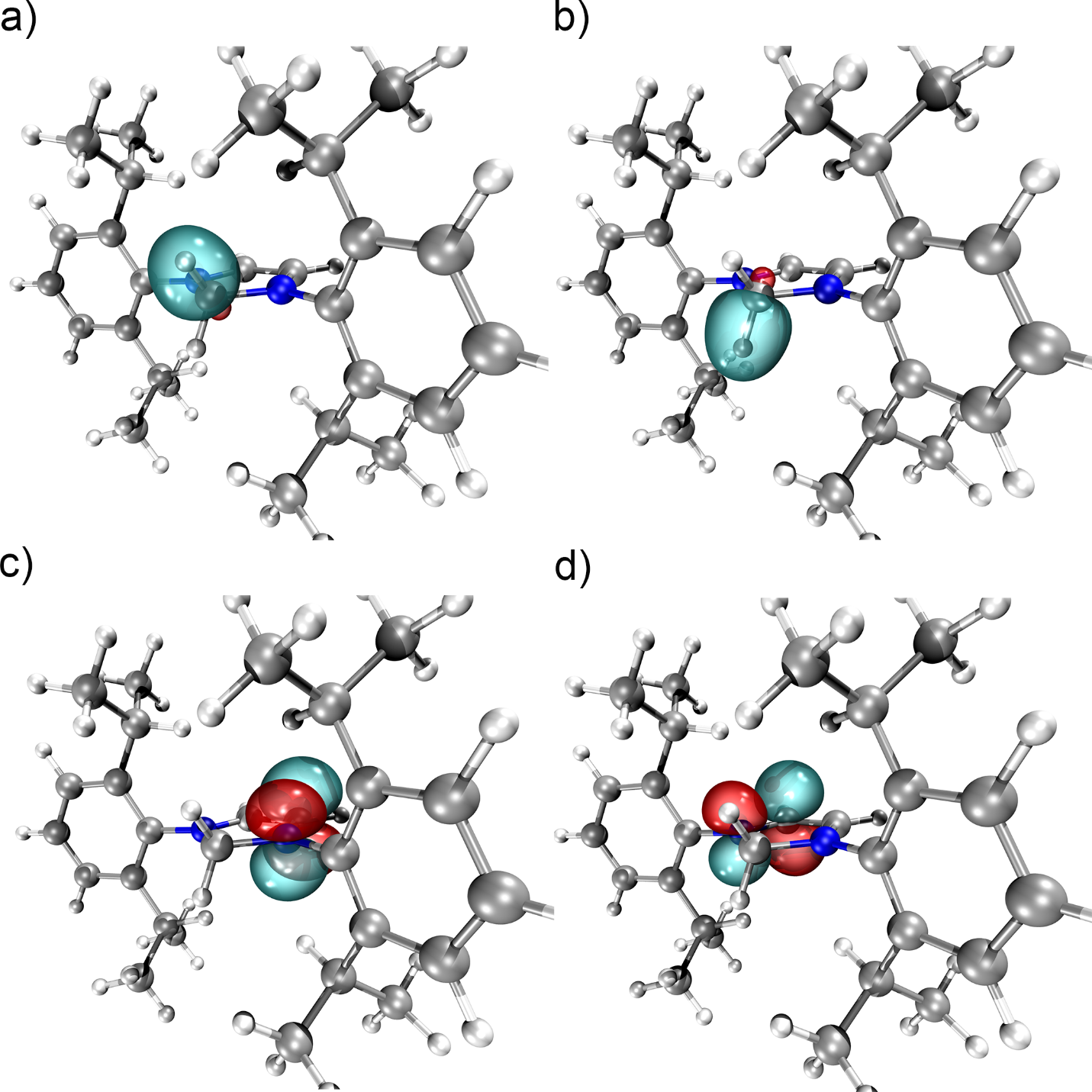


**Figure S56**. Selected beta-spin NBOs illustrating the hyperconjugative interaction in [IDippH_2_]^•+^ at the U*ω*B97X-D/6-31G* level. Shown are the two donor σ(C-H) orbitals of the methylene unit, BD(1) C1-H22 (a) and BD(1) C1-H67 (b), and the two acceptor π* orbitals of the adjacent N-C-C-N fragment, BD*(2) N2-C3 (c) and BD*(2) C4-N5 (d). Second-order perturbation analysis gives E(2) = 2.57 kcal mol^-1^ for each σ(C-H)→π* interaction. No corresponding σ(C-H)→π* interaction is found in the alpha-spin orbitals.

## Transition state analysis


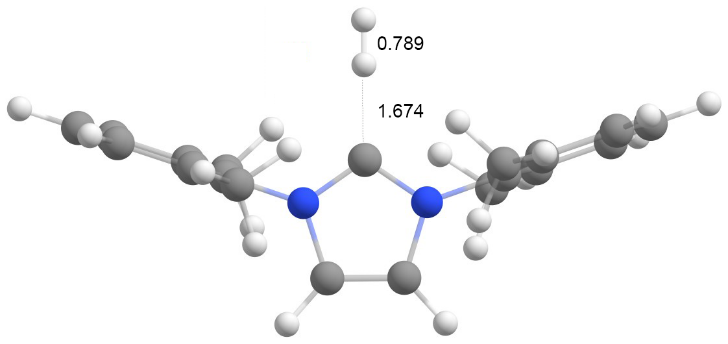


**Figure S57.** Transition state of the reaction of H_2_ addition to [IXyl]^•+^, akin to the transition states reported by Bertrand et al for H_2_ splitting on carbenes^[20]^.

**Figure S58.** Computed Gibbs free energy profile (Δ*G*, kcal mol^-1^) for activation of H_2_ by the imidazolium-derived radical cation IXyl^•+^ in toluene at the SCRF(Toluene)/(U)ωB97X-D/6-311+G(d,p)//(U)ωB97X-D/6-31G(d) level of theory. The reactant encounter complex **[IXyl^•+^,H_2_]** is taken as reference (ΔG = 0.0); cleavage of the H–H bond proceeds via **TS [IXyl^•+^···H···H]^‡^** (ΔG^‡^ = +1.4) to afford the product encounter complex **[IXylH^+^,H^•^]** (ΔG = −24.1), consistent with H-atom abstraction from H_2_ by [IXyl]^•+^.

## Calculated and experimental EPR parameters

**Tables S1–S5.** Summary of experimental and calculated EPR parameters for imidazolium-derived radicals and the triarylamine radical cation investigated in this work. Isotropic parameters (*g*_iso_ and *A*_iso_) are reported for the σ-complex radical cations ([IDippH_2_]^•+^ and [IXylH_2_]^•+^), whereas anisotropic parameters are given as principal values of the *g*- and *A*-tensors for [IXylH]^•^, [IDippH]^•^ and [(*p*-MeOPh)_3_N]^•+^. Hyperfine couplings are listed in MHz. Experimental values were obtained by simulation of X-band EPR spectra, and calculated values were obtained as described in the Computational Details.

**Table S2**. Calculated and experimental isotropic EPR parameters for [IDippH_2_]^•+^.

| [IDippH_2_]^•+^ |  | Calculated | Experimental |
| --- | --- | --- | --- |
|  | *g* | 2.00321 | 2.00377 |
|  | $A_{iso}$ | | |
|  | $H_{CH_{2}}$ | 119.84 (^1^H) /  18.44 (D) | 123.5 (^1^H) /  18.9 (D) |
|  | $N$ | 16.70 | 18.9 |
|  | $H_{C_{4-5}}$ | –15.19 (1H) /  –2.33 (D) | 20.3 (^1^H) /  3.0 (D) |
|  | $H_{p}$ | –0.09 | 0.9 |
|  | $H_{m}$ | 1.06 | 2.5 |
|  | $H_{\mathrm{CH}}$ | 0.14 | 0.9 |
|  | $H_{CH_{3}}$ | 0.09 | - |

**Table S3**. Calculated and experimental isotropic EPR parameters for [IXylH_2_]^•+^.

| IXylH_2_^•+^ |  | Calculated | Experimental |
| --- | --- | --- | --- |
|  | *g* | 2.00293 | 2.00294 |
|  | $A_{iso}$ | | |
|  | $H_{CH_{2}}$ | 116.58 (^1^H) /  17.94 (D) | 123.7 (^1^H) /  18.9 (D) |
|  | $N$ | 16.49 | 18.9 |
|  | $H_{C_{4-5}}$ | –15.48 | 20.6 |
|  | $H_{p}$ | –0.30 | 1.0 |
|  | $H_{m}$ | 1.16 | 2.5 |
|  | $H_{CH_{3}}$ | 0.04 | 0.5 |

**Table S4**. Calculated and experimental anisotropic EPR parameters for [IDippH]^•^.

| IDippH^•^ |  | Calculated | Experimental |
| --- | --- | --- | --- |
|  | *g* | 2.001988, 2.003223, 2.003510 | 2.0035, 2.0023, 2.0035 |
|  | *A* | | |
|  | $H_{C_{2}}$ | 68.1, 85.6, 116.0 | 86, 93, 113 |
|  | $N$ | -3.1, -3.4, 29.1 | <5, <5, 29 |
|  | $H_{C_{4-5}}$ | -2.2, 0.3, 0.0 | - |
|  | $H_{p}$ | 0.4, -0.9, -1.2 | - |
|  | $H_{m}$ | 0.1, 0.2, 1.7 | - |
|  | $H_{\mathrm{CH}}$ | -3.8, 0.5, 1.9 | - |
|  | $H_{CH_{3}}$ | -0.6, -0.7, 1.8 | - |

**Table S5**. Calculated and experimental anisotropic EPR parameters for [IXylH]^•^.

| IXylH^•^ |  | Calculated | Experimental |
| --- | --- | --- | --- |
|  | *g* | 2.002036, 2.003226, 2.003532 | 2.0041, 2.0031, 2.0041 |
|  | *A* | | |
|  | $H_{C_{2}}$ | 70.9, 88.7, 118.9 | 67 88 108 |
|  | $N$ | -1.8, -2.2, 28.3 | <5, <5, 26 |
|  | $H_{C_{4-5}}$ | 2.3, -3.2, -4.0 | - |
|  | $H_{p}$ | -0.1, -1.9, -2.8 | - |
|  | $H_{m}$ | 0.5, 0.7, 2.2 | - |
|  | $H_{CH_{3}}$ | -0.4, -0.7, 4.9 | - |

**Table S6**. Calculated and experimental anisotropic EPR parameters for [(*p*-MeOPh)_3_N]^•+^.

| [(*p*-MeOPh)_3_N]^•+^ |  | Calculated | Experimental |
| --- | --- | --- | --- |
|  | *g* | 2.002681, 2.003665, 2.003665 | 2.0046, 2.0046, 2.0025 |
|  | *A* | | |
|  | $N$ | 0.3, 0.3, 51.1 | <5, <5, 61 |
|  | $H_{o}$ | -1.2, -5.5, -6.5 | - |
|  | $H_{m}$ | 0.4, 2.0, 2.5 | - |
|  | $H_{CH_{3}}$ | 2.2, 2.4, 4.3 | - |

## TD-DFT Analysis of Excited States

**Table S7.** Calculated vertical excitation energies (eV), wavelengths (nm), oscillator strengths (F_osc_), and main configurations for the first 20 singlet excited states of [IXylH^+^, (*p*-MeOPh)_3_N]. Only transitions with a contribution >10% are shown.

| **State** | **Energy (eV)** | **Wavelength (nm)** | **F_osc._** | **Major Transitions (>10%)** |
| --- | --- | --- | --- | --- |
| S1 | 3.788 | 327.31 | 0.0035 | H → L (93.1%) |
| S2 | 4.241 | 292.34 | 0.0273 | H → L+1 (12.9%), H → L+2 (22.2%), H → L+5 (28.0%), H → L+6 (22.7%) |
| S3 | 4.637 | 267.4 | 0.0686 | H → L+1 (20.0%), H → L+7 (40.1%) |
| S4 | 4.737 | 261.75 | 0.0352 | H → L+1 (33.8%), H → L+2 (29.1%), H → L+8 (19.2%) |
| S5 | 4.768 | 260.02 | 0.0997 | H → L+1 (26.5%), H → L+6 (15.0%), H → L+7 (27.5%) |
| S6 | 4.907 | 252.68 | 0.1644 | H → L+8 (34.8%), H → L+10 (15.2%) |
| S7 | 4.975 | 249.23 | 0.1444 | H → L+2 (12.5%), H → L+8 (18.7%), H → L+9 (41.3%) |
| S8 | 5.029 | 246.53 | 0.1396 | H → L+2 (11.7%), H → L+9 (18.2%), H → L+10 (37.6%) |
| S9 | 5.102 | 243.02 | 0.0416 | H → L+4 (50.5%), H → L+7 (12.5%), H → L+9 (13.1%) |
| S10 | 5.202 | 238.33 | 0.0101 | H → L+3 (71.2%), H → L+4 (13.8%) |
| S11 | 5.382 | 230.36 | 0.0231 | H-8 → L+4 (14.6%), H-6 → L (16.1%), H-6 → L+2 (23.1%) |
| S12 | 5.43 | 228.35 | 0.0262 | H-9 → L+3 (13.1%), H-7 → L+1 (42.3%) |
| S13 | 5.448 | 227.57 | 0.0188 | H-2 → L (41.1%), H-1 → L (38.5%) |
| S14 | 5.575 | 222.38 | 0.0019 | H → L+5 (44.2%), H → L+6 (40.4%) |
| S15 | 5.71 | 217.14 | 0.0012 | H-2 → L (27.4%), H-1 → L (39.7%) |
| S16 | 5.828 | 212.75 | 0.0041 | H-1 → L+5 (10.0%), H-1 → L+10 (10.5%), H → L+10 (13.7%) |
| S17 | 5.903 | 210.05 | 0.0035 | H-8 → L (12.2%), H → L+10 (10.8%) |
| S18 | 5.928 | 209.15 | 0.0089 | H-8 → L (38.2%) |
| S19 | 5.985 | 207.15 | 0.009 | H-8 → L (17.7%), H-4 → L (14.3%), H → L+11 (13.1%) |
| S20 | 6.014 | 206.16 | 0.0042 | H-1 → L+1 (68.9%), H-1 → L+2 (10.3%) |

**Table S8.** Calculated vertical excitation energies (eV), wavelengths (nm), oscillator strengths (F_osc_), and main configurations for the first 20 singlet excited states of [IDippH^+^, (*p*-MeOPh)_3_N]. Only transitions with a contribution >10% are shown.

| **State** | **Energy (eV)** | **Wavelength (nm)** | **F_osc._** | **Major Transitions (>10%)** |
| --- | --- | --- | --- | --- |
| S1 | 3.7376 | 331.72 | 0.0039 | H → L (95.8%) |
| S2 | 4.2091 | 294.56 | 0.0354 | H → L+4 (28.3%), H → L+5 (39.6%) |
| S3 | 4.6248 | 268.09 | 0.0183 | H → L+2 (13.4%), H → L+7 (29.4%), H → L+8 (14.7%), H → L+10 (11.6%) |
| S4 | 4.6641 | 265.83 | 0.1476 | H → L+1 (17.1%), H → L+7 (42.1%), H → L+8 (15.7%) |
| S5 | 4.7344 | 261.88 | 0.0218 | H → L+1 (37.0%), H → L+3 (18.2%) |
| S6 | 4.8079 | 257.88 | 0.1767 | H → L+1 (11.1%), H → L+3 (16.2%), H → L+8 (38.5%), H → L+10 (10.6%) |
| S7 | 4.8404 | 256.14 | 0.1001 | H → L+9 (34.4%), H → L+10 (11.9%) |
| S8 | 4.9143 | 252.29 | 0.1407 | H → L+3 (39.0%), H → L+8 (12.9%), H → L+9 (16.8%) |
| S9 | 5.0285 | 246.56 | 0.0432 | H → L+2 (32.7%), H → L+10 (21.5%) |
| S10 | 5.1127 | 242.5 | 0.0236 | H → L+2 (12.5%), H → L+4 (42.6%), H → L+5 (13.3%) |
| S11 | 5.3821 | 230.36 | 0.0006 | H-7 → L+3 (10.4%), H-6 → L+1 (19.9%) |
| S12 | 5.3956 | 229.79 | 0.0393 | H-6 → L+1 (13.9%), H-6 → L+3 (10.2%) |
| S13 | 5.4801 | 226.25 | 0.0016 | H-2 → L (14.1%), H-1 → L (48.1%), H → L+6 (16.8%) |
| S14 | 5.6094 | 221.03 | 0.0088 | H-2 → L (63.5%), H-1 → L (18.4%) |
| S15 | 5.634 | 220.06 | 0.0007 | H-1 → L (21.5%), H → L+5 (15.1%), H → L+6 (49.5%) |
| S16 | 5.772 | 214.8 | 0.0022 | H-4 → L (25.1%), H-3 → L (37.2%) |
| S17 | 5.8672 | 211.32 | 0.0006 | H-2 → L+5 (19.9%), H → L+9 (11.5%) |
| S18 | 5.9408 | 208.7 | 0.0084 | H-8 → L (10.3%), H-6 → L (20.9%), H-4 → L (16.1%), H-3 → L (10.4%) |
| S19 | 5.9501 | 208.37 | 0.0018 | H-8 → L (41.8%), H-6 → L (18.8%) |
| S20 | 5.9664 | 207.81 | 0.0031 | H-8 → L (10.8%) |

## Coordinates of optimized structures

The following optimized geometries are provided as Cartesian coordinates (Å). $E^{\circ}$ is the electronic energy of the optimized structure, $H^{\circ}$ is the sum of electronic and thermal enthalpies, and $G^{\circ}$ is the sum of electronic and thermal free energies. All energies are given in Hartree.

**IXylH^+^**

E° = -845.94518944 *E_h_*

G° = -845.63478044 *E_h_*

H° = -845.56323544 *E_h_*


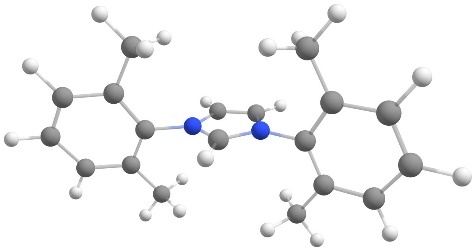


| C | 0.000000 | 0.000000 | 0.266176 |
| --- | --- | --- | --- |
| N | 1.083926 | -0.004344 | -0.508900 |
| C | 0.679608 | -0.002763 | -1.828855 |
| C | -0.679607 | 0.002832 | -1.828855 |
| N | -1.083926 | 0.004370 | -0.508900 |
| C | -2.449655 | 0.012400 | -0.035035 |
| C | 2.449655 | -0.012396 | -0.035034 |
| C | -3.096628 | -1.218234 | 0.115299 |
| C | -4.415708 | -1.183869 | 0.566030 |
| C | -5.040753 | 0.026978 | 0.845657 |
| C | -4.362602 | 1.230258 | 0.682648 |
| C | -3.041874 | 1.249876 | 0.235083 |
| C | 3.041857 | -1.249883 | 0.235070 |
| C | 4.362585 | -1.230288 | 0.682636 |
| C | 5.040752 | -0.027019 | 0.845659 |
| C | 4.415723 | 1.183840 | 0.566046 |
| C | 3.096644 | 1.218228 | 0.115313 |
| C | 2.400580 | 2.519213 | -0.194282 |
| C | 2.286296 | -2.543201 | 0.062384 |
| C | -2.400545 | -2.519207 | -0.194304 |
| C | -2.286330 | 2.543205 | 0.062412 |
| H | 0.000000 | -0.000017 | 1.345392 |
| H | 1.396231 | -0.006904 | -2.634816 |
| H | -1.396230 | 0.006998 | -2.634816 |
| H | -4.955788 | -2.116305 | 0.697341 |
| H | -6.068516 | 0.032802 | 1.193748 |
| H | -4.861812 | 2.168667 | 0.903116 |
| H | 4.861783 | -2.168705 | 0.903094 |
| H | 6.068514 | -0.032859 | 1.193752 |
| H | 4.955815 | 2.116267 | 0.697368 |
| H | 3.058394 | 3.365894 | 0.012186 |
| H | 1.495126 | 2.648711 | 0.410186 |
| H | 2.103114 | 2.579331 | -1.247269 |
| H | 1.826695 | -2.619893 | -0.929080 |
| H | 1.486361 | -2.643036 | 0.806064 |
| H | 2.954452 | -3.397984 | 0.185964 |
| H | -1.495122 | -2.648722 | 0.410210 |
| H | -2.103022 | -2.579292 | -1.247276 |
| H | -3.058366 | -3.365897 | 0.012103 |
| H | -2.954491 | 3.397979 | 0.186029 |
| H | -1.826752 | 2.619927 | -0.929061 |
| H | -1.486377 | 2.643029 | 0.806075 |

**[IXylH]^•^**

E° = -846.04476286 *E_h_*

G° = -845.73715886 *E_h_*

H° = -845.66637086 *E_h_*


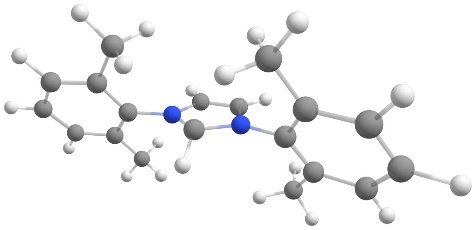


| C | 0.000001 | 0.200644 | 0.358894 |
| --- | --- | --- | --- |
| N | 1.112222 | -0.269983 | -0.376925 |
| C | 0.673243 | -0.905862 | -1.535022 |
| C | -0.673244 | -0.905860 | -1.535022 |
| N | -1.112222 | -0.269981 | -0.376925 |
| C | -2.461759 | -0.024178 | -0.031153 |
| C | 2.461760 | -0.024182 | -0.031154 |
| C | -3.297702 | -1.116947 | 0.254477 |
| C | -4.628741 | -0.866039 | 0.588193 |
| C | -5.113556 | 0.434349 | 0.658411 |
| C | -4.269006 | 1.504438 | 0.383554 |
| C | -2.937078 | 1.297836 | 0.026471 |
| C | 3.297697 | -1.116950 | 0.254496 |
| C | 4.628736 | -0.866043 | 0.588213 |
| C | 5.113556 | 0.434345 | 0.658412 |
| C | 4.269011 | 1.504433 | 0.383533 |
| C | 2.937084 | 1.297831 | 0.026449 |
| C | 2.043182 | 2.460290 | -0.315538 |
| C | 2.766067 | -2.527769 | 0.224815 |
| C | -2.766079 | -2.527768 | 0.224778 |
| C | -2.043170 | 2.460299 | -0.315487 |
| H | 0.000001 | 0.004919 | 1.435974 |
| H | 1.369453 | -1.260240 | -2.279037 |
| H | -1.369454 | -1.260236 | -2.279037 |
| H | -5.284920 | -1.702961 | 0.813181 |
| H | -6.149759 | 0.614927 | 0.928811 |
| H | -4.650761 | 2.521146 | 0.430217 |
| H | 5.284910 | -1.702964 | 0.813217 |
| H | 6.149758 | 0.614923 | 0.928813 |
| H | 4.650772 | 2.521141 | 0.430181 |
| H | 2.624497 | 3.383059 | -0.401297 |
| H | 1.265778 | 2.601316 | 0.442628 |
| H | 1.520718 | 2.286670 | -1.262436 |
| H | 2.654966 | -2.903141 | -0.799433 |
| H | 1.778078 | -2.586205 | 0.692584 |
| H | 3.442472 | -3.205769 | 0.753277 |
| H | -1.778089 | -2.586214 | 0.692542 |
| H | -2.654986 | -2.903131 | -0.799474 |
| H | -3.442486 | -3.205770 | 0.753236 |
| H | -2.624485 | 3.383068 | -0.401251 |
| H | -1.520682 | 2.286690 | -1.262374 |
| H | -1.265785 | 2.601322 | 0.442699 |

**(*p*-MeOPh)_3_N**

E° = -1093.18224733 *E_h_*

G° = -1092.85444633 *E_h_*

H° = -1092.77695733 *E_h_*


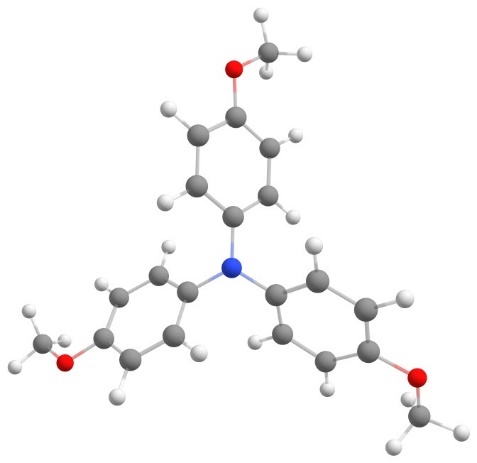


| C | 6.269738 | -1.130113 | -0.667220 |
| --- | --- | --- | --- |
| O | 5.580645 | -0.198500 | 0.131400 |
| C | 4.220922 | -0.212926 | 0.091760 |
| C | 3.455246 | -1.086999 | -0.678310 |
| C | 2.063651 | -1.002789 | -0.649900 |
| C | 1.414397 | -0.073403 | 0.160650 |
| N | 0.001490 | -0.000490 | 0.205770 |
| C | -0.768672 | -1.187442 | 0.160280 |
| C | -0.414361 | -2.293845 | 0.945720 |
| C | -1.158919 | -3.459082 | 0.902500 |
| C | -2.296045 | -3.545808 | 0.091840 |
| O | -2.965817 | -4.729108 | 0.132780 |
| C | -4.115631 | -4.859912 | -0.668110 |
| C | -2.664838 | -2.447662 | -0.683690 |
| C | -1.894410 | -1.285800 | -0.655310 |
| C | -0.642214 | 1.259546 | 0.160940 |
| C | -0.165460 | 2.285120 | -0.653340 |
| C | -0.788756 | 3.532172 | -0.681380 |
| C | -1.925506 | 3.759236 | 0.092900 |
| O | -2.618006 | 4.929430 | 0.133350 |
| C | -2.159196 | 5.991008 | -0.668610 |
| C | -2.417700 | 2.729714 | 0.902600 |
| C | -1.778600 | 1.503489 | 0.945560 |
| C | 2.194573 | 0.791897 | 0.941190 |
| C | 3.576022 | 0.731710 | 0.897680 |
| H | 7.331760 | -0.953503 | -0.491020 |
| H | 6.052414 | -0.987354 | -1.734340 |
| H | 6.024383 | -2.163299 | -0.386690 |
| H | 3.920633 | -1.829662 | -1.316410 |
| H | 1.475580 | -1.679294 | -1.262190 |
| H | 0.458381 | -2.232112 | 1.588320 |
| H | -0.886033 | -4.317941 | 1.507000 |
| H | -3.880441 | -4.748794 | -1.735200 |
| H | -4.497341 | -5.865933 | -0.488250 |
| H | -4.886049 | -4.127144 | -0.392690 |
| H | -3.537754 | -2.480215 | -1.325770 |
| H | -2.182008 | -0.439608 | -1.271440 |
| H | 0.712109 | 2.113223 | -1.268710 |
| H | -0.381028 | 4.305539 | -1.322440 |
| H | -2.842827 | 6.822420 | -0.491020 |
| H | -1.140851 | 6.295975 | -0.392390 |
| H | -2.178193 | 5.729911 | -1.735290 |
| H | -3.298946 | 2.920692 | 1.506280 |
| H | -2.160781 | 0.715598 | 1.587110 |
| H | 1.704033 | 1.519745 | 1.579950 |
| H | 4.182502 | 1.401921 | 1.498120 |

**[(*p*-MeOPh)_3_N]^•+^**

E° = -1092.98515262 *E_h_*

G° = -1092.65490362 *E_h_*

H° = -1092.57894962 *E_h_*


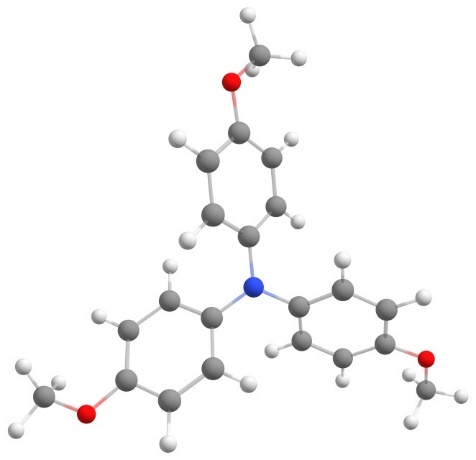


| C | 4.383945 | -4.636337 | -0.552620 |
| --- | --- | --- | --- |
| O | 4.351550 | -3.410738 | 0.166530 |
| C | 3.264619 | -2.641506 | 0.097550 |
| C | 2.119519 | -2.938976 | -0.659180 |
| C | 1.045971 | -2.065541 | -0.654470 |
| C | 1.093080 | -0.882195 | 0.097590 |
| N | -0.000050 | -0.000169 | 0.097120 |
| C | -1.310586 | -0.505702 | 0.097750 |
| C | -1.629686 | -1.650563 | 0.855570 |
| C | -2.915057 | -2.139037 | 0.855740 |
| C | -3.920146 | -1.506052 | 0.097880 |
| O | -5.129935 | -2.062411 | 0.167020 |
| C | -6.207144 | -1.478189 | -0.553130 |
| C | -3.604806 | -0.366314 | -0.659670 |
| C | -2.311497 | 0.126414 | -0.655030 |
| C | 0.217346 | 1.387589 | 0.097750 |
| C | 1.264734 | 1.938480 | -0.655580 |
| C | 1.484626 | 3.304894 | -0.660150 |
| C | 0.655717 | 4.147739 | 0.098040 |
| O | 0.778806 | 5.473621 | 0.167300 |
| C | 1.822898 | 6.114529 | -0.553400 |
| C | -0.394538 | 3.593654 | 0.856430 |
| C | -0.614162 | 2.236240 | 0.856170 |
| C | 2.244518 | -0.585407 | 0.854550 |
| C | 3.310406 | -1.454087 | 0.854650 |
| H | 5.352519 | -5.081077 | -0.330140 |
| H | 4.300545 | -4.460296 | -1.630630 |
| H | 3.586101 | -5.308108 | -0.217730 |
| H | 2.065854 | -3.836919 | -1.262400 |
| H | 0.172243 | -2.284296 | -1.259350 |
| H | -0.866438 | -2.126753 | 1.461770 |
| H | -3.185449 | -3.005987 | 1.447910 |
| H | -6.012548 | -1.495017 | -1.631040 |
| H | -7.076754 | -2.094313 | -0.330420 |
| H | -6.390045 | -0.451003 | -0.219300 |
| H | -4.355386 | 0.128821 | -1.263460 |
| H | -2.063718 | 0.991890 | -1.260570 |
| H | 1.890073 | 1.291331 | -1.261610 |
| H | 2.288320 | 3.707472 | -1.264350 |
| H | 1.724146 | 7.175683 | -0.330620 |
| H | 2.804134 | 5.759433 | -0.220130 |
| H | 1.710431 | 5.954468 | -1.631260 |
| H | -1.009835 | 4.261153 | 1.449060 |
| H | -1.407818 | 1.813151 | 1.462700 |
| H | 2.275367 | 0.314142 | 1.460060 |
| H | 4.196706 | -1.254157 | 1.446160 |

**[IXylH_2_]^•+^**

E° = -846.50940378 *E_h_*

G° = -846.18859578 *E_h_*

H° = -846.11658078 *E_h_*


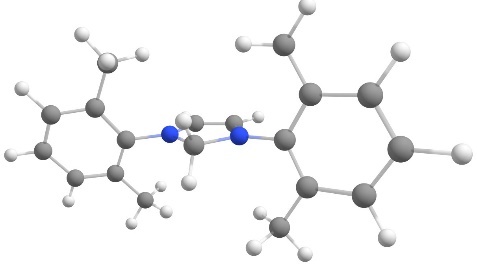


| C | 0.000001 | -0.000028 | 0.404159 |
| --- | --- | --- | --- |
| N | 1.129101 | -0.060284 | -0.507033 |
| C | 0.691738 | -0.032749 | -1.779485 |
| C | -0.691740 | 0.032689 | -1.779484 |
| N | -1.129100 | 0.060233 | -0.507031 |
| C | -2.492491 | 0.056714 | -0.058311 |
| C | 2.492492 | -0.056725 | -0.058316 |
| C | -3.191661 | -1.157952 | -0.082392 |
| C | -4.510571 | -1.143793 | 0.367678 |
| C | -5.094994 | 0.034294 | 0.821436 |
| C | -4.372972 | 1.221920 | 0.834438 |
| C | -3.049551 | 1.258646 | 0.394866 |
| C | 3.049585 | -1.258637 | 0.394875 |
| C | 4.373005 | -1.221870 | 0.834446 |
| C | 5.094995 | -0.034225 | 0.821431 |
| C | 4.510540 | 1.143842 | 0.367660 |
| C | 3.191629 | 1.157960 | -0.082409 |
| C | 2.550260 | 2.428319 | -0.582374 |
| C | 2.260492 | -2.543543 | 0.422324 |
| C | -2.550330 | -2.428335 | -0.582342 |
| C | -2.260426 | 2.543533 | 0.422298 |
| H | 1.374714 | -0.062404 | -2.615774 |
| H | -1.374718 | 0.062345 | -2.615771 |
| H | -5.083055 | -2.066275 | 0.363174 |
| H | -6.123597 | 0.026051 | 1.167037 |
| H | -4.839810 | 2.136877 | 1.186415 |
| H | 4.839868 | -2.136811 | 1.186433 |
| H | 6.123598 | -0.025949 | 1.167031 |
| H | 5.082998 | 2.066339 | 0.363147 |
| H | 3.169859 | 3.293515 | -0.336897 |
| H | 1.561021 | 2.593712 | -0.140065 |
| H | 2.423487 | 2.415562 | -1.671200 |
| H | 1.574441 | -2.626598 | -0.426921 |
| H | 1.669665 | -2.627020 | 1.343236 |
| H | 2.929707 | -3.406590 | 0.394009 |
| H | -1.561111 | -2.593772 | -0.140005 |
| H | -2.423524 | -2.415582 | -1.671165 |
| H | -3.169973 | -3.293505 | -0.336885 |
| H | -2.929620 | 3.406596 | 0.393971 |
| H | -1.574373 | 2.626560 | -0.426949 |
| H | -1.669596 | 2.627008 | 1.343208 |
| H | -0.055108 | -0.891249 | 1.044707 |
| H | 0.055112 | 0.891187 | 1.044714 |

**IXyl**

E° = -845.47215976 *E_h_*

G° = -845.17424576 *E_h_*

H° = -845.10433076 *E_h_*


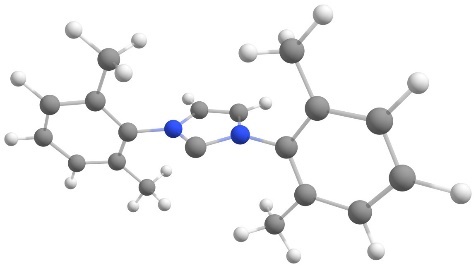


| C | 0.000000 | -0.000025 | 0.413823 |
| --- | --- | --- | --- |
| N | -1.056235 | 0.014201 | -0.455088 |
| C | -0.675015 | 0.009676 | -1.793117 |
| C | 0.675013 | -0.009665 | -1.793117 |
| N | 1.056234 | -0.014228 | -0.455088 |
| C | 2.420483 | -0.033773 | -0.026105 |
| C | -2.420484 | 0.033763 | -0.026103 |
| C | 3.139585 | 1.165563 | -0.014652 |
| C | 4.476331 | 1.123011 | 0.382799 |
| C | 5.066412 | -0.077820 | 0.760846 |
| C | 4.325153 | -1.254541 | 0.754572 |
| C | 2.987015 | -1.252603 | 0.360640 |
| C | -2.986998 | 1.252602 | 0.360640 |
| C | -4.325136 | 1.254560 | 0.754572 |
| C | -5.066412 | 0.077850 | 0.760846 |
| C | -4.476349 | -1.122990 | 0.382801 |
| C | -3.139604 | -1.165562 | -0.014650 |
| C | -2.472036 | -2.461255 | -0.397036 |
| C | -2.158422 | 2.510313 | 0.369673 |
| C | 2.472002 | 2.461246 | -0.397044 |
| C | 2.158459 | -2.510327 | 0.369670 |
| H | -1.391012 | 0.021271 | -2.600459 |
| H | 1.391010 | -0.021238 | -2.600460 |
| H | 5.054680 | 2.042887 | 0.402132 |
| H | 6.107578 | -0.095696 | 1.069506 |
| H | 4.787189 | -2.189268 | 1.060769 |
| H | -4.787158 | 2.189295 | 1.060768 |
| H | -6.107577 | 0.095742 | 1.069505 |
| H | -5.054711 | -2.042857 | 0.402133 |
| H | -3.132917 | -3.311328 | -0.206464 |
| H | -1.548704 | -2.601601 | 0.174992 |
| H | -2.196583 | -2.477089 | -1.457781 |
| H | -1.726642 | 2.710587 | -0.617551 |
| H | -1.322516 | 2.408542 | 1.069816 |
| H | -2.761122 | 3.374164 | 0.663504 |
| H | 1.548629 | 2.601549 | 0.174927 |
| H | 2.196620 | 2.477102 | -1.457807 |
| H | 3.132846 | 3.311333 | -0.206406 |
| H | 2.761172 | -3.374169 | 0.663502 |
| H | 1.726684 | -2.710607 | -0.617556 |
| H | 1.322549 | -2.408570 | 1.069811 |

**[IXyl]^•+^**

E° = -845.22955967 *E_h_*

G° = -844.93444867 *E_h_*

H° = -844.86107267 *E_h_*


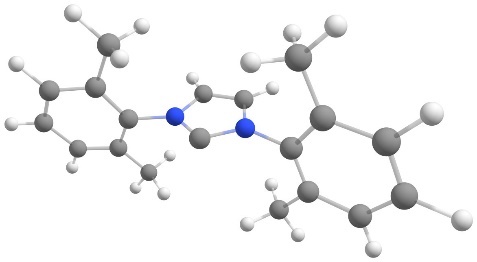


| C | 0.000019 | -0.001342 | 0.247211 |
| --- | --- | --- | --- |
| N | -1.096078 | -0.004815 | -0.490018 |
| C | -0.678733 | -0.017927 | -1.819124 |
| C | 0.678421 | -0.022592 | -1.819194 |
| N | 1.095983 | -0.011201 | -0.490141 |
| C | 2.465491 | -0.013035 | -0.018573 |
| C | -2.465446 | 0.010868 | -0.018372 |
| C | 3.107084 | 1.220916 | 0.120407 |
| C | 4.431085 | 1.191243 | 0.556755 |
| C | 5.062014 | -0.017192 | 0.833845 |
| C | 4.385524 | -1.223254 | 0.683989 |
| C | 3.060237 | -1.248686 | 0.250776 |
| C | -3.049740 | 1.253083 | 0.243655 |
| C | -4.375133 | 1.241477 | 0.677122 |
| C | -5.061782 | 0.042103 | 0.834168 |
| C | -4.441100 | -1.173233 | 0.564233 |
| C | -3.117431 | -1.216752 | 0.128029 |
| C | -2.421883 | -2.521561 | -0.164512 |
| C | -2.281392 | 2.539578 | 0.080670 |
| C | 2.400309 | 2.517981 | -0.179636 |
| C | 2.302721 | -2.542469 | 0.095180 |
| H | -1.399058 | -0.021386 | -2.622545 |
| H | 1.398639 | -0.033586 | -2.622648 |
| H | 4.969707 | 2.125553 | 0.679956 |
| H | 6.093330 | -0.018888 | 1.171254 |
| H | 4.889039 | -2.159178 | 0.904880 |
| H | -4.870707 | 2.182942 | 0.892371 |
| H | -6.093008 | 0.054514 | 1.171626 |
| H | -4.987533 | -2.102221 | 0.693162 |
| H | -3.088616 | -3.364271 | 0.028966 |
| H | -1.532192 | -2.653159 | 0.462521 |
| H | -2.101042 | -2.586366 | -1.210427 |
| H | -1.825692 | 2.621892 | -0.912381 |
| H | -1.478038 | 2.621192 | 0.822945 |
| H | -2.939550 | 3.400260 | 0.216001 |
| H | 1.516065 | 2.650810 | 0.454891 |
| H | 2.068679 | 2.569214 | -1.222929 |
| H | 3.063358 | 3.367033 | -0.001860 |
| H | 2.965803 | -3.396769 | 0.245948 |
| H | 1.856897 | -2.638993 | -0.901075 |
| H | 1.492989 | -2.621726 | 0.830678 |

**INT1 [IXylH^+^][IXylH^•^]**

E° = -1692.03412840 *E_h_*

G° = -1691.38565140 *E_h_*

H° = -1691.27118140 *E_h_*


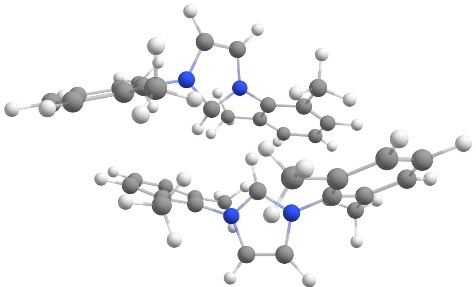


| C | 1.137021 | 0.129374 | 1.259100 |
| --- | --- | --- | --- |
| N | 2.331496 | 0.556880 | 1.670412 |
| C | 2.453542 | 0.302268 | 3.024753 |
| C | 1.300573 | -0.293296 | 3.422699 |
| N | 0.493776 | -0.387764 | 2.305843 |
| C | -0.852892 | -0.901292 | 2.248305 |
| C | 3.308901 | 1.224841 | 0.848404 |
| C | -1.900991 | 0.022764 | 2.276584 |
| C | -3.194119 | -0.481701 | 2.155488 |
| C | -3.410961 | -1.848408 | 2.015309 |
| C | -2.341545 | -2.738443 | 1.992157 |
| C | -1.027893 | -2.279579 | 2.103482 |
| C | 3.017380 | 2.517682 | 0.400437 |
| C | 3.986396 | 3.154231 | -0.374480 |
| C | 5.183437 | 2.517045 | -0.682183 |
| C | 5.435041 | 1.228148 | -0.223472 |
| C | 4.499287 | 0.550768 | 0.558684 |
| C | 4.766615 | -0.843778 | 1.063604 |
| C | 1.705629 | 3.191253 | 0.713284 |
| C | -1.640546 | 1.502198 | 2.368556 |
| C | 0.148575 | -3.218940 | 2.035466 |
| H | 0.685772 | 0.228089 | 0.267028 |
| H | 3.341559 | 0.575793 | 3.571367 |
| H | 0.974945 | -0.650470 | 4.386525 |
| H | -4.032373 | 0.207476 | 2.145998 |
| H | -4.424974 | -2.222214 | 1.914315 |
| H | -2.522577 | -3.803126 | 1.878238 |
| H | 3.797375 | 4.159422 | -0.738443 |
| H | 5.926630 | 3.028742 | -1.285160 |
| H | 6.371406 | 0.737186 | -0.471291 |
| H | 5.600735 | -1.292139 | 0.518622 |
| H | 3.895934 | -1.493903 | 0.932777 |
| H | 5.034053 | -0.843357 | 2.126572 |
| H | 1.417619 | 3.065542 | 1.762653 |
| H | 0.895067 | 2.782862 | 0.096225 |
| H | 1.764790 | 4.262212 | 0.506976 |
| H | -1.157865 | 1.862437 | 1.452352 |
| H | -0.990779 | 1.753848 | 3.214311 |
| H | -2.578269 | 2.050683 | 2.478748 |
| H | -0.189236 | -4.245778 | 1.877423 |
| H | 0.737809 | -3.201255 | 2.959080 |
| H | 0.820400 | -2.958815 | 1.208498 |
| C | -1.072716 | -0.105965 | -1.105331 |
| N | -0.321052 | -0.605844 | -2.201997 |
| C | -0.712477 | 0.054513 | -3.360919 |
| C | -1.774813 | 0.834003 | -3.068910 |
| N | -2.047566 | 0.715234 | -1.711888 |
| C | -3.268560 | 1.071296 | -1.072344 |
| C | 0.859870 | -1.368272 | -2.015270 |
| C | -4.298108 | 0.119572 | -0.995379 |
| C | -5.479201 | 0.480651 | -0.348118 |
| C | -5.625755 | 1.745522 | 0.214319 |
| C | -4.593379 | 2.672638 | 0.128219 |
| C | -3.400515 | 2.352989 | -0.524287 |
| C | 0.734012 | -2.719280 | -1.653100 |
| C | 1.899587 | -3.450892 | -1.418441 |
| C | 3.151919 | -2.861773 | -1.556993 |
| C | 3.255396 | -1.524476 | -1.923740 |
| C | 2.115161 | -0.751841 | -2.146862 |
| C | 2.235600 | 0.705201 | -2.512565 |
| C | -0.621473 | -3.367710 | -1.541027 |
| C | -4.124505 | -1.258580 | -1.578610 |
| C | -2.297483 | 3.370609 | -0.660239 |
| H | -1.454690 | -0.846729 | -0.393640 |
| H | -0.205751 | -0.105589 | -4.299817 |
| H | -2.374549 | 1.461991 | -3.709363 |
| H | -6.293937 | -0.236059 | -0.289095 |
| H | -6.552509 | 2.012078 | 0.713331 |
| H | -4.715444 | 3.662770 | 0.559333 |
| H | 1.821452 | -4.500550 | -1.148165 |
| H | 4.049251 | -3.449210 | -1.387111 |
| H | 4.233515 | -1.062804 | -2.029020 |
| H | 3.231208 | 1.083183 | -2.266933 |
| H | 1.491594 | 1.313955 | -1.986808 |
| H | 2.073239 | 0.866055 | -3.584342 |
| H | -1.278857 | -3.045974 | -2.354302 |
| H | -1.122512 | -3.100281 | -0.602339 |
| H | -0.534327 | -4.457054 | -1.572889 |
| H | -3.516687 | -1.887418 | -0.915823 |
| H | -3.618469 | -1.226911 | -2.548506 |
| H | -5.092258 | -1.749826 | -1.710281 |
| H | -2.298199 | 4.066420 | 0.184260 |
| H | -2.423988 | 3.962547 | -1.574502 |
| H | -1.318725 | 2.886589 | -0.722869 |

**TS1 [IXylH^+^][IXylH^•^]**

E° = -1692.01786384 *E_h_*

G° = -1691.37133084 *E_h_*

H° = -1691.25966684 *E_h_*

ν_imag_ = -1253.4 cm^-1^


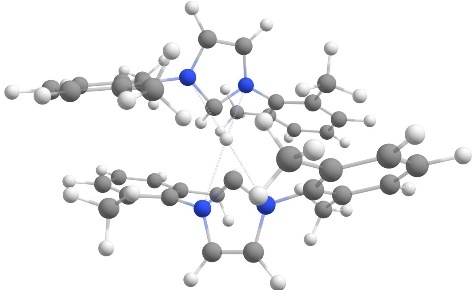


| C | -0.475720 | -1.139228 | 0.704446 |
| --- | --- | --- | --- |
| N | -1.545913 | -1.943004 | 0.858137 |
| C | -1.356309 | -2.822271 | 1.914558 |
| C | -0.136919 | -2.550972 | 2.431588 |
| N | 0.383856 | -1.517148 | 1.673889 |
| C | 1.620042 | -0.837320 | 1.948201 |
| C | -2.796851 | -1.862437 | 0.151189 |
| C | 2.824876 | -1.459728 | 1.614363 |
| C | 4.000066 | -0.750920 | 1.864280 |
| C | 3.960485 | 0.519125 | 2.425360 |
| C | 2.744569 | 1.097980 | 2.776992 |
| C | 1.545269 | 0.422089 | 2.555861 |
| C | -2.967368 | -2.607250 | -1.018269 |
| C | -4.230812 | -2.589451 | -1.610640 |
| C | -5.272238 | -1.862918 | -1.044902 |
| C | -5.064241 | -1.118557 | 0.111889 |
| C | -3.816340 | -1.100287 | 0.733526 |
| C | -3.568203 | -0.287222 | 1.977326 |
| C | -1.829955 | -3.380467 | -1.632683 |
| C | 2.859855 | -2.855127 | 1.048442 |
| C | 0.220672 | 1.012163 | 2.970419 |
| H | -0.051460 | -0.054282 | -0.114282 |
| H | -2.108620 | -3.544168 | 2.191123 |
| H | 0.403851 | -2.983735 | 3.258209 |
| H | 4.953038 | -1.199555 | 1.603622 |
| H | 4.885575 | 1.057836 | 2.604775 |
| H | 2.724873 | 2.077500 | 3.246496 |
| H | -4.399429 | -3.162807 | -2.517323 |
| H | -6.252995 | -1.875210 | -1.509899 |
| H | -5.880557 | -0.548163 | 0.545345 |
| H | -4.474232 | 0.242485 | 2.279139 |
| H | -2.788546 | 0.463037 | 1.804392 |
| H | -3.246959 | -0.914382 | 2.816251 |
| H | -1.238438 | -3.911072 | -0.880190 |
| H | -1.150048 | -2.707949 | -2.168761 |
| H | -2.203351 | -4.114432 | -2.350610 |
| H | 2.089585 | -3.006538 | 0.286682 |
| H | 2.693354 | -3.600962 | 1.834536 |
| H | 3.831496 | -3.060719 | 0.595498 |
| H | 0.370767 | 1.917690 | 3.563444 |
| H | -0.356653 | 0.305457 | 3.576364 |
| H | -0.404590 | 1.276394 | 2.109133 |
| C | 0.568606 | 1.074375 | -0.679757 |
| N | -0.407113 | 1.919768 | -1.285691 |
| C | -0.190959 | 1.976555 | -2.641325 |
| C | 0.948507 | 1.295104 | -2.924250 |
| N | 1.453569 | 0.786556 | -1.752100 |
| C | 2.760708 | 0.230432 | -1.608345 |
| C | -1.560068 | 2.385822 | -0.593969 |
| C | 3.809698 | 1.061665 | -1.195956 |
| C | 5.087130 | 0.506109 | -1.127895 |
| C | 5.304436 | -0.825950 | -1.459862 |
| C | 4.242770 | -1.632610 | -1.854335 |
| C | 2.949475 | -1.118092 | -1.930279 |
| C | -1.392967 | 3.342628 | 0.419559 |
| C | -2.527660 | 3.754432 | 1.118813 |
| C | -3.784661 | 3.256140 | 0.796952 |
| C | -3.927251 | 2.333254 | -0.232196 |
| C | -2.819103 | 1.872442 | -0.942286 |
| C | -2.986561 | 0.861238 | -2.046722 |
| C | -0.044749 | 3.948235 | 0.721309 |
| C | 3.571601 | 2.502879 | -0.828156 |
| C | 1.782607 | -1.977902 | -2.340841 |
| H | 1.071657 | 1.502226 | 0.197709 |
| H | -0.857566 | 2.511090 | -3.299397 |
| H | 1.455784 | 1.149839 | -3.865621 |
| H | 5.920907 | 1.131157 | -0.821050 |
| H | 6.308203 | -1.237289 | -1.415180 |
| H | 4.417174 | -2.673803 | -2.110964 |
| H | -2.424022 | 4.495533 | 1.906196 |
| H | -4.658038 | 3.598513 | 1.343108 |
| H | -4.910877 | 1.948566 | -0.485682 |
| H | -3.944918 | 0.348371 | -1.952084 |
| H | -2.197025 | 0.103871 | -2.027153 |
| H | -2.963891 | 1.334653 | -3.034654 |
| H | 0.547436 | 4.075527 | -0.189890 |
| H | 0.542957 | 3.328230 | 1.409269 |
| H | -0.163372 | 4.928217 | 1.190159 |
| H | 3.178456 | 2.581982 | 0.192969 |
| H | 2.851407 | 2.981867 | -1.498877 |
| H | 4.503418 | 3.072381 | -0.867020 |
| H | 0.972983 | -1.913721 | -1.604428 |
| H | 2.082069 | -3.025311 | -2.430774 |
| H | 1.365192 | -1.665266 | -3.304649 |

**INT2 [IXylH_2_^•+^][IXyl]**

E° = -1692.03104897 *E_h_*

G° = -1691.38128397 *E_h_*

H° = -1691.26741897 *E_h_*


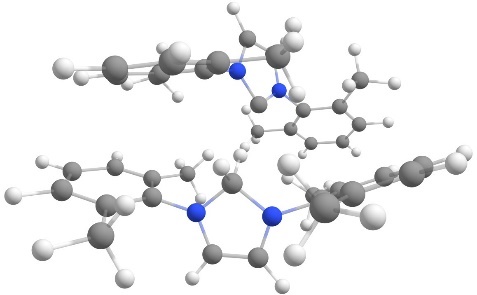


| C | 0.824064 | 1.201707 | 0.645110 |
| --- | --- | --- | --- |
| N | 1.971640 | 1.912321 | 0.810055 |
| C | 1.950307 | 2.708858 | 1.948594 |
| C | 0.749607 | 2.501974 | 2.532349 |
| N | 0.086843 | 1.587408 | 1.724528 |
| C | -1.193811 | 1.017401 | 2.015187 |
| C | 3.124622 | 1.764793 | -0.028711 |
| C | -2.348559 | 1.734422 | 1.687233 |
| C | -3.581126 | 1.125818 | 1.929128 |
| C | -3.650002 | -0.146934 | 2.483180 |
| C | -2.484892 | -0.830194 | 2.822362 |
| C | -1.232448 | -0.255607 | 2.598748 |
| C | 3.178471 | 2.451910 | -1.244769 |
| C | 4.315570 | 2.282900 | -2.036402 |
| C | 5.358930 | 1.463773 | -1.618871 |
| C | 5.279593 | 0.794452 | -0.401632 |
| C | 4.157414 | 0.930975 | 0.416256 |
| C | 4.050825 | 0.198578 | 1.728928 |
| C | 2.037545 | 3.324508 | -1.698103 |
| C | -2.259521 | 3.123919 | 1.112766 |
| C | 0.040307 | -0.986328 | 2.942470 |
| H | 2.781394 | 3.338221 | 2.226459 |
| H | 0.309873 | 2.909748 | 3.429155 |
| H | -4.492894 | 1.654873 | 1.670825 |
| H | -4.617729 | -0.604885 | 2.664503 |
| H | -2.545959 | -1.813482 | 3.281859 |
| H | 4.384770 | 2.807460 | -2.985073 |
| H | 6.240793 | 1.351266 | -2.241957 |
| H | 6.100229 | 0.160737 | -0.076145 |
| H | 4.870570 | -0.515466 | 1.842036 |
| H | 3.107018 | -0.352985 | 1.794829 |
| H | 4.087411 | 0.888380 | 2.579412 |
| H | 1.665468 | 3.958052 | -0.886845 |
| H | 1.195622 | 2.713692 | -2.042867 |
| H | 2.346728 | 3.970077 | -2.524127 |
| H | -1.546158 | 3.170712 | 0.283862 |
| H | -1.919376 | 3.840626 | 1.868852 |
| H | -3.234865 | 3.455565 | 0.749973 |
| H | -0.172921 | -1.896337 | 3.509982 |
| H | 0.709868 | -0.360963 | 3.542220 |
| H | 0.598289 | -1.265700 | 2.039990 |
| C | -0.977428 | -1.220451 | -0.564985 |
| N | -0.109227 | -2.218334 | -1.159318 |
| C | -0.506097 | -2.485149 | -2.419311 |
| C | -1.633836 | -1.738307 | -2.699807 |
| N | -1.939213 | -0.986400 | -1.623002 |
| C | -3.058938 | -0.101833 | -1.494526 |
| C | 1.111102 | -2.633478 | -0.535591 |
| C | -4.277651 | -0.627148 | -1.050662 |
| C | -5.359341 | 0.248467 | -0.966267 |
| C | -5.215743 | 1.590301 | -1.303146 |
| C | -3.984489 | 2.085503 | -1.718588 |
| C | -2.874928 | 1.246476 | -1.818637 |
| C | 1.044792 | -3.591148 | 0.483196 |
| C | 2.244005 | -3.966400 | 1.088540 |
| C | 3.450071 | -3.406923 | 0.681131 |
| C | 3.480539 | -2.452843 | -0.330297 |
| C | 2.306653 | -2.034211 | -0.954878 |
| C | 2.318993 | -0.962503 | -2.013283 |
| C | -0.270282 | -4.182930 | 0.922323 |
| C | -4.406900 | -2.073915 | -0.650489 |
| C | -1.526988 | 1.764447 | -2.246589 |
| H | -1.476130 | -1.590560 | 0.342057 |
| H | 0.027024 | -3.181664 | -3.049300 |
| H | -2.228677 | -1.708398 | -3.600787 |
| H | -6.322238 | -0.127740 | -0.633663 |
| H | -6.070919 | 2.256019 | -1.240141 |
| H | -3.879113 | 3.135870 | -1.973688 |
| H | 2.229582 | -4.710500 | 1.879360 |
| H | 4.374791 | -3.716709 | 1.157828 |
| H | 4.423291 | -2.008375 | -0.633888 |
| H | 3.323605 | -0.549664 | -2.129939 |
| H | 1.655322 | -0.137537 | -1.729556 |
| H | 1.996786 | -1.343395 | -2.989170 |
| H | -0.917821 | -4.417043 | 0.071269 |
| H | -0.818321 | -3.490421 | 1.574313 |
| H | -0.110680 | -5.103991 | 1.487904 |
| H | -3.835176 | -2.276055 | 0.263802 |
| H | -4.041632 | -2.753522 | -1.427616 |
| H | -5.450139 | -2.328148 | -0.449121 |
| H | -1.582097 | 2.828257 | -2.491499 |
| H | -1.149867 | 1.237197 | -3.130398 |
| H | -0.783326 | 1.638553 | -1.448359 |
| H | -0.385303 | -0.316021 | -0.293943 |

**[IXylH^+^][(p-MeOPh)_3_N]**

E° = -1939.16282334 *E_h_*

G° = -1938.49479134 *E_h_*

H° = -1938.37267034 *E_h_*


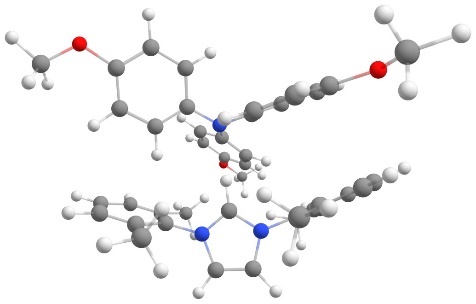


| C | -0.433905 | 0.892610 | 1.583861 |
| --- | --- | --- | --- |
| N | -1.648349 | 1.272575 | 1.987179 |
| C | -1.513109 | 2.140622 | 3.053082 |
| C | -0.183025 | 2.280126 | 3.285563 |
| N | 0.470885 | 1.491461 | 2.359039 |
| C | 1.900070 | 1.332943 | 2.240710 |
| C | -2.893054 | 0.849118 | 1.391241 |
| C | 2.504889 | 0.294494 | 2.955001 |
| C | 3.886371 | 0.157645 | 2.821956 |
| C | 4.608427 | 1.014596 | 1.997377 |
| C | 3.967596 | 2.027203 | 1.291003 |
| C | 2.589959 | 2.214732 | 1.402518 |
| C | -3.384576 | -0.415155 | 1.729772 |
| C | -4.569144 | -0.816167 | 1.116274 |
| C | -5.222974 | 0.015297 | 0.214478 |
| C | -4.720416 | 1.278718 | -0.072416 |
| C | -3.542451 | 1.731545 | 0.522540 |
| C | -3.027328 | 3.124935 | 0.265616 |
| C | -2.676560 | -1.292273 | 2.728813 |
| C | 1.694489 | -0.671647 | 3.779223 |
| C | 1.873034 | 3.298130 | 0.642627 |
| H | -0.205064 | 0.216893 | 0.762679 |
| H | -2.373851 | 2.571063 | 3.539123 |
| H | 0.361123 | 2.857181 | 4.016008 |
| H | 4.395029 | -0.634381 | 3.363089 |
| H | 5.682610 | 0.891067 | 1.902274 |
| H | 4.539750 | 2.684424 | 0.643875 |
| H | -4.978147 | -1.796101 | 1.340420 |
| H | -6.134021 | -0.326122 | -0.265665 |
| H | -5.247519 | 1.928871 | -0.764123 |
| H | -3.430535 | 3.513374 | -0.673244 |
| H | -1.935483 | 3.163308 | 0.205975 |
| H | -3.340524 | 3.810134 | 1.062332 |
| H | -2.539792 | -0.778971 | 3.687510 |
| H | -1.689842 | -1.598705 | 2.366940 |
| H | -3.252683 | -2.201562 | 2.914442 |
| H | 1.125014 | -1.348861 | 3.130389 |
| H | 0.980682 | -0.162157 | 4.434846 |
| H | 2.345188 | -1.286536 | 4.404714 |
| H | 2.570930 | 3.859338 | 0.018239 |
| H | 1.382667 | 4.006161 | 1.320949 |
| H | 1.105605 | 2.880417 | -0.017456 |
| C | -4.669058 | -4.569569 | -0.166713 |
| O | -4.472442 | -3.498527 | -1.063823 |
| C | -3.267781 | -2.877366 | -1.058943 |
| C | -2.243694 | -3.132465 | -0.147849 |
| C | -1.070226 | -2.384715 | -0.201509 |
| C | -0.876104 | -1.401837 | -1.171835 |
| N | 0.367779 | -0.697438 | -1.182501 |
| C | 1.541380 | -1.525500 | -1.140192 |
| C | 1.663761 | -2.636873 | -1.981460 |
| C | 2.792259 | -3.434458 | -1.927721 |
| C | 3.836267 | -3.128492 | -1.043929 |
| O | 4.895190 | -3.967057 | -1.072872 |
| C | 5.983211 | -3.700126 | -0.214987 |
| C | 3.726494 | -2.011389 | -0.213849 |
| C | 2.576002 | -1.226524 | -0.264128 |
| C | 0.480180 | 0.593775 | -1.765897 |
| C | -0.564936 | 1.518470 | -1.680514 |
| C | -0.450329 | 2.800918 | -2.221093 |
| C | 0.735669 | 3.199815 | -2.834752 |
| O | 0.963624 | 4.422326 | -3.371103 |
| C | -0.086463 | 5.363316 | -3.345685 |
| C | 1.790209 | 2.284643 | -2.912037 |
| C | 1.664376 | 1.005887 | -2.398070 |
| C | -1.891819 | -1.189156 | -2.112856 |
| C | -3.075274 | -1.904240 | -2.044374 |
| H | -5.669515 | -4.952817 | -0.367577 |
| H | -4.614244 | -4.236424 | 0.879245 |
| H | -3.934425 | -5.368127 | -0.327784 |
| H | -2.344385 | -3.896074 | 0.614913 |
| H | -0.271560 | -2.597760 | 0.504485 |
| H | 0.861394 | -2.878194 | -2.671879 |
| H | 2.896729 | -4.301253 | -2.571379 |
| H | 5.680132 | -3.735588 | 0.839492 |
| H | 6.715377 | -4.484802 | -0.405087 |
| H | 6.434242 | -2.723402 | -0.432210 |
| H | 4.515911 | -1.739875 | 0.477094 |
| H | 2.501946 | -0.349698 | 0.368742 |
| H | -1.505929 | 1.231554 | -1.221425 |
| H | -1.299250 | 3.471543 | -2.155264 |
| H | 0.299044 | 6.259253 | -3.832264 |
| H | -0.963578 | 5.001299 | -3.897283 |
| H | -0.380762 | 5.609448 | -2.316355 |
| H | 2.707925 | 2.592296 | -3.402313 |
| H | 2.495313 | 0.317075 | -2.492907 |
| H | -1.755501 | -0.463393 | -2.906966 |
| H | -3.867693 | -1.733521 | -2.765293 |

**[IXylH^+^][(*p*-MeOPh)_3_N] excited singlet state**

E° = -1939.04989255 *E_h_*

G° = -1938.37987455 *E_h_*

H° = -1938.26034855 *E_h_*


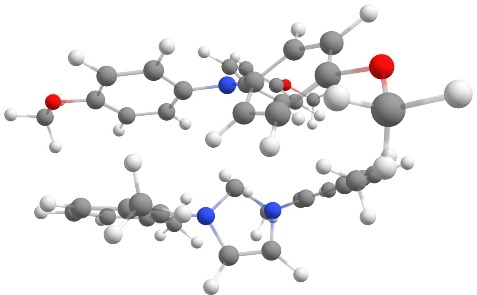


| C | 0.027520 | -0.263808 | 1.420031 |
| --- | --- | --- | --- |
| N | -1.102176 | 0.199924 | 2.143082 |
| C | -0.666096 | 0.889000 | 3.277513 |
| C | 0.675375 | 0.813888 | 3.335623 |
| N | 1.124530 | 0.060279 | 2.257807 |
| C | 2.479532 | -0.313009 | 2.043724 |
| C | -2.453053 | -0.207525 | 1.948483 |
| C | 2.873841 | -1.645104 | 2.240179 |
| C | 4.214290 | -1.977940 | 2.034974 |
| C | 5.139780 | -1.015323 | 1.649082 |
| C | 4.731586 | 0.300558 | 1.456744 |
| C | 3.400960 | 0.672581 | 1.652605 |
| C | -2.813730 | -1.547244 | 2.185984 |
| C | -4.146957 | -1.920565 | 2.022996 |
| C | -5.111053 | -0.988501 | 1.655636 |
| C | -4.744962 | 0.332550 | 1.444807 |
| C | -3.419414 | 0.746851 | 1.591560 |
| C | -3.102519 | 2.208553 | 1.385342 |
| C | -1.807845 | -2.563603 | 2.659646 |
| C | 1.889927 | -2.695624 | 2.679742 |
| C | 2.962005 | 2.097587 | 1.434050 |
| H | -0.007438 | -1.284629 | 1.033282 |
| H | -1.366630 | 1.359396 | 3.948716 |
| H | 1.362659 | 1.220484 | 4.060223 |
| H | 4.535982 | -3.002177 | 2.204730 |
| H | 6.181708 | -1.286787 | 1.508437 |
| H | 5.454099 | 1.053982 | 1.155622 |
| H | -4.436064 | -2.948516 | 2.226521 |
| H | -6.151867 | -1.284636 | 1.564588 |
| H | -5.499169 | 1.066489 | 1.174155 |
| H | -3.201644 | 2.478038 | 0.327213 |
| H | -2.096624 | 2.471793 | 1.717891 |
| H | -3.810182 | 2.831787 | 1.941439 |
| H | -1.098412 | -2.120196 | 3.363660 |
| H | -1.223088 | -2.979934 | 1.829938 |
| H | -2.309940 | -3.396878 | 3.157993 |
| H | 1.265825 | -3.034158 | 1.843735 |
| H | 1.217742 | -2.309149 | 3.450870 |
| H | 2.408278 | -3.570046 | 3.081312 |
| H | 3.691229 | 2.640985 | 0.827174 |
| H | 2.855230 | 2.634424 | 2.384108 |
| H | 1.989690 | 2.139307 | 0.935396 |
| C | -5.936617 | -2.150741 | -1.601246 |
| O | -5.436916 | -0.851181 | -1.880805 |
| C | -4.119459 | -0.641396 | -1.824051 |
| C | -3.181649 | -1.575130 | -1.364896 |
| C | -1.837046 | -1.252632 | -1.358600 |
| C | -1.392185 | -0.009144 | -1.833083 |
| N | -0.026884 | 0.298326 | -1.857054 |
| C | 0.931439 | -0.711488 | -2.039351 |
| C | 0.706564 | -1.782570 | -2.925903 |
| C | 1.649090 | -2.777223 | -3.051944 |
| C | 2.839490 | -2.733398 | -2.298499 |
| O | 3.676212 | -3.761153 | -2.473682 |
| C | 4.896132 | -3.775557 | -1.747420 |
| C | 3.077563 | -1.654636 | -1.438003 |
| C | 2.128094 | -0.655906 | -1.316554 |
| C | 0.403842 | 1.619390 | -1.649595 |
| C | -0.233152 | 2.416423 | -0.694492 |
| C | 0.178459 | 3.720391 | -0.471235 |
| C | 1.257074 | 4.239887 | -1.199008 |
| O | 1.749304 | 5.474936 | -1.057609 |
| C | 1.150314 | 6.349474 | -0.114415 |
| C | 1.902614 | 3.435507 | -2.158879 |
| C | 1.484238 | 2.144336 | -2.383082 |
| C | -2.341498 | 0.924834 | -2.297268 |
| C | -3.680918 | 0.615789 | -2.282464 |
| H | -7.012915 | -2.091952 | -1.757297 |
| H | -5.729170 | -2.436271 | -0.565618 |
| H | -5.505147 | -2.888208 | -2.287292 |
| H | -3.494870 | -2.533526 | -0.972407 |
| H | -1.121971 | -1.965612 | -0.967095 |
| H | -0.199007 | -1.811817 | -3.522583 |
| H | 1.502601 | -3.603948 | -3.737822 |
| H | 4.710193 | -3.767391 | -0.667513 |
| H | 5.395594 | -4.701642 | -2.028272 |
| H | 5.525114 | -2.920988 | -2.020625 |
| H | 3.975478 | -1.590587 | -0.836971 |
| H | 2.296727 | 0.155430 | -0.622476 |
| H | -1.024640 | 1.986282 | -0.099259 |
| H | -0.324591 | 4.307902 | 0.286902 |
| H | 1.706913 | 7.282995 | -0.182621 |
| H | 0.098076 | 6.531970 | -0.359594 |
| H | 1.232770 | 5.947021 | 0.901535 |
| H | 2.721704 | 3.865396 | -2.724136 |
| H | 1.970498 | 1.537469 | -3.139424 |
| H | -2.010794 | 1.875693 | -2.700070 |
| H | -4.420472 | 1.314485 | -2.656822 |

**[IXylH^+^][(*p*-MeOPh)_3_N] triplet state**

E° = -1939.06520118 *E_h_*

G° = -1938.40076418 *E_h_*

H° = -1938.27781718 *E_h_*


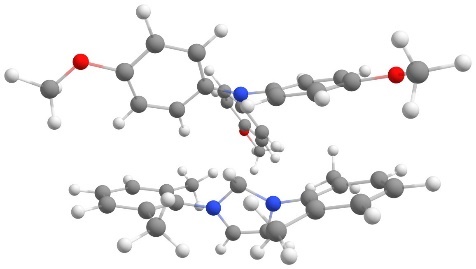


| C | -0.044349 | -0.203022 | 1.453885 |
| --- | --- | --- | --- |
| N | -1.189490 | 0.327072 | 2.106987 |
| C | -0.769627 | 1.117016 | 3.178559 |
| C | 0.568946 | 1.021327 | 3.286162 |
| N | 1.034058 | 0.150884 | 2.306908 |
| C | 2.388910 | -0.226876 | 2.128674 |
| C | -2.537734 | -0.063531 | 1.884049 |
| C | 2.768824 | -1.563438 | 2.340166 |
| C | 4.110824 | -1.907608 | 2.172486 |
| C | 5.055569 | -0.955405 | 1.806087 |
| C | 4.662015 | 0.361348 | 1.590311 |
| C | 3.329679 | 0.746722 | 1.745836 |
| C | -2.929154 | -1.386304 | 2.171049 |
| C | -4.258600 | -1.750725 | 1.964291 |
| C | -5.192115 | -0.827728 | 1.505588 |
| C | -4.797830 | 0.477025 | 1.248168 |
| C | -3.474687 | 0.883047 | 1.432932 |
| C | -3.125126 | 2.323785 | 1.146948 |
| C | -1.959763 | -2.389187 | 2.739069 |
| C | 1.767024 | -2.604643 | 2.760644 |
| C | 2.911149 | 2.170468 | 1.481651 |
| H | -0.090826 | -1.239488 | 1.113913 |
| H | -1.478781 | 1.652661 | 3.790604 |
| H | 1.239960 | 1.473267 | 3.999976 |
| H | 4.418984 | -2.933484 | 2.357951 |
| H | 6.099014 | -1.235920 | 1.697114 |
| H | 5.397479 | 1.105905 | 1.297937 |
| H | -4.571112 | -2.763908 | 2.205569 |
| H | -6.230297 | -1.117366 | 1.373609 |
| H | -5.528103 | 1.204148 | 0.903172 |
| H | -3.092089 | 2.504140 | 0.065375 |
| H | -2.163051 | 2.618027 | 1.571482 |
| H | -3.890004 | 2.988623 | 1.560171 |
| H | -1.266571 | -1.915629 | 3.440137 |
| H | -1.354598 | -2.863914 | 1.956322 |
| H | -2.494158 | -3.183009 | 3.267718 |
| H | 1.184853 | -2.969337 | 1.905023 |
| H | 1.057703 | -2.198752 | 3.487278 |
| H | 2.267548 | -3.466388 | 3.209893 |
| H | 3.655202 | 2.685366 | 0.867239 |
| H | 2.800714 | 2.739444 | 2.412429 |
| H | 1.946902 | 2.210001 | 0.966862 |
| C | -5.781938 | -2.455333 | -1.529063 |
| O | -5.328493 | -1.146937 | -1.845564 |
| C | -4.020594 | -0.887340 | -1.798007 |
| C | -3.046170 | -1.779830 | -1.325218 |
| C | -1.714209 | -1.415910 | -1.342987 |
| C | -1.315361 | -0.164893 | -1.846750 |
| N | 0.032918 | 0.187442 | -1.894316 |
| C | 1.033778 | -0.789169 | -2.013913 |
| C | 0.874226 | -1.899041 | -2.865885 |
| C | 1.859957 | -2.858044 | -2.932491 |
| C | 3.025851 | -2.738092 | -2.150791 |
| O | 3.908649 | -3.735416 | -2.264990 |
| C | 5.102204 | -3.678734 | -1.497636 |
| C | 3.196463 | -1.620628 | -1.321342 |
| C | 2.207109 | -0.657300 | -1.260908 |
| C | 0.419254 | 1.533673 | -1.727724 |
| C | -0.189724 | 2.311383 | -0.740584 |
| C | 0.181904 | 3.633573 | -0.558014 |
| C | 1.189132 | 4.187765 | -1.361006 |
| O | 1.634962 | 5.445687 | -1.264051 |
| C | 1.064132 | 6.302620 | -0.288493 |
| C | 1.809834 | 3.397780 | -2.346843 |
| C | 1.432633 | 2.085492 | -2.529320 |
| C | -2.302060 | 0.732257 | -2.311526 |
| C | -3.629352 | 0.377473 | -2.278747 |
| H | -6.859735 | -2.438554 | -1.684982 |
| H | -5.564518 | -2.703293 | -0.485471 |
| H | -5.325127 | -3.195919 | -2.194923 |
| H | -3.324726 | -2.739826 | -0.910037 |
| H | -0.971143 | -2.094664 | -0.940482 |
| H | -0.014307 | -1.986011 | -3.482563 |
| H | 1.765153 | -3.714381 | -3.590705 |
| H | 4.878053 | -3.639362 | -0.425463 |
| H | 5.644199 | -4.594926 | -1.727567 |
| H | 5.709372 | -2.811472 | -1.780163 |
| H | 4.073164 | -1.501189 | -0.697471 |
| H | 2.321708 | 0.183703 | -0.589036 |
| H | -0.911179 | 1.847971 | -0.081429 |
| H | -0.292826 | 4.208809 | 0.227821 |
| H | 1.574067 | 7.258435 | -0.400323 |
| H | -0.009335 | 6.438395 | -0.462183 |
| H | 1.232086 | 5.914221 | 0.722711 |
| H | 2.578672 | 3.851143 | -2.962267 |
| H | 1.901577 | 1.483353 | -3.300643 |
| H | -2.006808 | 1.689097 | -2.727507 |
| H | -4.396600 | 1.045782 | -2.653132 |

**[IDippH]^•^**

E° = -1160.55151082 *E_h_*

G° = -1160.02440182 *E_h_*

H° = -1159.93160482 *E_h_*


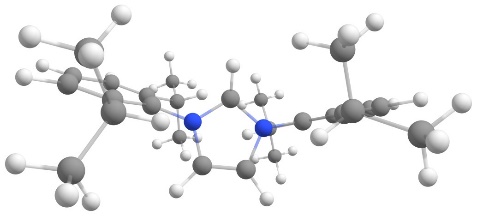


| C | -0.036722 | 0.110254 | 0.454091 |
| --- | --- | --- | --- |
| N | 1.096991 | 0.227242 | -0.383384 |
| C | 0.685852 | 0.448404 | -1.693924 |
| C | -0.656120 | 0.558679 | -1.705758 |
| N | -1.123161 | 0.439486 | -0.397152 |
| C | -2.474670 | 0.115492 | -0.072705 |
| C | 2.438328 | 0.007666 | 0.026357 |
| C | -2.887376 | -1.226685 | -0.055297 |
| C | -4.210490 | -1.500976 | 0.296306 |
| C | -5.095336 | -0.476659 | 0.604418 |
| C | -4.672954 | 0.847058 | 0.561610 |
| C | -3.358532 | 1.168054 | 0.222458 |
| C | 2.852406 | -1.286804 | 0.404500 |
| C | 4.185100 | -1.462051 | 0.771888 |
| C | 5.083740 | -0.399504 | 0.753756 |
| C | 4.654257 | 0.867258 | 0.389201 |
| C | 3.322262 | 1.099039 | 0.037052 |
| C | 2.860145 | 2.506211 | -0.307146 |
| C | 1.876572 | -2.453355 | 0.364462 |
| C | -1.942194 | -2.363570 | -0.408292 |
| C | -2.871814 | 2.609413 | 0.216688 |
| H | 0.013392 | 0.611680 | 1.425980 |
| H | 1.400139 | 0.503017 | -2.501118 |
| H | -1.337672 | 0.709682 | -2.528613 |
| H | -4.553663 | -2.532005 | 0.323626 |
| H | -6.121568 | -0.708956 | 0.874429 |
| H | -5.377847 | 1.638069 | 0.798182 |
| H | 4.535035 | -2.445495 | 1.068593 |
| H | 6.120835 | -0.563070 | 1.032340 |
| H | 5.359301 | 1.694443 | 0.391375 |
| C | 3.150984 | 3.489784 | 0.834355 |
| H | 1.774880 | 2.478606 | -0.441475 |
| C | 3.481510 | 2.989996 | -1.624293 |
| C | 1.695372 | -2.961320 | -1.074451 |
| H | 0.908064 | -2.066570 | 0.700281 |
| C | 2.256486 | -3.608612 | 1.293563 |
| H | -0.987090 | -1.929331 | -0.711394 |
| C | -2.462987 | -3.186468 | -1.593043 |
| C | -1.669430 | -3.248501 | 0.813761 |
| C | -3.940936 | 3.611341 | -0.231266 |
| H | -2.041103 | 2.672811 | -0.493251 |
| C | -2.319606 | 2.987623 | 1.599498 |
| H | -2.586073 | -3.733027 | 1.170793 |
| H | -1.254973 | -2.652322 | 1.633047 |
| H | -0.949113 | -4.036022 | 0.562159 |
| H | -3.401899 | -3.698860 | -1.352966 |
| H | -1.728675 | -3.950728 | -1.872497 |
| H | -2.640473 | -2.549116 | -2.465709 |
| H | -4.395625 | 3.315191 | -1.182481 |
| H | -3.491230 | 4.601716 | -0.360527 |
| H | -4.741566 | 3.715560 | 0.510157 |
| H | -1.944211 | 4.017443 | 1.597406 |
| H | -1.497695 | 2.325965 | 1.889860 |
| H | -3.103626 | 2.910262 | 2.362159 |
| H | 3.110150 | 3.988117 | -1.882532 |
| H | 3.239388 | 2.311922 | -2.449631 |
| H | 4.573928 | 3.045995 | -1.548797 |
| H | 1.335925 | -2.169075 | -1.738405 |
| H | 0.963185 | -3.777683 | -1.099861 |
| H | 2.643318 | -3.341602 | -1.474039 |
| H | 2.449315 | -3.259379 | 2.313497 |
| H | 3.144751 | -4.147121 | 0.941609 |
| H | 1.435065 | -4.332123 | 1.334001 |
| H | 2.702314 | 3.145295 | 1.771624 |
| H | 2.737802 | 4.476942 | 0.598425 |
| H | 4.227412 | 3.612081 | 1.000764 |

**[IDippH_2_]^•+^**

E° = -1161.01732410 *E_h_*

G° = -1160.48012210 *E_h_*

H° = -1160.38314610 *E_h_*


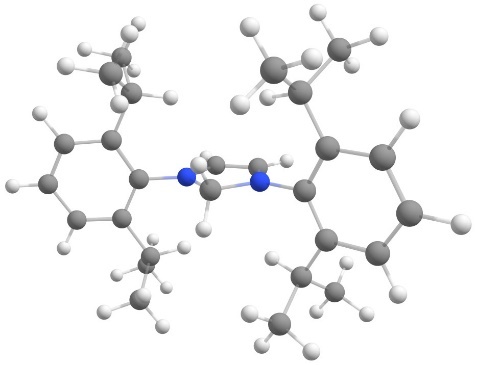


| C | 0.000000 | 0.000933 | -0.302660 |
| --- | --- | --- | --- |
| N | 1.127853 | 0.000793 | 0.606027 |
| C | 0.692883 | 0.000747 | 1.878763 |
| C | -0.692883 | 0.000751 | 1.878763 |
| N | -1.127853 | 0.000801 | 0.606027 |
| C | -2.482688 | 0.000168 | 0.132871 |
| C | 2.482689 | 0.000163 | 0.132872 |
| C | -3.098541 | 1.236838 | -0.109552 |
| C | -4.402113 | 1.206347 | -0.604600 |
| C | -5.045920 | -0.001100 | -0.847234 |
| C | -4.400937 | -1.207911 | -0.604554 |
| C | -3.097338 | -1.237116 | -0.109508 |
| C | 3.098542 | 1.236835 | -0.109542 |
| C | 4.402116 | 1.206347 | -0.604587 |
| C | 5.045922 | -0.001098 | -0.847228 |
| C | 4.400939 | -1.207911 | -0.604559 |
| C | 3.097339 | -1.237120 | -0.109515 |
| C | 2.383821 | -2.560890 | 0.118875 |
| C | 2.386279 | 2.561283 | 0.118784 |
| C | -2.386277 | 2.561287 | 0.118765 |
| C | -2.383823 | -2.560885 | 0.118898 |
| H | -0.000003 | -0.893519 | -0.943022 |
| H | 1.378629 | 0.000645 | 2.713046 |
| H | -1.378629 | 0.000652 | 2.713045 |
| H | -4.919887 | 2.138712 | -0.807646 |
| H | -6.060059 | -0.001602 | -1.233749 |
| H | -4.917806 | -2.140789 | -0.807555 |
| H | 4.919890 | 2.138713 | -0.807626 |
| H | 6.060062 | -0.001598 | -1.233741 |
| H | 4.917809 | -2.140788 | -0.807565 |
| C | 2.093242 | -3.260894 | -1.217289 |
| H | 1.418441 | -2.357027 | 0.598120 |
| C | 3.168393 | -3.476190 | 1.068026 |
| C | 3.171822 | 3.476012 | 1.067681 |
| H | 1.420788 | 2.358341 | 0.598193 |
| C | 2.096156 | 3.261344 | -1.217451 |
| H | -1.420790 | 2.358349 | 0.598184 |
| C | -3.171826 | 3.476029 | 1.067645 |
| C | -2.096142 | 3.261333 | -1.217476 |
| C | -3.168392 | -3.476162 | 1.068075 |
| H | -1.418437 | -2.357020 | 0.598129 |
| C | -2.093262 | -3.260917 | -1.217255 |
| H | -3.025177 | 3.524671 | -1.734042 |
| H | -1.515117 | 2.621874 | -1.892085 |
| H | -1.531863 | 4.184493 | -1.049889 |
| H | -4.130493 | 3.776949 | 0.632812 |
| H | -2.601345 | 4.387948 | 1.270447 |
| H | -3.376948 | 2.979405 | 2.021275 |
| H | -3.373752 | -2.979567 | 2.021669 |
| H | -2.597089 | -4.387561 | 1.270904 |
| H | -4.126889 | -3.777962 | 0.633477 |
| H | -1.528038 | -4.183485 | -1.049578 |
| H | -1.512987 | -2.621039 | -1.892109 |
| H | -3.022132 | -3.525280 | -1.733592 |
| H | 2.597085 | -4.387588 | 1.270845 |
| H | 3.373766 | -2.979614 | 2.021627 |
| H | 4.126883 | -3.777989 | 0.633413 |
| H | 3.376934 | 2.979377 | 2.021308 |
| H | 2.601344 | 4.387931 | 1.270488 |
| H | 4.130496 | 3.776931 | 0.632861 |
| H | 1.515133 | 2.621896 | -1.892070 |
| H | 3.025195 | 3.524684 | -1.734008 |
| H | 1.531879 | 4.184505 | -1.049857 |
| H | 1.512974 | -2.620994 | -1.892129 |
| H | 1.528005 | -4.183456 | -1.049627 |
| H | 3.022106 | -3.525260 | -1.733635 |
| H | 0.000004 | 0.895594 | -0.942741 |

**[IXyl^•+^,H_2_]**

E° = -846.408800155 E*_h_*

G° = -846.104833155 *E_h_*

H° = -846.025275155 *E_h_*


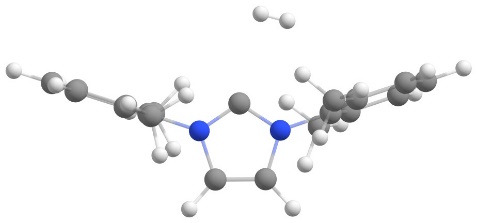


| C | 0.047908 | 0.042192 | 0.250276 |
| --- | --- | --- | --- |
| N | 1.121927 | 0.063764 | -0.518285 |
| C | 0.670474 | 0.135859 | -1.833580 |
| C | -0.685520 | 0.152700 | -1.795970 |
| N | -1.067106 | 0.088409 | -0.457934 |
| C | -2.423635 | 0.061945 | 0.045598 |
| C | 2.500435 | -0.007098 | -0.081459 |
| C | -3.031208 | -1.186668 | 0.206161 |
| C | -4.341677 | -1.188117 | 0.681646 |
| C | -4.993218 | 0.005439 | 0.973778 |
| C | -4.350564 | 1.226670 | 0.802145 |
| C | -3.039432 | 1.283041 | 0.331255 |
| C | 3.017468 | -1.266120 | 0.235473 |
| C | 4.351885 | -1.311781 | 0.636378 |
| C | 5.112325 | -0.149931 | 0.710384 |
| C | 4.557348 | 1.084038 | 0.389038 |
| C | 3.227496 | 1.184554 | -0.017066 |
| C | 2.605708 | 2.512469 | -0.365640 |
| C | 2.171122 | -2.511375 | 0.163987 |
| C | -2.297566 | -2.465926 | -0.102771 |
| C | -2.315919 | 2.593002 | 0.154856 |
| H | 1.368090 | 0.165578 | -2.656132 |
| H | -1.426646 | 0.204148 | -2.578466 |
| H | -4.853507 | -2.134818 | 0.822951 |
| H | -6.013925 | -0.016616 | 1.341255 |
| H | -4.869444 | 2.150636 | 1.037164 |
| H | 4.796449 | -2.268712 | 0.891166 |
| H | 6.149624 | -0.206546 | 1.023677 |
| H | 5.160561 | 1.984043 | 0.455084 |
| H | 3.292162 | 3.329578 | -0.135360 |
| H | 1.681075 | 2.686808 | 0.196018 |
| H | 2.361133 | 2.578613 | -1.431876 |
| H | 1.637539 | -2.590651 | -0.789549 |
| H | 1.423920 | -2.531861 | 0.966916 |
| H | 2.790375 | -3.403910 | 0.273576 |
| H | -1.422819 | -2.591462 | 0.546739 |
| H | -1.945542 | -2.494732 | -1.139935 |
| H | -2.947694 | -3.329400 | 0.050700 |
| H | -2.979125 | 3.431622 | 0.375565 |
| H | -1.946586 | 2.721639 | -0.868565 |
| H | -1.454907 | 2.667170 | 0.829707 |
| H | -0.545850 | -0.690477 | 2.761366 |
| H | -1.265740 | -0.622562 | 2.580249 |

**TS2 [IXyl···H···H]^•+^**

E° = -846.40793567 *E_h_*

G° = -846.10267467 *E_h_*

H° = -846.026105666 *E_h_*

ν_imag_ = -415.5 cm^-1^


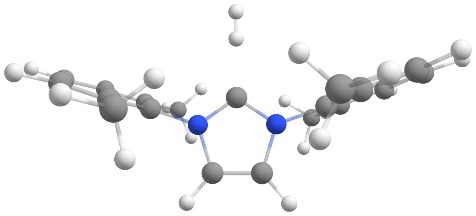


| C | 0.000000 | 0.000005 | -0.220902 |
| --- | --- | --- | --- |
| N | -1.090576 | 0.009584 | 0.530833 |
| C | -0.678609 | 0.005578 | 1.857614 |
| C | 0.678600 | -0.005560 | 1.857616 |
| N | 1.090573 | -0.009572 | 0.530838 |
| C | 2.456682 | -0.015995 | 0.055339 |
| C | -2.456683 | 0.015999 | 0.055329 |
| C | 3.095592 | -1.251953 | -0.076267 |
| C | 4.415659 | -1.230435 | -0.524015 |
| C | 5.046928 | -0.027060 | -0.819832 |
| C | 4.374046 | 1.181566 | -0.678129 |
| C | 3.052841 | 1.214294 | -0.233952 |
| C | -3.052829 | -1.214291 | -0.233983 |
| C | -4.374032 | -1.181569 | -0.678164 |
| C | -5.046926 | 0.027052 | -0.819853 |
| C | -4.415670 | 1.230429 | -0.524015 |
| C | -3.095606 | 1.251953 | -0.076261 |
| C | -2.389249 | 2.544010 | 0.244865 |
| C | -2.299509 | -2.511442 | -0.087262 |
| C | 2.389220 | -2.544007 | 0.244838 |
| C | 2.299530 | 2.511448 | -0.087213 |
| H | -1.397342 | 0.009860 | 2.662245 |
| H | 1.397331 | -0.009839 | 2.662251 |
| H | 4.951167 | -2.167322 | -0.641256 |
| H | 6.075478 | -0.031388 | -1.165515 |
| H | 4.877660 | 2.113949 | -0.913416 |
| H | -4.877636 | -2.113954 | -0.913467 |
| H | -6.075475 | 0.031375 | -1.165540 |
| H | -4.951187 | 2.167312 | -0.641244 |
| H | -3.038564 | 3.397974 | 0.042307 |
| H | -1.481260 | 2.667743 | -0.356788 |
| H | -2.094215 | 2.594717 | 1.298928 |
| H | -1.850546 | -2.613996 | 0.906702 |
| H | -1.491745 | -2.589693 | -0.824937 |
| H | -2.965714 | -3.362747 | -0.240439 |
| H | 1.481237 | -2.667725 | -0.356827 |
| H | 2.094172 | -2.594721 | 1.298897 |
| H | 3.038530 | -3.397975 | 0.042279 |
| H | 2.965730 | 3.362750 | -0.240426 |
| H | 1.850609 | 2.614012 | 0.906769 |
| H | 1.491735 | 2.589694 | -0.824854 |
| H | 0.000009 | -0.000011 | -1.894472 |
| H | 0.000015 | -0.000019 | -2.683070 |

**[IXylH^+^,H^•^]**

E° = -846.447768185 *E_h_*

G° = -846.143236185 *E_h_*

H° = -846.062957185 *E_h_*


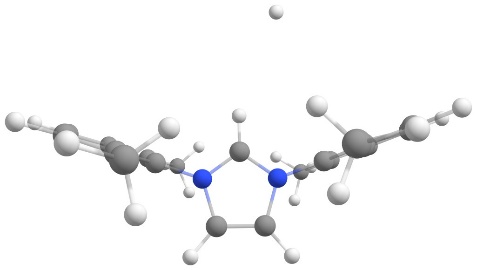


| C | -0.006703 | -0.001171 | -0.236553 |
| --- | --- | --- | --- |
| N | 1.074384 | -0.014385 | 0.542363 |
| C | 0.665342 | -0.045214 | 1.860512 |
| C | -0.693863 | -0.050372 | 1.855479 |
| N | -1.093375 | -0.022351 | 0.534351 |
| C | -2.457289 | -0.017518 | 0.055300 |
| C | 2.441670 | 0.005853 | 0.073512 |
| C | -3.099729 | 1.217738 | -0.075150 |
| C | -4.417110 | 1.195897 | -0.531593 |
| C | -5.044879 | -0.007623 | -0.835666 |
| C | -4.371201 | -1.215880 | -0.691991 |
| C | -3.052330 | -1.247930 | -0.239707 |
| C | 3.028351 | 1.249245 | -0.180969 |
| C | 4.350695 | 1.241459 | -0.624162 |
| C | 5.035631 | 0.043622 | -0.798227 |
| C | 4.415867 | -1.173476 | -0.534348 |
| C | 3.095504 | -1.219674 | -0.088534 |
| C | 2.405193 | -2.527638 | 0.203992 |
| C | 2.265352 | 2.536835 | 0.001678 |
| C | -2.400644 | 2.510572 | 0.260719 |
| C | -2.301430 | -2.546505 | -0.087504 |
| H | 1.379009 | -0.058855 | 2.668978 |
| H | -1.413404 | -0.072026 | 2.658556 |
| H | -4.953714 | 2.132311 | -0.647975 |
| H | -6.071264 | -0.003763 | -1.187828 |
| H | -4.872493 | -2.148474 | -0.931555 |
| H | 4.845743 | 2.184861 | -0.832402 |
| H | 6.064527 | 0.058609 | -1.142680 |
| H | 4.961118 | -2.101618 | -0.674444 |
| H | 3.064039 | -3.368826 | -0.020843 |
| H | 1.495912 | -2.649697 | -0.396208 |
| H | 2.115233 | -2.606123 | 1.257876 |
| H | 1.785648 | 2.593389 | 0.984900 |
| H | 1.480506 | 2.648432 | -0.756302 |
| H | 2.933065 | 3.395735 | -0.092372 |
| H | -1.494177 | 2.649546 | -0.340144 |
| H | -2.104180 | 2.549203 | 1.314997 |
| H | -3.056003 | 3.362874 | 0.070185 |
| H | -2.970431 | -3.396831 | -0.234918 |
| H | -1.851036 | -2.645007 | 0.906267 |
| H | -1.494932 | -2.632720 | -0.825767 |
| H | -0.002480 | 0.023760 | -1.315343 |
| H | 1.141676 | 0.653064 | -4.320715 |

# V. References

[1] S. Stoll, A. Schweiger, “EasySpin, a comprehensive software package for spectral simulation and analysis in EPR” *Journal of Magnetic Resonance* **2006**, *178*, 42–55.

[2] T. Casey, cwEPR 3.6.0, MATLAB Central File Exchange.

[3] R. M. Stolley, H. A. Duong, D. R. Thomas, J. Louie, “The Discovery of [Ni(NHC)RCN] _2_ Species and Their Role as Cycloaddition Catalysts for the Formation of Pyridines” *J. Am. Chem. Soc.* **2012**, *134*, 15154–15162.

[4] A. A. Kelkar, N. M. Patil, R. V Chaudhari, “Copper-catalyzed amination of aryl halides: single-step synthesis of triarylamines” *Tetrahedron Lett.* **2002**, *43*, 7143–7146.

[5] Bruker, SAINT, V8.40B, Bruker AXS Inc., Madison, Wisconsin, USA.

[6] L. Krause, R. Herbst-Irmer, G. M. Sheldrick, D. Stalke, “Comparison of silver and molybdenum microfocus X-ray sources for single-crystal structure determination” *J. Appl. Crystallogr.* **2015**, *48*, 3–10.

[7] G. M. Sheldrick, “*SHELXT* – Integrated space-group and crystal-structure determination” *Acta Crystallogr. A Found. Adv.* **2015**, *71*, 3–8.

[8] G. M. Sheldrick, “Crystal structure refinement with *SHELXL*” *Acta Crystallogr. C Struct. Chem.* **2015**, *71*, 3–8.

[9] J. D. Chai, M. Head-Gordon, “Long-range corrected hybrid density functionals with damped atom–atom dispersion corrections.” *Physical Chemistry Chemical Physics* **2008**, *10*, 6615–6620.

[10] J.-P. Blaudeau, M. P. McGrath, L. A. Curtiss, L. Radom, “Extension of Gaussian-2 (G2) theory to molecules containing third-row atoms K and Ca” *J. Chem. Phys.* **1997**, *107*, 5016–5021.

[11] M. M. Francl, W. J. Pietro, W. J. Hehre, J. S. Binkley, M. S. Gordon, D. J. DeFrees, J. A. Pople, “Self-consistent molecular orbital methods. XXIII. A polarization-type basis set for second-row elements” *J. Chem. Phys.* **1982**, *77*, 3654–3665.

[12] M. J. Frisch, G. W. Trucks, H. B. Schlegel, G. E. Scuseria, M. A. Robb, J. R. Cheeseman, G. Scalmani, V. Barone, G. A. Petersson, H. Nakatsuji, X. Li, M. Caricato, A. V. Marenich, J. Bloino, B. G. Janesko, R. Gomperts, B.Mennucci, H. P. Hratchian, J. V. Ortiz, A. F.  Izmaylov, J. L. Sonnenberg, D. Williams-Young, F. Ding, F. Lipparini, F. Egidi, J. Goings, B. Peng, A. Petrone, T. Henderson, D. Ranasinghe, V. G.  Zakrzewski, J. Gao, N. Rega, G. Zheng, W. Liang, M. Hada, M.Ehara, K. Toyota, R. Fukuda, J. Hasegawa, M. Ishida, T. Nakajima, Y. Honda, O.  Kitao, H. Nakai, T. Vreven, K. Throssell, Jr. J. A. Montgomery, J. E. Peralta, F. Ogliaro, M.J. Bearpark, J. J. Heyd, E. N. Brothers, K. N. Kudin, V. N. Staroverov, T. A. Keith, R.Kobayashi, J. Normand, K. Raghavachari, A. P. Rendell, J. C. Burant, S. S. Iyengar, J.Tomasi, M. Cossi, J. M.  Millam, M. Klene, C. Adamo, R. Cammi, J. W. Ochterski, R.L. Martin, K. Morokuma, O. Farkas, J. B. Foresman, D. J. Fox, **2017**, Gaussian, Inc.

[13] K. Fukui, “The path of chemical reactions - the IRC approach” *Acc. Chem. Res.* **1981**, *14*, 363–368.

[14] G. Scalmani, M. J. Frisch, “Continuous surface charge polarizable continuum models of solvation. I. General formalism” *J. Chem. Phys.* **2010**, *132*, DOI 10.1063/1.3359469.

[15] L. Yuanhe, “liyuanhe211/Energy_Diagram_Plotter_CDXML: Energy Diagram Plotter CDXML 3.4.2 (3.4.2).” *Zenodo*, **2022**, DOI: 10.5281/zenodo.6399320.

[16] E. D. Glendening, C. R. Landis, F. Weinhold, “*NBO 7.0* : New vistas in localized and delocalized chemical bonding theory” *J. Comput. Chem.* **2019**, *40*, 2234–2241.

[17] T. Lu, F. Chen, “Multiwfn: A multifunctional wavefunction analyzer” *J. Comput. Chem.* **2012**, *33*, 580–592.

[18] W. Humphrey, A. Dalke, K. Schulten, “VMD: Visual molecular dynamics” *J. Mol. Graph.* **1996**, *14*, 33–38.

[19] Chemcraft - graphical software for visualization of quantum chemistry computations, 1.8, build 682, Ivanovo, Russia

[20] G. D. Frey, V. Lavallo, B. Donnadieu, W. W. Schoeller, G. Bertrand, “Facile Splitting of Hydrogen and Ammonia by Nucleophilic Activation at a Single Carbon Center” *Science (1979).* **2007**, *316*, 439–441.
